# Carbon neutrality strategies modulate air quality effects on solar power generation

**DOI:** 10.1038/s41467-026-77344-z

**Published:** 2026-09-01

**Authors:** Hongrong Shi, Da Gao, Chenfei Qu, Xiang’ao Xia, Dazhi Yang, Da Zhang, Mengmeng Song, Disong Fu, Baozhu Ge, Hongbin Chen, Yun Zhu, Rong Wang, Junji Cao, Shuxiao Wang, Bin Zhao

**Affiliations:** 1 State Key Laboratory of Atmospheric Environment and Extreme Meteorology, Institute of Atmospheric Physics, Chinese Academy of Sciences, Beijing, China; 2 Key Laboratory of Energy Meteorology, China Meteorological Administration (CMA-EML), Beijing, China; 3 State Key Laboratory of Regional Environment and Sustainability, School of Environment, Tsinghua University, Beijing, China; 4 State Environmental Protection Key Laboratory of Sources and Control of Air Pollution Complex, Beijing, China; 5 Institute of Energy, Environment, and Economy, Tsinghua University, Beijing, China; 6 Laboratory of Middle Atmosphere and Global Environment Observation, Institute of Atmospheric Physics, Chinese Academy of Sciences, Beijing, China; 7 School of Electrical Engineering and Automation, Harbin Institute of Technology, Harbin, China; 8 School of Environment and Energy, South China University of Technology, Guangzhou, China; 9 Shanghai Key Laboratory of Atmospheric Particle Pollution and Prevention (LAP³), Department of Environmental Science and Engineering, Fudan University, Shanghai, China

**Keywords:** Sustainability, Energy economics, Environmental impact

## Abstract

Solar photovoltaic power is essential for achieving carbon neutrality, yet its output is highly sensitive to changes in air quality under future emission pathways. We integrate energy–economy, coupled meteorology–chemistry, and photovoltaic performance models to quantify how alternative carbon-neutrality pathways influence solar power generation via air quality changes in China. Here we show that a pathway emphasizing renewable-energy deployment yields the greatest air-quality improvements, boosting annual photovoltaic power generation by 57,526 ± 10,314 gigawatt-hours (GWh) relative to business-as-usual, equivalent to economic gains of US$5.18 ± 0.93 billion by 2060. These gains are driven primarily by aerosol–cloud interactions rather than aerosol–radiation interactions. In contrast, pathways relying on biomass or carbon capture achieve only about one-third of these gains. The largest gains occur in eastern and southern China, where electricity demand is highest. These findings highlight the importance of co-optimizing decarbonization and air-pollution mitigation to maximize the renewable energy benefits of carbon-neutrality strategies.

## Abstract (web summary)

This study reveals that the choice of carbon-neutrality pathway can determine the future solar energy gains from air-quality improvements. Renewable-focused strategies deliver the largest photovoltaic benefits, with aerosol–cloud interactions driving most of the enhancement.

## Introduction

The global transition to net-zero CO₂ emissions is accelerating to mitigate climate change, with many countries including China committing to peak emissions before 2030 and achieve carbon neutrality by 2060^1–3^. This ambitious target necessitates a fundamental transformation of China’s energy system, where solar photovoltaic (PV) technology is poised to become a cornerstone of decarbonization, given its vast resource potential and declining costs^4,5^.

However, PV system performance is acutely sensitive to atmospheric conditions, particularly aerosol pollution^3,6^. In China, atmospheric aerosols reduced surface solar radiation, depressing annual PV power generation by >20% between 2003 and 2014^7^. Since 2013, stringent clean air policies have significantly improved air quality, with recent studies demonstrating a measurable rebound in surface solar radiation and consequent PV output^8,9^. Observational evidence shows that air pollution can substantially suppress PV generation, with reductions ranging from several percent to over 20% under polluted conditions^10–12^.

As China advances toward carbon neutrality, ongoing emission reductions will significantly modify atmospheric composition and thereby surface solar radiation. Understanding the impacts of these pathway-specific air quality improvements on PV generation is therefore critical. Current projections of solar energy potential predominantly examine broad climate change scenarios, within which evolving aerosol forcing can substantially alter projected changes in surface solar radiation and PV power potential^13–19^. However, pathway-specific air quality effects associated with national decarbonization strategies remain largely unexplored. Although recent work has demonstrated co-benefits of China’s carbon neutrality pledge for both air quality and renewable energy performance^20–22^, it remains unclear how divergent technological pathways—even when achieving comparable CO₂ reduction targets—could yield substantially different air pollutant emissions and, in turn, distinct impacts on PV cell performance^23–25^. This underscores the imperative to quantify how China’s alternative carbon neutrality pathways modulate PV generation through their distinct air quality impacts. This presents particular challenges as it must account for multiple interacting processes stemming from emission control measures that collectively influence PV generation potential. The complex causal chain spans from policy-driven technology deployments to atmospheric chemistry changes and ultimately to solar energy outputs, requiring careful integration across economic, environmental, and energy systems.

In this work, we develop an integrated modeling framework to quantify air quality-PV generation interactions across China’s carbon neutrality pathways. We combine the China-in-Global Energy Model (C-GEM) to generate economy-wide emission trajectories, the Weather Research and Forecasting model coupled with Chemistry (WRF-Chem) to simulate high-resolution aerosol and meteorological fields, and a photovoltaic performance model to estimate power generation. By comparing a baseline scenario (BS) without carbon neutrality constraints with three alternative carbon neutrality pathways characterized by different technological strategies—high renewable (HR), high biomass (HB), and carbon capture and storage (CS)—this study systematically evaluates how pathway-dependent emission reductions alter aerosol loading, surface solar radiation, and ultimately PV performance. This integrated framework enables a comprehensive assessment of the interactions between decarbonization strategies, air quality improvements, and solar energy resources, providing insights into how policy-driven technology choices may shape the future performance of PV systems.

## Results

We calculated the photovoltaic power potential (PV_POT_), a dimensionless value describing the performance of PV cells relative to the nominal power capacity under pathway-specific environmental conditions. PV_POT_, when multiplied by the nominal installed watts of PV power capacity, gives the PV power generation. This was computed using a PV model chain that integrates key meteorological variables from high-resolution WRF-Chem simulations, specifically global horizontal irradiance (GHI), 2-m temperature (T2), and wind speed (WS) at 10 m, with PV system characteristics. Notably, we utilized ensemble downscaled future meteorological fields for three-year simulations, enhancing the robustness of our projections. The WRF-Chem simulations were driven by emissions derived from the C-GEM under each scenario. This integrated approach allows us to quantify the changes in PV power generation resulting from air quality improvements associated with different carbon-neutrality strategies. In this study, we analyze the results based on China’s seven regional power grids, enabling a spatial assessment of variations in PV_POT_ and air quality improvements across China.

### PV power potential changes and their drivers under different carbon-neutrality scenarios

**Fig. 1:**
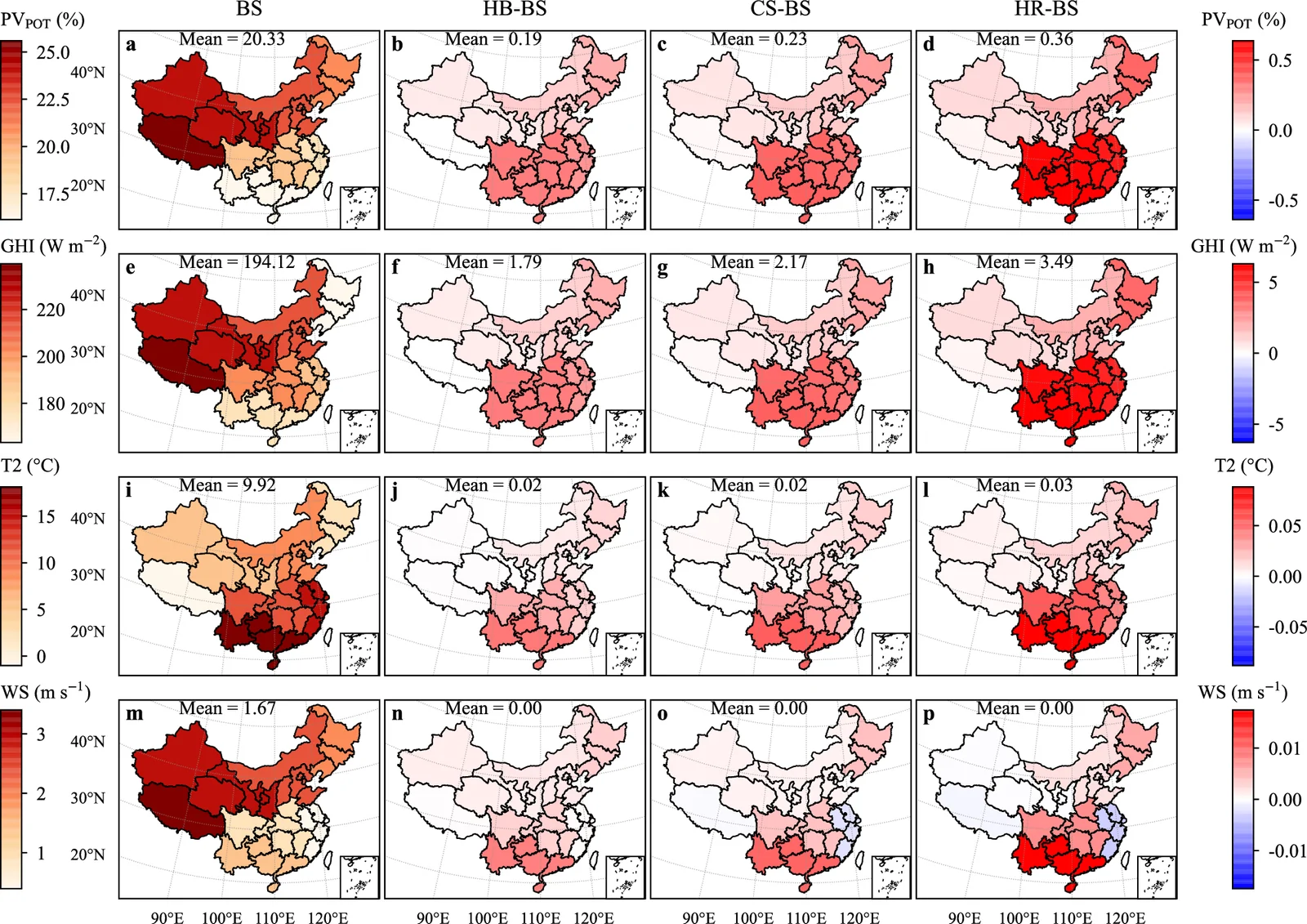
Photovoltaic power potential and key meteorological variables under different carbon-neutrality pathways. Spatial distributions of photovoltaic power potential (PV_POT_) and key meteorological variables under the baseline scenario (BS), and their changes under three carbon-neutrality pathways across China in 2060. **a**–**d**, PV_POT_; **e**–**h**, global horizontal irradiance (GHI); **i**–**l**, 2-m air temperature (T2); and **m**–**p**, wind speed (WS). Within each row, the leftmost panel shows the absolute value under BS, whereas the remaining panels show differences relative to BS under the high-biomass (HB), carbon capture and storage (CS) and high-renewable (HR) pathways, denoted as HB–BS, CS–BS and HR–BS, respectively.

Figure 1 illustrates the spatial distribution of PV_POT_ along with three key factors influencing PV_POT_, GHI, T2, and WS, across China’s seven regional power grids under the BS scenario and the differences between three carbon-neutrality pathways (HB, CS, HR) and the BS for the year 2060. In the BS scenario, the northwestern region of China exhibits the highest PV_POT_. The national average PV_POT_ in the BS scenario is 20.2%. Under the three carbon neutrality pathways, there is a quantifiable increase in PV_POT_ across most regions. The HR shows the largest average increase of 0.36% ± 0.07% compared to BS, where the uncertainty (±1 standard deviation; see the Uncertainty analysis section in the Methods) reflects the uncertainty in radiation simulation from WRF-Chem, followed by the CS with an increase of 0.23% ± 0.05%, and the HB with an increase of 0.19% ± 0.04%. The most significant PV_POT_ improvements are observed in the eastern and central regions (up to 0.5% in HR), which historically have been major emission sources and heavily polluted areas in China. Under the HR scenario, a substantial number of grid cells exhibit significant increases with false discovery rate control, particularly across eastern, central, and southern China, supporting the robustness of the regional-mean enhancement, but substantial spatial heterogeneity (Supplementary Figs. 1, 2 and Supplementary Note 1). Under the carbon neutrality pathways, these regions experience more pronounced air quality improvements due to stringent emission reduction measures^23^. This regional variation highlights the disproportionate benefits of air quality improvements in traditionally more polluted areas.

**Fig. 2:**
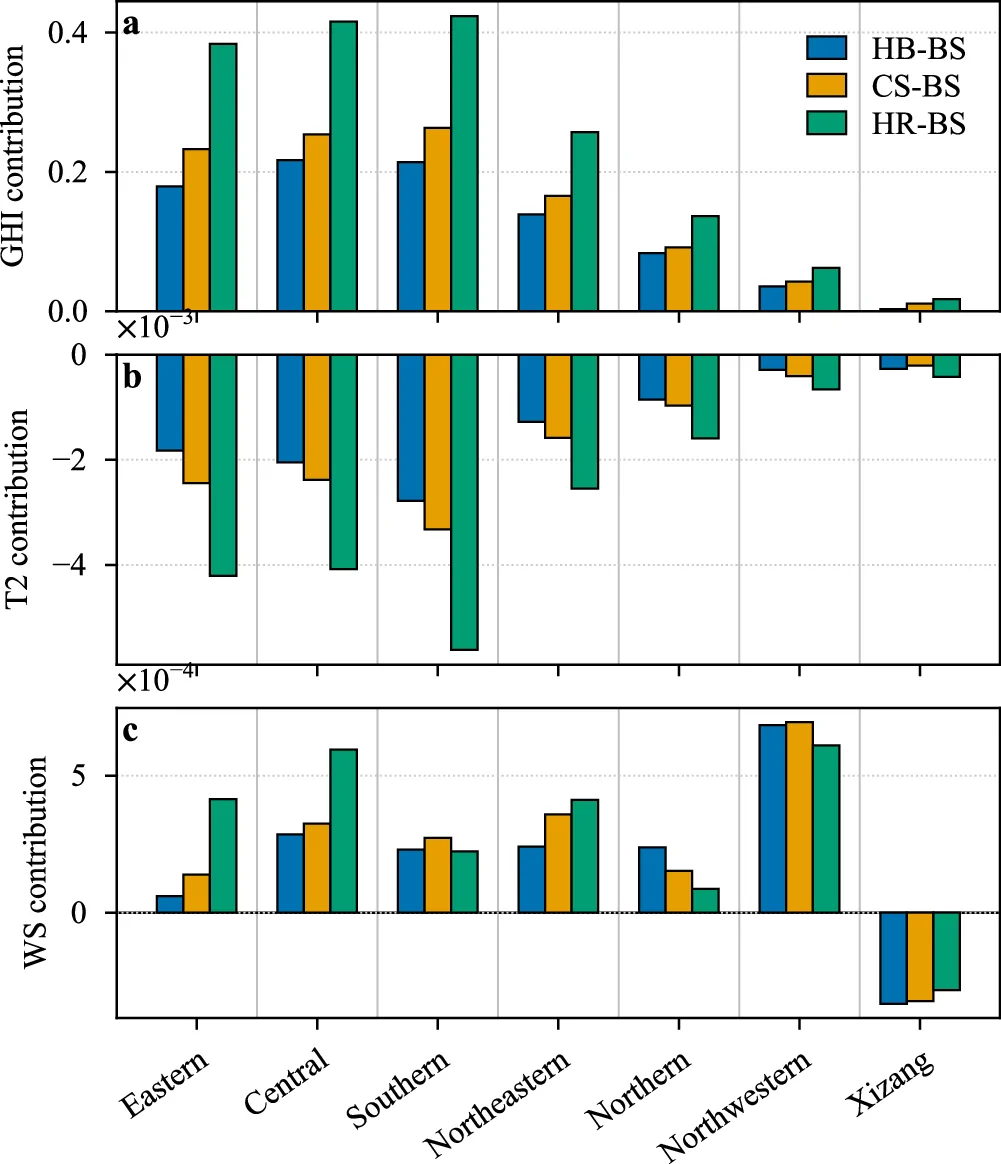
Contributions of key meteorological variables to changes in photovoltaic power potential across China’s seven regional power grids. Contributions (%) of **a** global horizontal irradiance (GHI), **b** 2-m air temperature (T2), and **c** wind speed (WS) to changes in photovoltaic power potential (PV_POT_) under the high-biomass (HB), carbon capture and storage (CS) and high-renewable (HR) pathways relative to the baseline scenario (BS) in 2060. The seven power-grid regions are Eastern, Central, Southern, Northeastern, Northern, Northwestern and Xizang.

To gain a quantitative understanding of the factors driving changes in PV_POT_, we quantified the contributions of GHI, T2, and WS to the changes in PV_POT_ under different carbon neutrality pathways relative to the BS scenario. We applied a Shapley value–based attribution to decompose changes in PV_POT_ (see the Contribution of GHI, T2, and WS to PV_POT_ changes section in the “Methods”). The method evaluates all factorial combinations of the three variables between baseline and scenario conditions, yielding a path-independent attribution that accounts for nonlinearity and interactions. Figure 2 presents the decomposed contributions of GHI, T2, and WS to the changes in PV_POT_ across seven regional power grids. It reveals that changes in GHI serve as the primary driver for the total changes in PV_POT_ in all regions with a dominant positive impact. The most pronounced GHI contributions are observed in Eastern, Central, and Southern China, while regions such as Northern, Northwestern, and Xizang show smaller contributions. In contrast, the impacts of T2 and WS are considerably smaller. The contribution of T2 is consistently negative across all regions, reflecting the temperature-induced reduction in photovoltaic conversion efficiency^26^. This negative effect becomes more pronounced under the HR scenario, particularly in the Southern and Central grids. Moreover, the temperature increase in our simulations is partly associated with enhanced surface solar radiation, which leads to additional surface heating. The contribution of WS is two to three orders of magnitude smaller than that of GHI and can therefore be safely neglected in the attribution analysis.

**Fig. 3:**
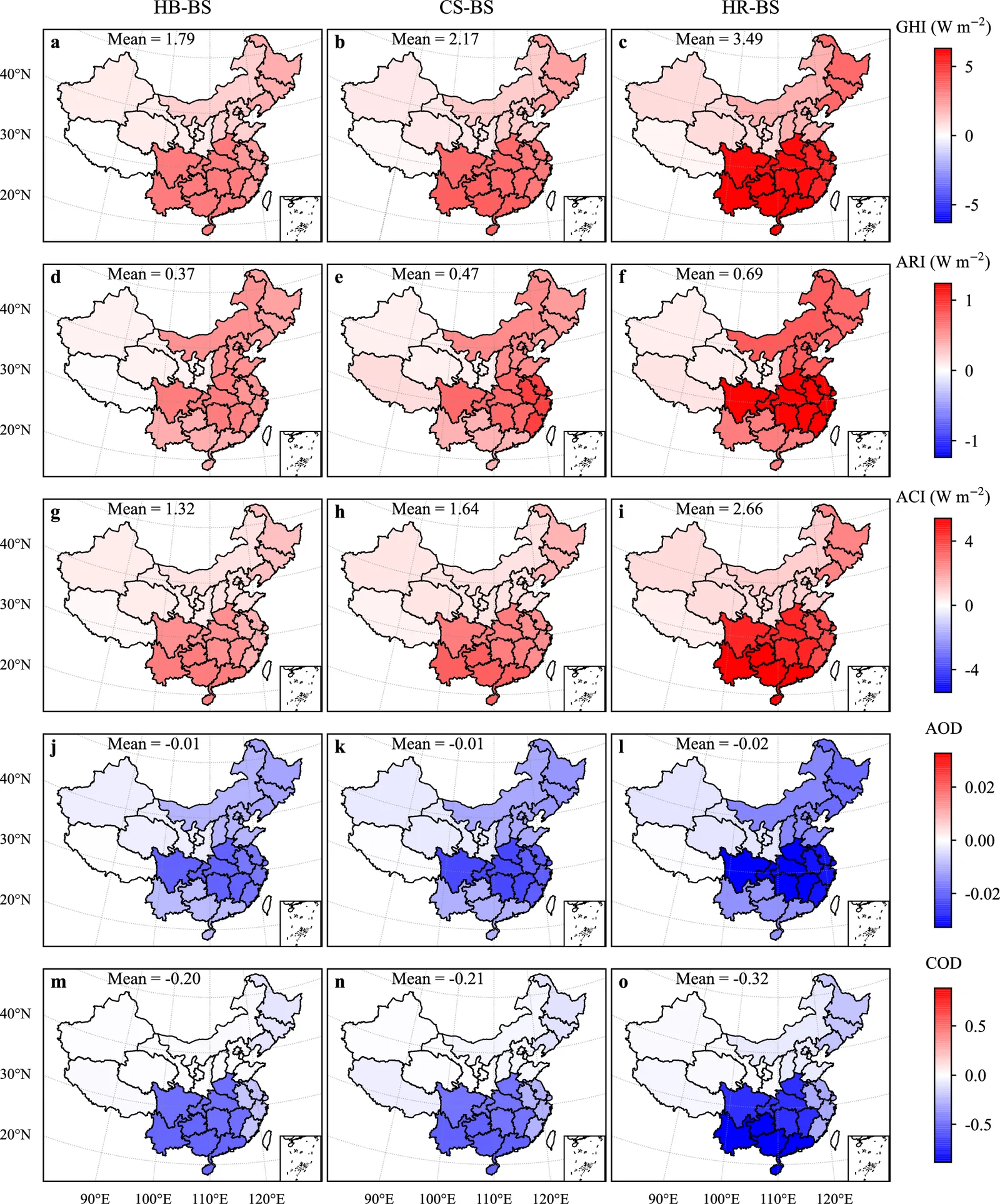
Spatial distributions of key atmospheric variables influencing changes in global horizontal irradiance. Spatial distributions of changes in global horizontal irradiance (GHI) and the associated contributions and atmospheric variables under three carbon-neutrality pathways relative to the baseline scenario (BS) across China in 2060. **a**–**c** Changes in GHI; **d**–**f** contributions from aerosol–radiation interactions (ARI); **g**–**i** contributions from aerosol–cloud interactions (ACI); **j**–**l** changes in aerosol optical depth (AOD); and **m**–**o** changes in cloud optical depth (COD). Within each row, the panels show the high-biomass (HB), carbon capture and storage (CS) and high-renewable (HR) pathways, respectively. Regional boundaries indicate China’s seven regional power grids.

Given that GHI contributes most significantly to PV_POT_ among three meteorological parameters, we need to further understand the mechanisms driving the increase in GHI. To explicitly separate the radiative effects arising from aerosol–radiation interactions (ARI) and aerosol–cloud interactions (ACI), we conducted paired sensitivity simulations within the WRF-Chem framework (see the Attribution of scenario-induced GHI changes section in the Methods). Figure 3 presents the spatial distribution of total GHI changes (ΔGHI) and their decomposed contributions from ARI (ΔGHI_ARI_) and ACI (ΔGHI_ACI_). Interestingly, the process-level decomposition reveals a clear dominance of ACI. While ΔGHI_ARI_ shows a modest positive contribution (domain means of 0.37, 0.47, and 0.69 W m⁻² for HB, CS, and HR, respectively), the ΔGHI_ACI_ component is substantially larger, contributing 1.32, 1.64, and 2.66 W m⁻². In all scenarios, ACI accounts for approximately three-quarters of the total radiation enhancement. The fractional contribution of ACI to total ΔGHI increases progressively from HB to CS and is largest under the HR, indicating a strengthening dominance of indirect aerosol effects under more aggressive emission reductions.

Further insight is provided by the consistent decrease in Aerosol Optical Depth (AOD) and Cloud Optical Depth (COD) across all scenarios relative to BS, especially prominent in eastern and central regions. The spatial distribution of AOD and COD reduction shows a strong correlation with the contribution from ARI and ACI, respectively. The combined spatial distribution of AOD and COD reductions is consistent with the distribution of GHI enhancement (Supplementary Fig. 3), especially in southern, eastern, and central China. The substantial COD reduction strongly suggests the influence of ACI on cloud properties. Fewer aerosols are likely to lead to decreased cloud droplet number concentrations (NDROP) and a reduction in liquid water path (LWP) (Supplementary Fig. 4), potentially reducing cloud albedo and allowing more solar radiation to reach the surface.

### PV gains from air quality improvement under carbon neutrality scenarios

To quantify the benefits of the pathway-specific PV power generation increase from air-quality improvements in 2060, we calculated the additional annual power generation and its corresponding monetary value.

**Fig. 4:**
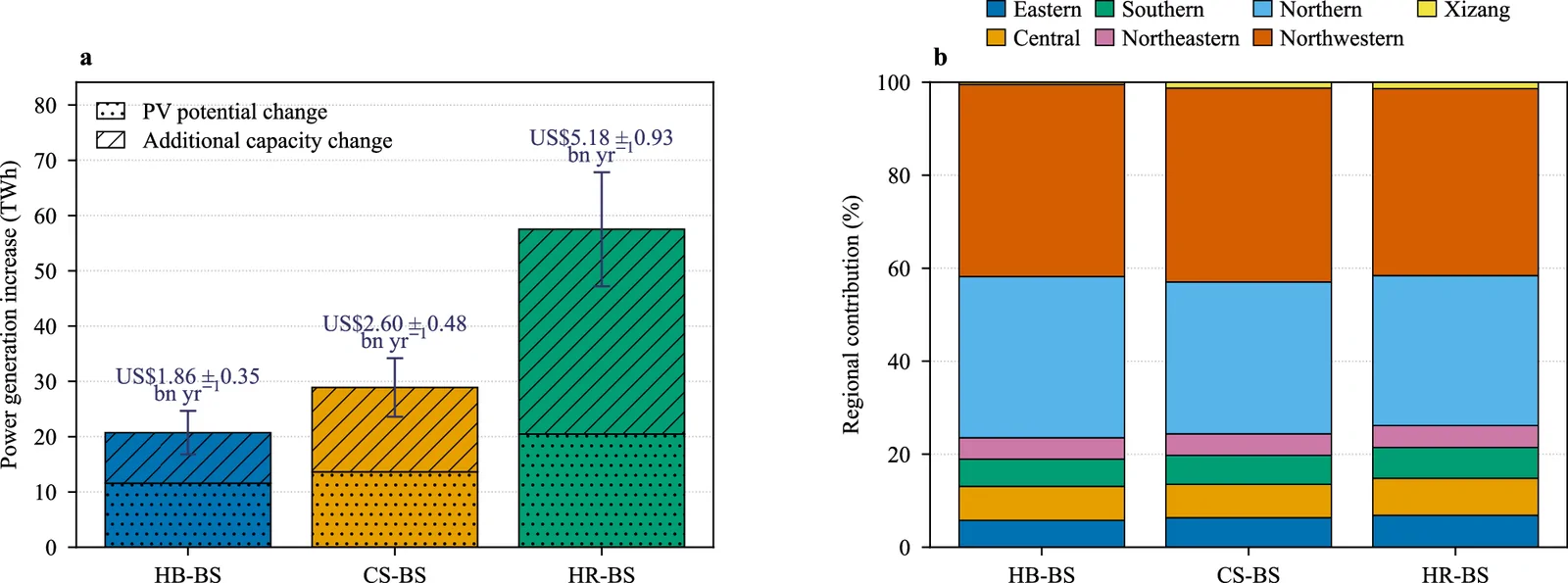
Pathway-specific photovoltaic power gains from air-quality improvements and their regional distributions. **a** Decomposition of increases in photovoltaic (PV) power generation into contributions from changes in photovoltaic power potential (PV_POT_) and additional installed capacity. Economic gains are expressed in billions of US dollars per year (US$ bn yr⁻¹), and error bars represent one standard deviation (SD). **b** Regional variations in pathway-specific PV power-generation increases across the Eastern, Central, Southern, Northeastern, Northern, Northwestern and Xizang power-grid regions under the high-biomass (HB), carbon capture and storage (CS) and high-renewable (HR) pathways relative to the baseline scenario (BS) in 2060.

Figure 4a illustrates the pathway-specific increases in PV power generation associated with air-quality improvements. Total increases in PV power generation reach 20,717 ± 3937 gigawatt-hours (GWh), 28,899 ± 5288 GWh, and 57,526 ± 10,314 GWh under HB, CS, and HR, respectively, in 2060. As emission-reduction efforts intensify (i.e., from HB to CS and further to HR), improvements in solar resource availability become more pronounced, leading to larger realized gains in PV power generation. These totals reflect both the direct enhancement of PV output under baseline installed capacity due to improved PV_POT_ and an additional synergistic benefit arising when enhanced solar resource conditions coincide with pathway-specific PV capacity expansion (see the Estimation of PV power generation gains from improved air quality section in the “Methods”). For context, the additional 57,526 GWh annual PV generation under the HR pathway is substantial: it is roughly equivalent to the total annual electricity consumption of the Beijing-Tianjin-Hebei (Jing-Jin-Ji) region, a major national economic and urban core zone in northern China in 2025^27^. Importantly, this significant gain is achieved as a direct co-benefit of decarbonization pathways, without requiring additional technological upgrades to PV panels, contrasting with the considerable economic inputs typically needed for advances in PV panel conversion efficiency. Using an average electricity price of US$0.09 kWh^−1^, which reflects current market rates in China (approximately US$0.084 kWh^−1^ for residential and US$0.089 kWh^−1^ for industrial users as of 2021)^28^, we estimated the economic value of this additional generation. Driven by the dual objectives of market reform and decarbonization, these rates are projected to rise in the future due to factors such as GDP growth and the mandatory reduction in energy intensity^28^. Additionally, recent system-level modeling studies suggest that electricity supply costs will continue to rise under carbon neutrality pathways, reaching higher levels by 2050 due to large-scale renewable energy deployment, grid expansion, and system integration requirements^29^. Therefore, the adopted value of US$0.09 kWh^−1^ represents a conservative estimate within the projected range of future electricity costs. For the HR pathway, this amounts to approximately US$5.18 ± 0.93 billion per year in 2060. Similar calculations were performed for the CS and HB pathways, yielding additional annual revenues of US$2.60 ± 0.48 billion and US$1.86 ± 0.35 billion, respectively.

The results highlight the substantial economic benefits that can be derived from the improved PV power generation resulting from aerosol emission reductions under different carbon neutrality pathways, with the HR pathway offering the most significant gains. Moreover, this synergistic effect creates a positive feedback loop, further enhancing the role of solar energy in the HR pathway. The HR pathway, with its strong focus on renewable energy and favorable PV_POT_, exhibits a reinforcing effect that significantly boosts solar power generation. This positive feedback loop highlights the potential for synergistic interactions between renewable energy deployment expansion and improved solar resources under ambitious decarbonization scenarios.

Focusing on the regional variations in PV power generation increases, Fig. 4b presents the percentage contribution of seven power grid regions to the national total increase in PV power generation under HB/CS/HR relative to BS in 2060. The results reveal a consistent increase in PV power generation across all regions under all carbon-neutrality pathways compared to the BS. More importantly, regions with higher electricity demand, predominantly located in central and eastern China, exhibit a more substantial increase in PV power generation, especially under scenarios with greater emission reductions (i.e., from HB to CS and further to HR). This concerted effort towards aggressive emission reductions not only maximizes overall PV power generation but also has significant implications for specific regions. This amplified increase is particularly advantageous for these densely populated and industrialized areas, which typically face greater energy consumption pressures. This trend is primarily attributable to the significantly higher baseline aerosol concentrations and larger absolute reductions achieved in these regions under intensive mitigation pathways, leading to a more pronounced improvement in surface solar radiation. In addition, the high density of existing and planned PV installations in these areas means that such improvements translate directly into greater overall energy output and economic value, reinforcing their strategic importance. These regional variations underscore the importance of considering synergistic interactions between renewable energy deployment expansion and air quality improvement when evaluating the potential impacts of carbon-neutral pathways on renewable energy generation capacities across diverse geographical contexts.

## Discussion

This study quantitatively estimated the impact of emission-driven air quality changes under three distinct carbon neutrality pathways (HB, CS, and HR) on China’s PV power generation in 2060, compared to a BS that assumes no specific carbon neutrality targets. Our comprehensive findings reveal that pathway-specific air quality enhancements significantly improve PV_POT_ across China, with strong spatial heterogeneity. Specifically, the different carbon neutrality pathways lead to varying degrees of PV power generation benefits, which are primarily mediated by increases in surface solar radiation.

For instance, under the HR pathway, the national-average PV_POT_ increases by 0.36 percentage points, from 20.2% in the BS scenario to about 20.6%, equivalent to a ~2% relative increase. To contextualize this gain within the broader trajectory of solar technology, we compare it against industry benchmarks from the International Technology Roadmap for Photovoltaic (ITRPV). Over the past decade (2010–2020), standard mono-crystalline module efficiencies improved from approximately 15–20%^30^, driven by the maturation of Passivated Emitter Rear Cell (PERC) technology, representing a decade-long average annual increase of 0.5% per year. Looking toward 2030, the roadmap projects further systematic enhancements: average module efficiencies are expected to reach 22.5–24.0% through the adoption of Tunnel Oxide Passivated Contact (TOPCon) and Heterojunction (HJT) architectures, with perovskite/c-Si tandem cells creating a pathway toward 30% efficiency^31,32^, with average annual gains of 0.3–1%. Crucially, modern PV progress extends beyond cell efficiency to include system-level innovations that boost annual energy yield. Key innovations including single-axis tracking, bifacial modules, and optimized direct-current to alternating-current (DC/AC) inverter ratios (rising from 1.2 to 1.7) collectively boost annual energy yield. When aggregated, the combined effect of material and system-level advancements yields an overall system yield improvement of 0.8–1.2% per year according to industry benchmarks^33^. This comparison indicates that the PV_POT_ gain from air quality improvements alone is remarkably material. Crucially, this benefit is realized without any direct investment in PV panel or system upgrades, highlighting a significant co-benefit of carbon reduction pathways.

Importantly, while many PV studies account for the direct radiative effects of aerosols^6,34,35^, they often overlook the more complex and frequently dominant role of aerosol-cloud interactions in modulating surface solar radiation^36,37^. Our findings suggest that these indirect effects play a central role in shaping solar resource availability under cleaner atmospheric conditions. Future PV modeling efforts must therefore incorporate aerosol-cloud interactions to avoid underestimating the true potential of PV generation in a decarbonizing world.

Regional analysis reveals a critical insight: economically developed regions with high energy demand—particularly central and eastern China—are projected to experience the largest increases in realized PV generation under pathways associated with improved atmospheric conditions. Zhuo et al.^28^ emphasize that achieving China’s net-zero emissions target will require substantial strengthening of east-west electricity transmission, which is likely to involve considerable cost increases. However, the projected increases in PV_POT_ under all three carbon neutrality pathways—especially those driven by reductions in aerosol concentrations in eastern China—create favorable solar resource conditions for distributed PV systems. This not only boosts local PV generation but also contributes to enhanced energy self-sufficiency, reducing reliance on external energy sources and reinforcing regional energy independence and resilience. These findings underscore the necessity of adaptive, region-specific energy planning and policy-making. Tailored strategies are essential to account for the spatial heterogeneity of air quality impacts on solar energy potential across the country. Failing to address these regional disparities could result in suboptimal energy policies and an underestimation of the role that solar power can play in ensuring regional energy security. Future research should focus on developing optimal strategies for integrating additional PV capacity into existing grid infrastructures, while also evaluating the associated economic and environmental trade-offs at the regional level.

## Methods

### Energy-economic model and four scenarios for China’s carbon-neutrality pathways

We utilize the C-GEM, a multi-regional recursive-dynamic computable general equilibrium model, to assess China-specific carbon neutrality pathways and associated emission trajectories toward the national 2060 carbon-neutrality target^29,38^. C-GEM integrates economic activities, energy flows, and CO_2_ emissions across 21 sectors while capturing interactions among producers, households, and government policies. The model is calibrated using China’s 2020 input-output tables, energy balance statistics, and supplementary data from the World Bank and International Energy Agency (IEA), incorporating key decarbonization technologies—including wind, solar, nuclear power, electric vehicles, and carbon capture and storage^38–40^. We examine four China-specific scenarios: (1) a BS without carbon neutrality target in 2060; and three carbon-neutral pathways achieving net-zero emissions by 2060 through distinct technological emphases—CS featuring significant 20% fossil fuel reliance with Carbon Capture, Utilization, and Storage, HB with expanded bioenergy use featuring 9% biomass energy share, and HR dominated by wind and solar power accounting for 70% of primary energy. The key socio-economic characteristics and sectoral activity levels for these pathways, including CO_2_ capture amounts by technology, electrification rates, and energy mix compositions, are quantitatively summarized in Supplementary Table 1. Importantly, the design of the three carbon neutrality scenarios follows a key principle: all scenarios meet the nationally mandated target of 80% non-fossil energy share by 2060, while maximizing the differentiation of technology pathways under this constraint. Additional details of the scenario designs are provided in Gao et al.^23^. Emissions of SO_2_, NO_x_, volatile organic compounds (VOCs), NH_3_, PM_2.5_, black carbon, and organic carbon are derived by integrating sector-specific energy consumption data from the C-GEM with technology-dependent emission factors for 31 regions of mainland China from the Air Benefit and Cost and Attainment Assessment System emission inventory (ABaCAS-EI)^41^, assuming gradual emission factor reductions from 2020 to 2035 followed by stabilization. Under these pathway-specific assumptions, different carbon neutrality strategies produce distinct emission trajectories^23^. For instance, the HR exhibits the lowest emission levels due to its high electrification rate (95%) and non-fossil share (97%). Compared to the BS, HR leads to substantial decreases in SO_2_, PM_2.5_, NO_x_, and VOCs emissions by 68%, 57%, 53%, and 28%, respectively. The HB exhibits a weaker reduction in PM_2.5_ (only 28% lower than BS) due to the increased utilization of biomass in the domestic combustion sector. The CS represents an intermediate level, with reduction ratios of 55%, 37%, 33%, and 21% for SO_2_, PM_2.5_, NO_x_, and VOCs, respectively. These quantified sectoral differences provide the physical basis for the spatially heterogeneous aerosol responses and subsequent PV_POT_ changes analyzed in this study.

### Air-quality model and simulations

We employ WRF-Chem to assess aerosol concentrations and meteorological parameters under various carbon neutrality scenarios. The WRF-Chem simulation is conducted using a single domain covering the entire land area of China with a horizontal grid spacing of 27 km. The model uses 24 vertical layers extending from the surface to 50 hPa, with enhanced resolution within the planetary boundary layer. Aerosol direct radiative effects as well as the first and second aerosol indirect effects on grid-scale clouds are included. The physical and chemical parameterizations used in this study are summarized in Supplementary Table 2 and are consistent with those described by Gao et al.^42,43^. This model configuration has previously been validated and shown to represent the complex aerosol-cloud interactions processes that regulate surface solar irradiance^40,41^.

The anthropogenic emissions from the C-GEM/ABaCAS-EI framework were downscaled to a 27 km grid. To generate gridded hourly emission files for WRF-Chem simulations, emissions were temporally allocated (monthly, weekly, and hourly) based on activity data^44–47^, and spatially distributed using proxies derived from county-level GDP, high-resolution population data, and road network maps^48^. To ensure spatial reliability, the simulation results were validated against nationwide PM_2.5_ observations for the base year of 2021. The spatial patterns of simulated PM_2.5_ align well with observations (Supplementary Fig. 7 in ref. ^23^), and statistical performance metrics (normalized mean bias (NMB) < 25%, Supplementary Table 2 in ref. ^23^) meet established modeling standards, confirming that the emission allocation captures the essential spatial characteristics required for regional radiative analysis.

Future meteorological initial and boundary conditions were derived from a bias-corrected ensemble mean of 18 global climate models from the Coupled Model Intercomparison Project Phase 6 (CMIP6) under the Shared Socioeconomic Pathway 1–2.6 (SSP126) scenario^49^. This multi-model ensemble dataset was specifically developed for regional dynamical downscaling and has been shown to effectively reduce uncertainties associated with individual model climate sensitivity and large-scale circulation biases. By employing ensemble-mean climate forcing, the representativeness and robustness of projected future meteorological conditions are substantially improved, thereby providing a reliable basis for regional air-quality and PV-impact assessments.

Future chemical initial and boundary conditions were constructed by scaling present-day global chemical fields using concentration ratios between 2021 and 2060 under SSP126^23^. The reduction ratio was obtained from the changes in concentrations of PM_2.5_ and its components from 2021 to 2060 under SSP126 in Institut Pierre-Simon Laplace Climate Model version 6A with low resolution Model (https://esgf-node.llnl.gov/search/cmip6/). This proportional-scaling approach preserves the large-scale spatial structure of chemical fields while ensuring consistency with the global low-emission background represented by SSP126.

Moreover, we performed 3-year WRF-Chem simulation covering 2059–2061 to reduce the impact of internal climate variability. This approach allowed us to project air quality and meteorological changes under different carbon neutrality pathways, accounting for regional variations in aerosol and atmospheric conditions, and thus more accurately assess their impact on PV power generation. For each grid cell, scenario differences were evaluated using a paired t-test based on annual means from three-year simulations (2059–2061). To account for multiple comparisons in spatial fields, the false discovery rate (FDR) was controlled using the Benjamini–Hochberg procedure with *q* = 0.05 (details in Supplementary Note 1). Grid cells passing the FDR criterion are indicated in the spatial figures in Supplementary Fig. 1.

The robustness of the projected enhancements in GHI and PV_POT_ was further evaluated using an independent climate-forcing dataset and an additional simulation year. Results indicate that both the spatial patterns and regional responses remain largely unchanged, supporting the stability of the conclusions presented here (Supplementary Note 2; Supplementary Figs. 6–9). While this timeframe may not capture decadal climatological shifts, it provides a robust basis for isolating the radiative benefits of air quality improvements under diverse carbon-neutrality strategies. Our analysis focuses on relative differences across emission scenarios rather than absolute meteorological or climate states, so the influence of interannual meteorological variability on our key conclusions is greatly minimized. According to our literature review^24,50,51^, the combination of an 18-model ensemble and a 3-year simulation period represents a more robust approach to addressing natural variability compared to most existing studies of a similar nature.

To further ensure the robustness of WRF-Chem in PV-related assessments, we conducted an additional evaluation of the model’s ability to simulate GHI, a key input for PV_POT_. We performed a comprehensive spatial comparison of WRF-Chem simulated GHI against historical ground-based observations^52^ across China for the year 2021. After applying quality control to the surface measurements, 132 observation stations were retained for analysis. The spatial distribution of model performance statistics, including mean bias error (MBE), root mean square error (RMSE), relative MBE (rMBE), and relative RMSE (rRMSE), across China’s seven grid power regions for 2021 is presented in Supplementary Table 3 and Note 3. The WRF-Chem simulations show a consistent positive bias in GHI, with rMBE values falling within a narrow range of 8.6–11.5% across the seven grid regions. While absolute GHI values in future projections may remain biased, the analysis of inter-scenario differences is less sensitive to such systematic biases.

### PV model chains for estimating PV_POT_ and power generation

We employ the pvlib-python package (Sandia National Laboratories)^53^ to estimate PV_POT_ from WRF-Chem simulated GHI and other meteorological parameters (T2 and WS). This physics-based approach, which better captures the nonlinear irradiance-to-power conversion compared to traditional regression models, systematically converts GHI to PV power output through three key stages^54,55^: (1) Radiation decomposition: decomposing GHI into direct and diffuse components using the DISC model, a quasi-physical algorithm widely adopted by the National Solar Radiation Database (NSRDB) for its robust performance in partitioning radiation based on the clearness index and solar position, (2) Transposition and optical losses, utilizing the Perez model to calculate Plane-of-Array (POA) irradiance, while incorporating the Xie model for reflection losses and Sandia Array Performance Model (SAPM) for spectral correction; and (3) Power output calculation, where cell temperature was estimated via the SAPM temperature model, and final DC power was determined using the PVWatts model. The open-source pvlib toolkit provides validated models for each conversion stage^52^, enabling robust simulation of PV system performance under varying atmospheric conditions. Previous studies have already quantified the historical enhancement of PV electricity generation associated with improved air quality in China following the Clean Air Action Plan, demonstrating substantial increases in solar radiation and PV output since 2013^56,57^. Building upon this established modeling framework, the present study focuses on quantifying how future emission pathways influence PV generation through air-quality-mediated changes in solar radiation.

PV power generation is calculated as the product of a dimensionless magnitude, PV_POT_, and the nominal installed watts of PV power capacity. PV_POT_, as defined in this study^14^, represents the performance of PV cells relative to their nominal power capacity under simulated ambient conditions (defined here as the pathway-specific meteorological conditions).

Unlike previous studies^13^, this research utilizes hourly meteorological data from the WRF-Chem model to calculate PV_POT_ for each hour and grid cell. Annual average PV_POT_ for each region under each carbon neutrality pathway is then obtained by averaging over time and region. Finally, annual PV power generation for each region is calculated by multiplying the annual average PV_POT_ by the projected PV installed capacity for each region in 2060 (see Table 1). This approach allows for a more granular assessment of the impact of air quality changes under different carbon neutrality pathways on PV power generation.

**Table 1:** Projected photovoltaic installed capacity across China’s seven regional power grids in 2060

Power Grid Region	BS	HB	CS	HR
Eastern	28	51	60	79
Central	29	53	62	84
Southern	25	43	52	68
Northeastern	29	52	62	81
Northern	377	675	802	1062
Northwestern	1019	1821	2160	2861
Xizang	124	222	263	348
National	1631	2917	3461	4583

Installed photovoltaic (PV) capacity is expressed in gigawatts (GW) for the baseline scenario (BS) and three carbon-neutrality pathways: the high-biomass (HB), carbon capture and storage (CS) and high-renewable (HR) scenarios.

Specifically, we use the pvlib.pvsystem.pvwatts_dc function to calculate the DC power output (${P}_{{{{\rm{dc}}}}}$), as described in Eq. (1).

1 $${P}_{{\mbox{dc}}}=\frac{{G}_{{\mbox{poaeff}}}}{1000}{P}_{{\mbox{dc}}0}\left(1+{\gamma }_{{\mbox{pdc}}}\left({T}_{{\mbox{cell}}}-{T}_{{\mbox{ref}}}\right)\right)$$

where ${G}_{{{{\rm{poaeff}}}}}$ is the effective plane-of-array irradiance (W m^-^²), accounting for angle-of-incidence losses; ${T}_{{{{\rm{cell}}}}}$ is the PV cell temperature (°C); ${P}_{{{{\rm{dc}}}}0}$ is the rated DC power of the module (W); ${\gamma }_{{{{\rm{pdc}}}}}$ is the temperature coefficient of power (°C^−1^), typically range from −0.002 to −0.005 °C^−1^; and ${T}_{{{{\rm{ref}}}}}$ is the reference cell temperature, defined as 25 °C in the PVWatts model.

PV_POT_ is calculated as Eq. (2).

2 $${\mbox{P}}{{\mbox{V}}}_{{\mbox{POT}}}=\frac{{P}_{{\mbox{dc}}}}{{{P}_{{\mbox{dc}}}}_{{\mbox{STC}}}}$$

where ${P}_{{{{\rm{dc}}}}}$ is the DC power output (W) calculated using the pvlib.pvsystem.pvwatts_dc function. ${{P}_{{\mbox{dc}}}}_{{\mbox{STC}}}$ is the DC power under standard test conditions (STC). In this study, we assume a nominal installed capacity of 1000 W, thus ${{P}_{{\mbox{dc}}}}_{{\mbox{STC}}}$ = 1000 W.

### Contributions of GHI, T2 and WS to PV_POT_ changes

PV output responds nonlinearly to GHI, T2, and WS^58^, traditional one-at-a-time approaches cannot provide a path-independent attribution of their contributions^59,60^. Thus, to quantitatively analyze the contribution of GHI, T2, and WS to changes in PV_POT_, a Shapley value–based decomposition method is employed in this study^61^. PV_POT_ is a nonlinear function of meteorological drivers, as described in Eq. (3).

3 $${{{\rm{P}}}}{{{{\rm{V}}}}}_{{{{\rm{POT}}}}}=f\left({{{\rm{GHI}}}},{{{\rm{T}}}}2,{{{\rm{WS}}}}\right)$$

where f represents the full pvlib-based photovoltaic performance model chain.

For a specific driver i∈N (here, *N* = {GHI, T2, WS}), its Shapley value ${\phi }_{i}$ representing its average marginal contribution to the total PV_POT_ change ($\Delta {{{{\rm{PV}}}}}_{{{{\rm{POT}}}}}$), is defined as Eq. (4).

4 $${\phi }_{i}={\sum}_{S\subseteq N\,\setminus \{i\}}\frac{\left|S\right|!\left(\left|N\right|-\left|S\right|-1\right)!}{\left|N\right|!}\left[f\left(S\cup \{i\}\right)-f\left(S\right)\right]$$

where *S* denotes any subset of variables excluding *i*, and ∣*N*∣ = 3 in this study. This framework ensures additive closure, as expressed in Eq. (5).

5 $$\Delta {{{{\rm{PV}}}}}_{{{{\rm{POT}}}}}={\phi }_{{\mbox{GHI}}}+{\phi }_{{{{\rm{T}}}}2}+{\phi }_{{{{\rm{WS}}}}}$$

For each carbon-neutrality pathway (HB, CS, HR), all ${2}^{{|N|}}=8$ factorial combinations of baseline and scenario meteorological variables were constructed. PV_POT_ was evaluated for each combination using the pvlib model chain, and Shapley contributions were computed grid-point-wise through explicit enumeration of all marginal contributions.

### Attribution of scenario-induced GHI changes

In this study, all baseline and carbon-neutral pathway simulations were conducted using the standard WRF-Chem configuration, in which both ARI and ACI are enabled. For clarity, we refer to this standard configuration as the CTRL simulation. To isolate the contributions of ACI and ARI to scenario-induced changes in GHI, we performed a set of paired sensitivity experiments. Two sensitivity experiments were conducted for each scenario, adopting the process-level separation approach^42^: (1) ACI-off experiment (ARI-on), in which aerosol–cloud coupling in cloud microphysics was disabled. The difference between the CTRL and ACI-off simulations therefore represents the radiative contribution arising specifically from aerosol–cloud interactions ($\Delta {{{\rm{GH}}}}{{{{\rm{I}}}}}_{{{{\rm{ACI}}}}}$). (2) ARI-off experiment (ACI-on), in which aerosol–radiation coupling in the radiative transfer scheme was suppressed. The difference between the CTRL and ARI-off simulations thus isolates the radiative contribution attributable to aerosol–radiation interactions ($\Delta {{{\rm{GH}}}}{{{{\rm{I}}}}}_{{{{\rm{ARI}}}}}$). Under this differential framework, the total scenario-induced change in GHI can be approximately decomposed into ACI and ARI contributions.

Our attribution focuses on scenario-induced increments relative to the BS scenario, rather than absolute aerosol effects. For each future carbon-neutral scenario X (HR, HB, and CS), the total change in GHI relative to BS is defined in Eq. (6).

6 $$\Delta{{\mbox{GHI}}}={{{\rm{GH}}}}{{{{\rm{I}}}}}_{{\mbox{CTRL}}}^{{{{\rm{X}}}}}-{{{\rm{GH}}}}{{{{\rm{I}}}}}_{{\mbox{CTRL}}}^{{\mbox{BS}}}$$

The contribution from ACI is diagnosed using Eq. (7).

7 $$\Delta {{{\rm{GH}}}}{{{{\rm{I}}}}}_{{{{\rm{ACI}}}}}=\left({{{\rm{GH}}}}{{{{\rm{I}}}}}_{{{{\rm{CTRL}}}}}^{{{{\rm{X}}}}}-{{{\rm{GH}}}}{{{{\rm{I}}}}}_{{{{\rm{ACloff}}}}}^{{{{\rm{X}}}}}\right)-\left({{{\rm{GH}}}}{{{{\rm{I}}}}}_{{{{\rm{CTRL}}}}}^{{{{\rm{BS}}}}}-{{{\rm{GH}}}}{{{{\rm{I}}}}}_{{{{\rm{ACloff}}}}}^{{{{\rm{BS}}}}}\right)$$

which represents the enhancement of GHI attributable to ACI under scenario X relative to BS.

Similarly, the contribution from ARI is defined in Eq. (8).

8 $$\Delta {{{\rm{GH}}}}{{{{\rm{I}}}}}_{{{{\rm{ARI}}}}}=\left({{{\rm{GH}}}}{{{{\rm{I}}}}}_{{{{\rm{CTRL}}}}}^{{{{\rm{X}}}}}-{{{\rm{GH}}}}{{{{\rm{I}}}}}_{{{{\rm{ARIoff}}}}}^{{{{\rm{X}}}}}\right)-\left({{{\rm{GH}}}}{{{{\rm{I}}}}}_{{{{\rm{CTRL}}}}}^{{{{\rm{BS}}}}}-{{{\rm{GH}}}}{{{{\rm{I}}}}}_{{{{\rm{ARIoff}}}}}^{{{{\rm{BS}}}}}\right)$$

which represents the enhancement of GHI attributable to ARI under scenario X relative to BS.

### Estimation of PV installed capacity in 2060

The installed capacity potential of each province in 2060 is from Zhang et al.^62^, which incorporates the scenario-specific assumptions of the solar energy ratio. The data from Zhang et al.^63^ represent the technical installed capacity potential for each province. This potential is determined by considering wind and solar resource availability, as well as land use constraints, thus serving as a physical upper bound for PV deployment. As such, this technical potential is applied consistently across all our distinct carbon neutrality pathways (HB, CS, and HR) in this study. The projected PV capacity in 2060 under four pathways is provided in Table 1. The results are aggregated across China’s seven regional power grids^28^, i.e., Eastern, Central, Southern, Northeastern, Northern, Northwestern, and Xizang (map of power grids shown in Supplementary Fig. 5). Provinces in the northwest, such as Xizang, Xinjiang, Qinghai, Gansu, and Inner Mongolia, are characterized by abundant wind and solar resources and relatively low electricity demand, which could lead to penetration rates exceeding 80%. Conversely, coastal and central provinces, despite having poorer wind and solar resources, face high electricity demand, resulting in penetration rates potentially below 50%. This disparity vividly illustrates the spatial imbalance between China’s renewable energy supply and its electricity demand.

### Estimation of PV power-generation gains from improved air quality

In this study, we define the gains in PV power generation from improved air quality under carbon neutrality pathways, as expressed in Eq. (9).

9 $$	\Delta {{{\rm{P}}}}{{{{\rm{V}}}}}_{{{{\rm{POT}}}}({{{\rm{X}}}}-{{{\rm{BS}}}})}\cdot {{{{\rm{Capacity}}}}}_{{{{\rm{X}}}}}{=\Delta {{{\rm{P}}}}{{{{\rm{V}}}}}_{{{{\rm{POT}}}}}}_{\left({{{\rm{X}}}}-{{{\rm{BS}}}}\right)}\cdot {{{{\rm{Capacity}}}}}_{{{{\rm{BS}}}}} \\ 	+\Delta {{{\rm{P}}}}{{{{\rm{V}}}}}_{{{{\rm{POT}}}}\left({{{\rm{X}}}}-{{{\rm{BS}}}}\right)}\cdot {\Delta {{{\rm{Capacity}}}}}_{\left({{{\rm{X}}}}-{{{\rm{BS}}}}\right)}$$

where ${\Delta {{{\rm{P}}}}{{{{\rm{V}}}}}_{{{{\rm{POT}}}}}}_{({{{\rm{X}}}}-{{{\rm{BS}}}})}$ represents the change in PV_POT_ between carbon neutrality pathway X (HB, CS, or HR) and the BS due to reduced aerosol loading, and ${{{{\rm{Capacity}}}}}_{{{{\rm{X}}}}}$ denotes the installed PV capacity under carbon neutrality pathway X. This term therefore represents the PV generation gains attributable to improved air quality under the PV deployment scale of each carbon neutrality pathway. This total gain consists of two components: a pure resource (air-quality) effect, ${\Delta {{{\rm{P}}}}{{{{\rm{V}}}}}_{{{{\rm{POT}}}}}}_{({{{\rm{X}}}}-{{{\rm{BS}}}})}\cdot {{{{\rm{Capacity}}}}}_{{{{\rm{BS}}}}}$, representing the increase in PV generation due solely to improved PV_POT_ under the baseline installed capacity; and a synergistic effect, $\Delta {{{\rm{P}}}}{{{{\rm{V}}}}}_{{{{\rm{POT}}}}({{{\rm{X}}}}-{{{\rm{BS}}}})}\cdot {\Delta {{{\rm{Capacity}}}}}_{({{{\rm{X}}}}-{{{\rm{BS}}}})}$, which reflects the additional gains from improved air quality realized under expanded PV deployment in the carbon neutrality pathways.

Furthermore, we apply this methodology to each of the seven power grid regions in China. PV power generation is estimated as the product of PV_POT_ and installed capacity. Grid integration constraints such as curtailment and co-located storage deployment are not explicitly represented. By calculating the power generation increase for each region under each carbon neutrality pathway, we can identify the spatial heterogeneity in the benefits of PV power generation associated with air quality improvements and provide insights into the regional prioritization of renewable energy deployment and emission reduction efforts. Annual PV power generation is calculated as the product of the annual mean PV_POT_, the installed PV capacity, and the total number of hours in a year (8760 h). The annual mean PV_POT_ is derived from hourly simulations using the pvlib model driven by meteorological variables and represents the capacity factor of the PV system. The increase in PV generation under different scenarios is then multiplied by the assumed electricity price to estimate the corresponding economic benefits of PV power generation under different scenarios.

### Uncertainty analysis

To quantify the uncertainty associated with projected PV_POT_, PV power generation, and economic benefits increase under different carbon neutrality pathways, we focus on quantifying the uncertainty associated with GHI, which is regarded as one of the most critical sources of uncertainty in PV energy estimates^26^. While uncertainties may arise from multiple components of the integrated modeling framework—including energy–economy scenarios, emission inventories, atmospheric chemistry–meteorology simulations, PV performance models, and economic valuation—a comprehensive quantification of all sources remains challenging. A detailed discussion of these uncertainty sources and their limitations is provided in Supplementary Note 4.

To quantify GHI uncertainty, we applied the Easy Uncertainty Quantification (EasyUQ) framework, which statistically post-processes deterministic WRF-Chem outputs to generate probabilistic GHI estimates based on model–observation pairs^63^. The implementation involves: (1) pairing simulated and observed GHI data from 132 stations in 2021 for model training, (2) applying the Pool-Adjacent-Violators algorithm to derive conditional distributions, and (3) generating probabilistic GHI forecasts in 2060. These probabilistic GHI distributions were then propagated through the PV model chain to estimate the resulting uncertainty in PV_POT_, PV power generation, and economic benefits. Assuming stochastic independence between scenario uncertainties, the uncertainty of the difference was estimated using Eq. (10).

10 $${\sigma }_{\Delta }=\sqrt{{\sigma }_{X}^{2}+{\sigma }_{{{{\rm{BS}}}}}^{2}}$$

This approach provides uncertainty ranges (±1${\sigma }_{\Delta }$) around the central estimates of PV gain changes under carbon neutrality scenarios X relative to BS. As a result, the reported ±1${\sigma }_{\Delta }$ ranges should be interpreted as conservative upper-bound estimates of uncertainty.

## Supplementary information

Supplementary Information

Transparent Peer Review file

## Data Availability

The processed datasets generated in this study, including scenario-induced changes in GHI, PV_POT_, and PV power generation, are available at https://github.com/shihrong/PV-China-2060.git. The datasets have been archived on Zenodo at https://doi.org/10.5281/zenodo.21990987. The WRF-Chem model used in this study is available at https://github.com/wrf-model/WRF/tree/v4.2.

## Code availability

All necessary source codes, scripts, and simulation configurations (including WRF-Chem namelists and Python data pipelines) required to execute the models and reproduce the findings of this study have been made publicly available in the GitHub repository (https://github.com/shihrong/PV-China-2060.git). The specific version of the code used in this paper has been archived on Zenodo at https://doi.org/10.5281/zenodo.21990987.
