# Supplementary material for: Carbon neutrality strategies modulate air quality effects on solar power generation
Source: Nat Commun. 2026 Sep 1;17:10423. doi: 10.1038/s41467-026-77344-z (PMC13631228; doi:10.1038/s41467-026-77344-z)
Supplement: Supplementary file 1 [file 41467_2026_77344_MOESM1_ESM.pdf]

Supplementary Information for

Carbon Neutrality Strategies Modulate Air Quality Effects on Solar Power Generation

Hongrong Shi[1, 2], Da Gao[3, 4], Chenfei Qu[5], Xiang'ao Xia[6*], Dazhi Yang[7], Da Zhang[5], Mengmeng Song[7], Disong Fu[1], Baozhu Ge[1], Hongbin Chen[6], Yun Zhu[8], Rong Wang[9], Junji Cao[1], Shuxiao Wang[3, 4], Bin Zhao[3, 4*]

[1] State Key Laboratory of Atmospheric Environment and Extreme Meteorology, Institute of Atmospheric Physics, Chinese Academy of Sciences, Beijing, China

[2] Key Laboratory of Energy Meteorology, China Meteorological Administration (CMA-EML), Beijing, China

[3] State Key Joint Laboratory of Environment Simulation and Pollution Control, School of Environment, Tsinghua University, Beijing, China

[4] State Environmental Protection Key Laboratory of Sources and Control of Air Pollution Complex, Beijing, China

[5] Institute of Energy, Environment, and Economy, Tsinghua University, Beijing, China

[6] Laboratory of Middle Atmosphere and Global Environment Observation, Institute of Atmospheric Physics, Chinese Academy of Sciences, Beijing, China

[7] School of Electrical Engineering and Automation, Harbin Institute of Technology, Harbin, China

[8] School of Environment and Energy, South China University of Technology, Guangzhou, China

[9] Shanghai Key Laboratory of Atmospheric Particle Pollution and Prevention (LAP³), Department of Environmental Science and Engineering, Fudan University, Shanghai, China.

*Correspondence to: Xiang'ao Xia (xxa@mail.iap.ac.cn) and Bin Zhao (bzhao@mail.tsinghua.edu.cn)

Supplementary Information Contents

Supplementary Note 1……………………………………………………………………….1
Supplementary Note 2……………………………………………………………………….1
Supplementary Note 3……………………………………………………………………….2
Supplementary Note 4……………………………………………………………………….2
Supplementary Table 1……………………………………………………………………….3
Supplementary Table 2……………………………………………………………………….3
Supplementary Table 3……………………………………………………………………….4
Supplementary Figure 1……………………………………………………………………..5
Supplementary Figure 2……………………………………………………………………..6
Supplementary Figure 3……………………………………………………………………..7
Supplementary Figure 4……………………………………………………………………..7
Supplementary Figure 5……………………………………………………………………..8
Supplementary Figure 6……………………………………………………………………..9
Supplementary Figure 7…………………………………………………………………… 10
Supplementary Figure 8…………………………………………………………………… 11
Supplementary Figure 9…………………………………………………………………… 11
Supplementary References………………………………………………………………… 12

Supplementary Note 1. Field significance with false discovery rate control

To assess the robustness of spatial patterns and account for multiple testing across grid cells, we evaluated statistical significance using false discovery rate (FDR) control. Although a large number of grid cells satisfy nominal significance ($p < 0.05$) in all scenarios, the number of FDR-significant cells is substantially reduced, indicating limited spatial coherence at the grid scale.

For global horizontal irradiance (GHI), 4,273 and 4,769 grid cells satisfy $p < 0.05$ under the high biomass (HB) and carbon capture and storage (CS) scenarios, respectively, yet none (HB) or only two (CS) remain significant after FDR correction. In contrast, under the high renewable (HR) scenario, 189 grid cells remain FDR-significant, suggesting more spatially consistent changes in irradiance. A similar but more pronounced pattern is found for photovoltaic power potential (PV_{POT}). While more than 5,000 grid cells satisfy $p < 0.05$ under each scenario, only one to two grid cells remain FDR-significant under HB and CS, whereas 1,058 grid cells remain significant under the HR scenario. The concentration of FDR-significant PV_{POT} changes over eastern and southern China supports the robustness of the regional-mean enhancement identified in the main text. Although many grid cells satisfy nominal significance ($p < 0.05$), FDR control substantially reduces the number of significant cells, highlighting the importance of accounting for multiple testing in spatial analyses.

Supplementary Note 2. Robustness of projected global horizontal irradiance and photovoltaic power potential enhancements to climate forcing and interannual variability

To evaluate the robustness of the projected air-quality-induced enhancements in global horizontal irradiance (GHI), and photovoltaic power potential (PV_{POT}), we conducted two complementary sensitivity analyses. First, to assess the influence of climate forcing uncertainty, an additional set of simulations was performed using meteorological boundary conditions derived from the Meteorological Research Institute Earth System Model version 2.0 (MRI-ESM2-0) model [4], while the main simulations employed the bias-corrected ensemble mean of 18 Coupled Model Intercomparison Project Phase 6 (CMIP6) models. Second, to examine the impact of interannual variability, we conducted additional simulations for 2058 under both the high renewable (HR) and baseline (BS)scenarios and compared the results with the original 2059–2061 simulations.

The resulting spatial distributions of ΔGHI and ΔPV_{POT} are highly consistent with those obtained using the CMIP6 ensemble-mean forcing (Supplementary Figure 6). Quantitatively, the regional mean responses across China's seven power-grid regions are strongly correlated between the two simulations, yielding a coefficient of determination (R^2) of 0.79 for both variables and preserving identical regional rankings (Supplementary Figure 7). This suggests that the ensemble-mean forcing adopted in the main analysis provides a representative estimate of future meteorological conditions.

The resulting spatial distributions of ΔGHI and ΔPV_{POT} in 2058 closely resemble those obtained from the 2059–2061 mean simulations (Supplementary Figure 8). Quantitatively, the regional mean responses across China's seven power-grid regions are strongly correlated between the two periods, with R^2 values of 0.96 for ΔGHI and 0.96 for $\Delta PV_{POT,}$ and with the regional ranking of projected benefits remaining unchanged (Supplementary Figure 9). The consistency in both spatial patterns and regional responses demonstrate that the projected air-quality-induced enhancements are robust to interannual meteorological variability.

Taken together, these sensitivity analyses demonstrate that the projected air-quality-induced

improvements in GHI and photovoltaic potential are robust to both climate forcing uncertainty and interannual variability, supporting the reliability of the conclusions presented in the main manuscript.

Supplementary Note 3. The simulated global horizontal irradiance evaluation results

Supplementary Table 3 presents the evaluation of simulated daily global horizontal irradiance (GHI) against ground-based observations for 2013 and 2021. Across the seven regional power grids, The Weather Research and Forecasting model coupled with Chemistry (WRF-Chem) reproduces the observed spatial variability of GHI reasonably well. Nationally averaged relative mean bias error (rMBE) values are 12.4% and 10.4% for 2013 and 2021, respectively, while corresponding relative root mean square error (rRMSE) values are 33.6% and 32.2%. The similar performance across the two years suggests that the model provides a stable representation of surface solar radiation under substantially different aerosol pollution conditions.

Supplementary Note 4. Sources of uncertainty and limitations

To quantify the uncertainty associated with projected photovoltaic power potential (PV_{POT}), photovoltaic (PV) power generation and economic gains under different carbon-neutrality pathways relative to the baseline, uncertainties across the entire integrated modeling framework should ideally be considered, including energy–economy scenarios, emission inventories, atmospheric chemistry–meteorology simulations, PV performance models, and economic valuation. In principle, a fully integrated Monte Carlo or multi-model ensemble propagating uncertainties across all components would provide a comprehensive assessment; however, such an end-to-end uncertainty propagation is computationally prohibitive in practice. In this study, we therefore focus on explicitly quantifying the uncertainty associated with simulated solar radiation (GHI) from the Weather Research and Forecasting model coupled with Chemistry (WRF-Chem), which represents the dominant driver of variability in PV performance [1]. These uncertainties are quantified using the Easy Uncertainty Quantification (EasyUQ) framework and propagated through the pvlib-based PV modeling chain to estimate the resulting variability in PV_{POT}, PV power generation and economic benefits increase.

Additional sources of uncertainty may arise from emission trajectories derived from the China-in-Global Energy Model (C-GEM) energy–economy model, assumptions about future technology deployment and emission factors, scheme uncertainties in atmospheric chemistry–meteorology simulations, parameter assumptions in the pvlib-based PV performance modeling, and the electricity price used in the economic valuation. While these factors may influence the absolute magnitude of projected PV benefits, the primary objective of this study is to compare relative differences among carbon neutrality pathways. Because the same modeling framework and parameter assumptions are consistently applied across scenarios, many systematic biases are expected to be minimized when evaluating pathway differences. Furthermore, the integrated framework developed here is transferable to other regions, provided that region-specific emission scenarios, meteorological projections, and PV deployment pathways are available.

Despite the robust nationwide gains identified for scenario contrasts, we acknowledge certain limitations in our uncertainty characterization. While our national-level assessment provides a clear signal for macro-scale energy policy, it may mask regional heterogeneities in model performance. Future research employing spatially-clustered bootstrapping or multi-model ensembles could further refine these local-scale uncertainties and provide more granular insights for regional grid planning.

Supplementary Table 1. Key characteristics of the three carbon-neutrality pathways in China in 2060. The three pathways are the high-biomass (HB), carbon-storage (CS) and high-renewable (HR) pathways.

Carbon Neutrality pathways*	HB	CS	HR
Total CO_2 captured in biomass burning per year ($GtCO_2$ $year^{-1}$)	0.8	0.5	0.2
Total CO_2 captured by direct air capture technology per year ($GtCO_2$ $year^{-1}$)	0.2	0.4	0.1
Total CO_2 captured in coal and gas electricity generation and industry ($GtCO_2$ $year^{-1}$)	1.2	1.3	0
Total CO_2 captured per year ($GtCO_2$ $year^{-1}$)	2.2	2.2	0.3
Share of non-fossil energy in primary energy consumption (%)	80	80	97
Wind and solar generations (PWh)	11	12	15
Electrification rate, i.e., share of electricity in final energy consumption (%)	65	67	95

*Data from Gao, et al. [3]

Supplementary Table 2. Configuration of the Weather Research and Forecasting model coupled with Chemistry used in this study. The table summarizes the principal model domains, spatial resolution, physical parameterizations, chemical mechanisms and emission settings used in the Weather Research and Forecasting model coupled with Chemistry (WRF-Chem) simulations.

Configuration	Description
Horizontal Resolution	27 km × 27 km
Grid Dimensions	182 (West-East) × 232 (South-North)
Vertical Layers	24 layers (top at 50 hPa)
Radiation Scheme	RRTMG (Shortwave and Longwave)
Microphysics	Morrison 2-moment scheme
Surface layer scheme	Eta similarity
Cumulus Scheme	Grell-3
Planetary boundary layer scheme	Bougeault–Lacarrère
Land Surface	Noah Land Surface Model
Biogenic emissions	MEGAN v2.04
Dust emissions	GOCART
Photolysis rates	Fast-J photolysis scheme
Gas-phase Mechanism	CBMZ
Aerosol Module	MOSAIC (8-bin)
Secondary organic aerosol	volatility basis set (VBS) framework

Supplementary Table 3. Performance statistics for simulated daily global horizontal irradiance across China's seven regional power grids in 2013 and 2021. Model-simulated daily global horizontal irradiance (GHI) was evaluated against observational data. The seven power-grid regions are Eastern, Central, Southern, Northeastern, Northern, Northwestern and Xizang. Mean bias error (MBE), relative mean bias error (rMBE), root mean square error (RMSE) and relative root mean square error (rRMSE) are reported. "National" denotes statistics calculated using all observational sites and corresponding model grid cells across China.

Power Grid Region	Year	Mean observation (W m^{-2})	Mean simulation (W m^{-2})	MBE (W m^{-2})	rMBE (%)	RMSE (W m^{-2})	rRMSE (%)
Eastern	2013	174.2	197.3	18.3	10.5%	43.7	25.1%
	2021	155.4	169.1	15.9	10.2%	53.3	34.3%
Central	2013	156.7	172.6	13.7	8.8%	61.6	39.4%
	2021	149.3	166.2	17.2	11.5%	58.9	39.4%
Southern	2013	135.0	167.5	31.6	23.3%	66.7	49.5%
	2021	164.8	181.5	17.6	10.7%	67.2	40.7%
Northeastern	2013	145.0	159.5	13.3	9.1%	41.9	28.9%
	2021	154.7	171.6	13.3	8.6%	44.7	28.9%
Northern	2013	170.2	192.3	21.7	12.7%	45.6	26.8%
	2021	168.5	186.3	17.3	10.2%	46.6	27.7%
Northwestern	2013	174.4	206.9	29.6	16.9%	51.0	29.2%
	2021	188.0	206.5	18.7	9.9%	49.3	26.2%
Xizang	2013	234.2	235.2	-0.1	-0.1%	57.1	24.3%
	2021	222.0	245.8	23.7	10.6%	69.8	31.4%
National	2013	162.4	183.9	20.1	12.4%	54.6	33.6%
	2021	170.0	187.3	17.6	10.3%	54.8	32.2%

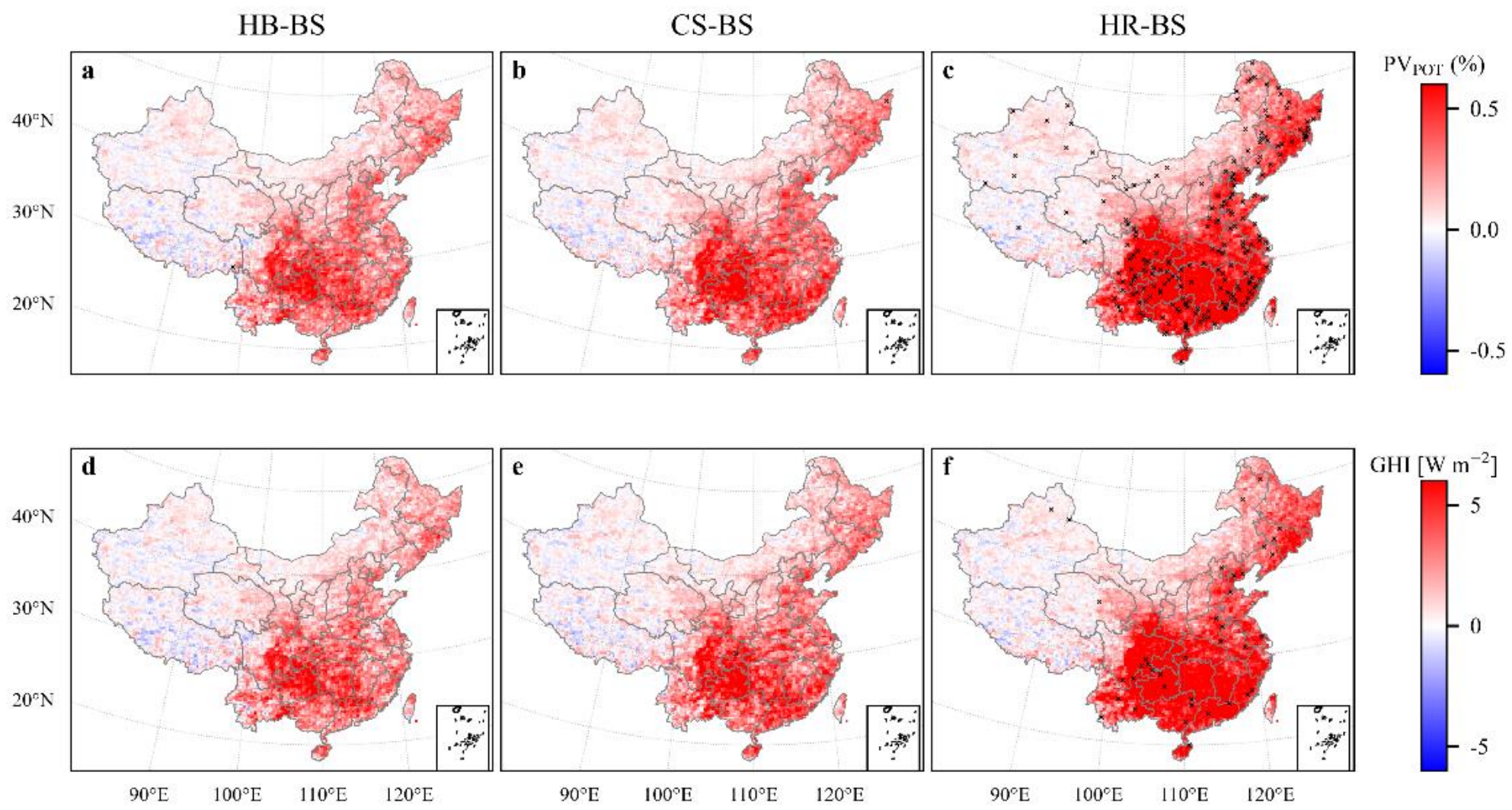

Supplementary Figure 1. Statistical significance of changes in photovoltaic power potential and global horizontal irradiance under different carbon-neutrality pathways. a–c, Spatial distributions of changes in photovoltaic power potential (PV_{POT}) under the high-biomass (HB), carbon-storage (CS) and high-renewable (HR) pathways, respectively, relative to the baseline scenario (BS) in 2060. **d–f,** Corresponding changes in global horizontal irradiance (GHI) under HB, CS and HR, respectively. Colour shading denotes grid-cell-level differences. Black dots indicate grid cells that remain statistically significant after controlling the false discovery rate (FDR) at $q = 0.05$ using the Benjamini–Hochberg procedure. Statistical significance was evaluated using paired *t*-tests applied to annual means from the three-year simulations covering 2059–2061. Only grid cells satisfying the FDR criterion are marked.

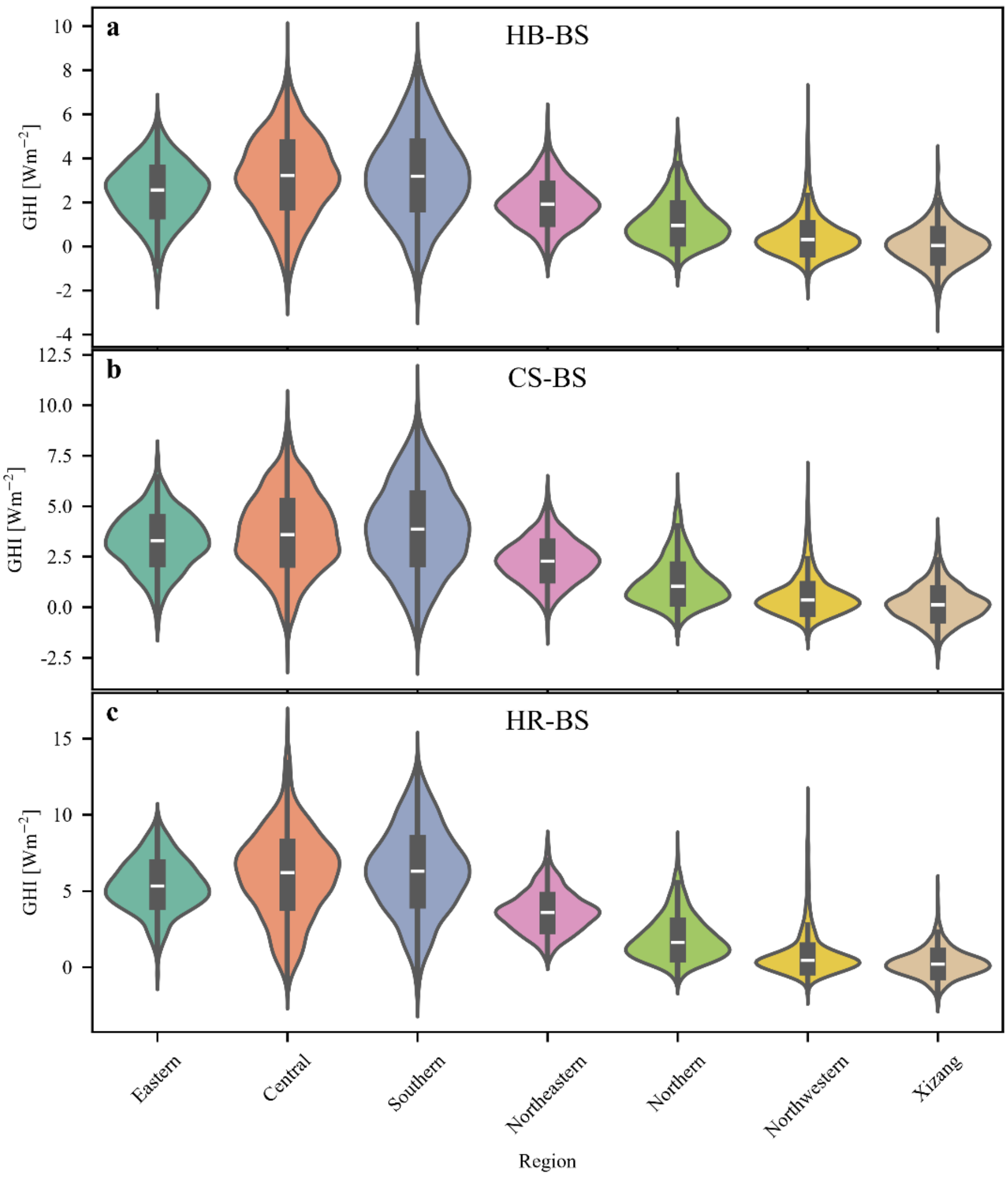

Supplementary Figure 2. Regional distributions of simulated changes in global horizontal irradiance. a–c, Changes in global horizontal irradiance (GHI) under the high-biomass (HB), carbon-storage (CS) and high-renewable (HR) pathways, respectively, relative to the baseline scenario (BS) in 2060. Results are shown for China's seven regional power grids: Eastern, Central, Southern, Northeastern, Northern, Northwestern and Xizang. The width of each violin represents the density of values. Positive and negative values indicate increases and decreases relative to BS, respectively. The distributions indicate pronounced regional variability, with the largest increases and broadest distributions generally occurring in the Eastern, Central and Southern regions, whereas changes in the Northwestern and Xizang regions are smaller and more narrowly distributed. The magnitude of the GHI increase generally progresses from HB to CS and HR.

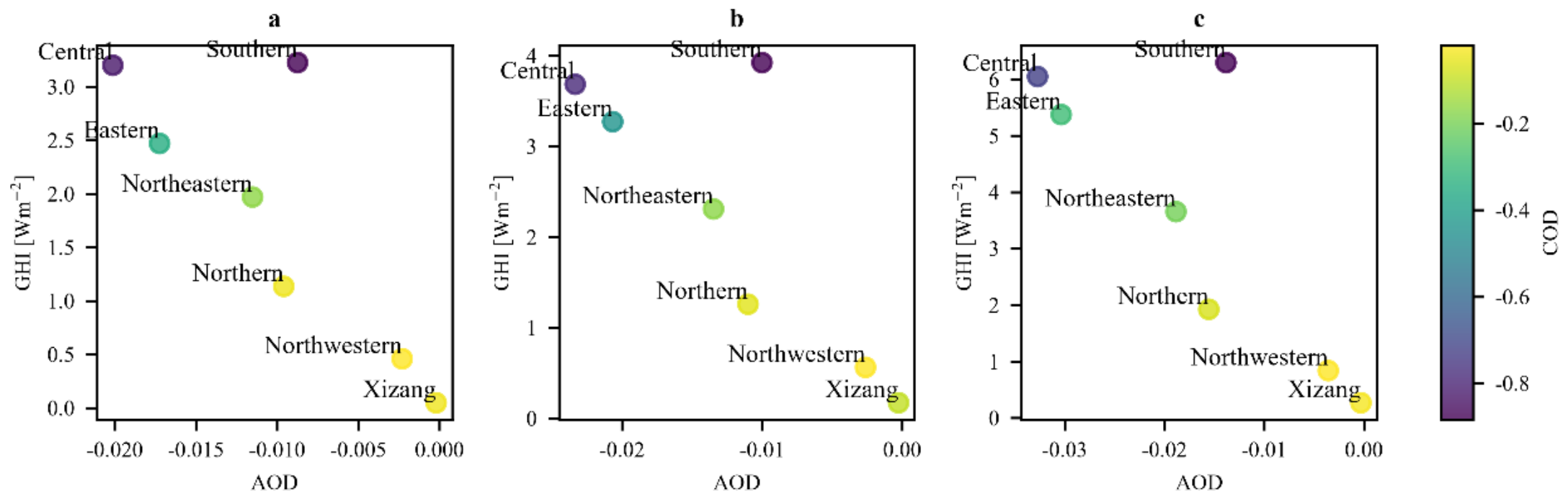

Supplementary Figure 3. Relationships among changes in aerosols, clouds and surface solar radiation under different carbon-neutrality pathways. a–c, Relationships between changes in aerosol optical depth (AOD; x axis) and global horizontal irradiance (GHI; y axis) under the high-biomass (HB; a), carbon-storage (CS; b) and high-renewable (HR; c) pathways relative to the baseline scenario (BS) in 2060. Point colour indicates the corresponding change in cloud optical depth (COD). Differences are calculated as the pathway value minus the BS value. Each point represents the mean value for one of China's seven regional power grids. AOD and COD are dimensionless, and GHI is expressed in W m^{-2}.

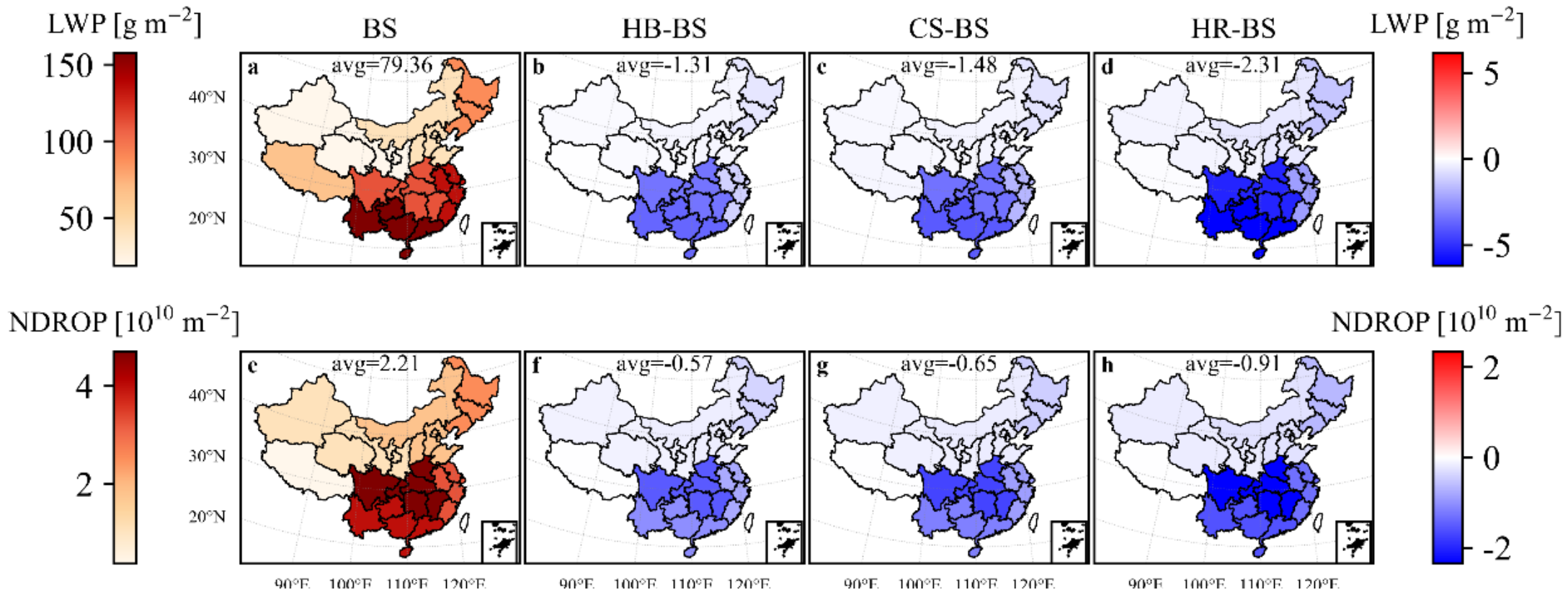

Supplementary Figure 4. Spatial distributions of liquid water path and integrated cloud droplet number concentration under different carbon-neutrality pathways. a–d, liquid water path (LWP), and **e–h,** integrated cloud droplet number concentration (NDROP) across China in 2060. Within each row, the leftmost panel shows the absolute value under the baseline scenario (BS), whereas the remaining panels show changes under the high-biomass (HB), carbon-storage (CS) and high-renewable (HR) pathways relative to BS, denoted as HB–BS, CS–BS and HR–BS, respectively.

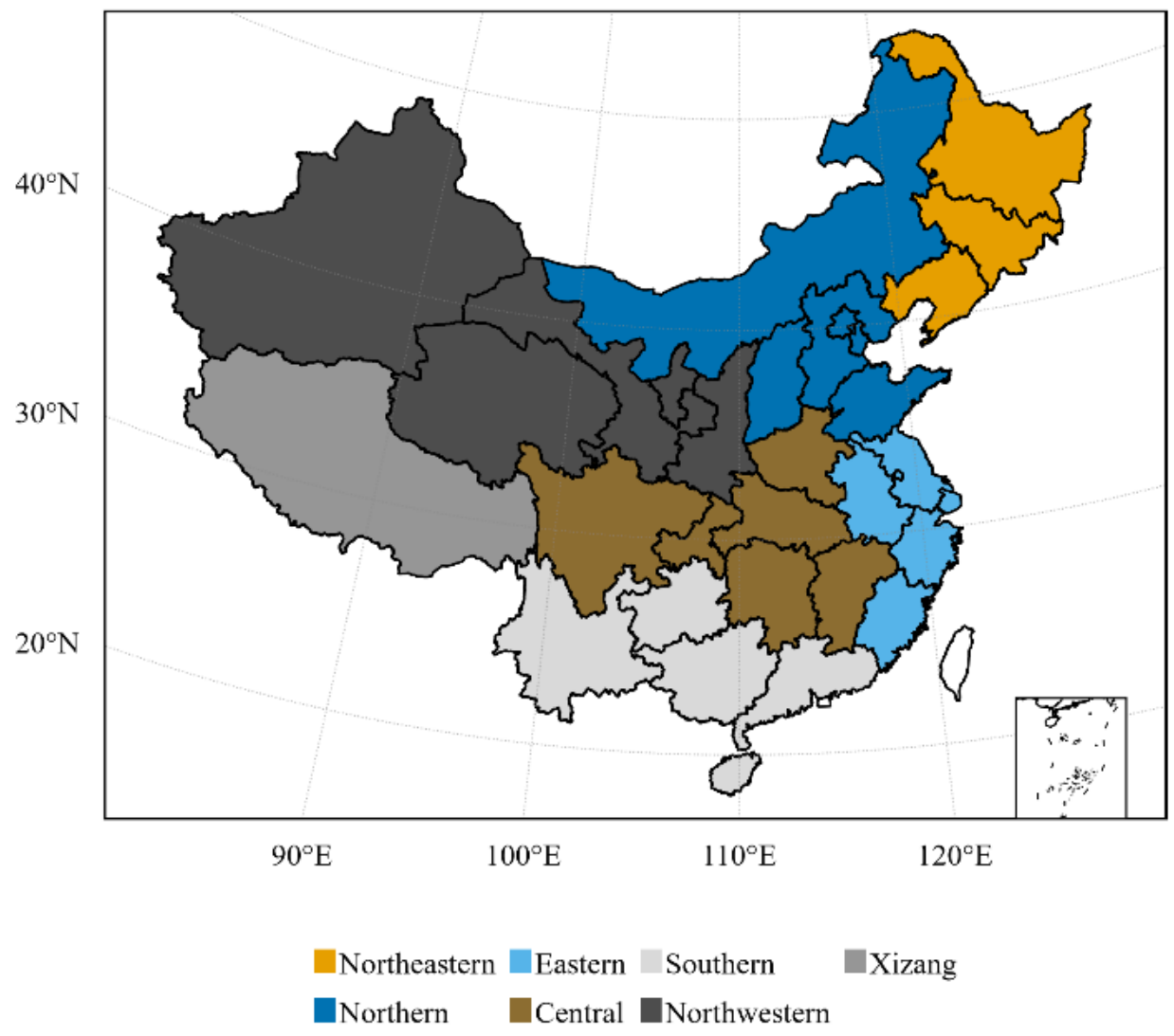

Supplementary Figure 5. China's inter-provincial regional power grids. Colours distinguish the seven power-grid regions used in this study: Eastern, comprising Shanghai, Jiangsu, Zhejiang, Anhui and Fujian; Central, comprising Henan, Hubei, Hunan, Jiangxi, Chongqing and Sichuan; Southern, comprising Guangdong, Guangxi, Hainan, Guizhou and Yunnan; Northeastern, comprising Liaoning, Jilin and Heilongjiang; Northern, comprising Beijing, Tianjin, Hebei, Shanxi, Inner Mongolia and Shandong; Northwestern, comprising Shaanxi, Gansu, Ningxia, Qinghai and Xinjiang; and Xizang. These regional divisions are used throughout the study to aggregate and compare changes in photovoltaic power potential (PV_{POT}) and photovoltaic (PV) power generation.

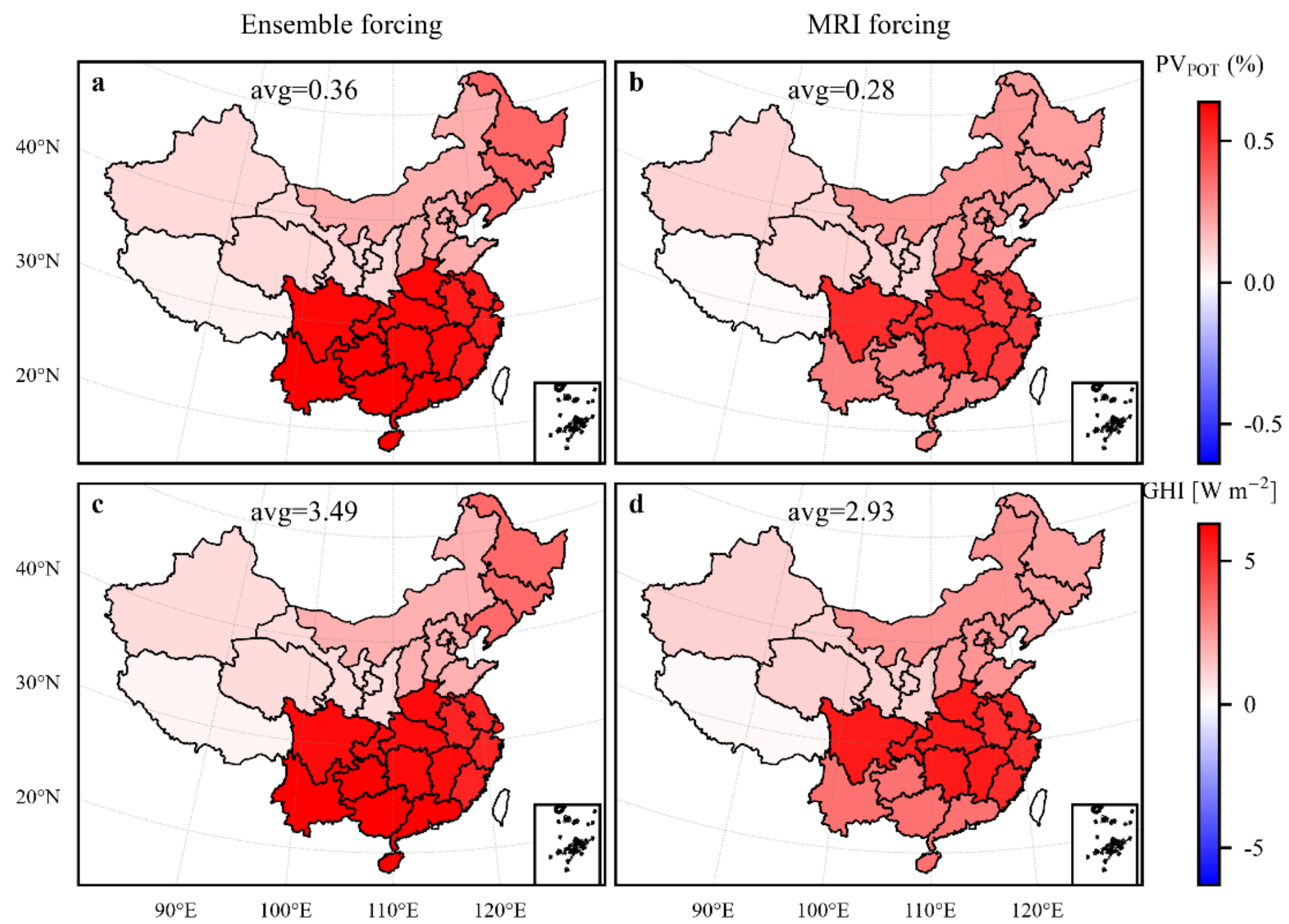

Supplementary Figure 6. Sensitivity of projected solar-resource changes to the meteorological forcing dataset. a,b, Spatial distributions of changes in global horizontal irradiance (GHI), and **c,d,** changes in photovoltaic power potential (PV_{POT}), under the high-renewable (HR) pathway relative to the baseline scenario (BS) in 2060. "Ensemble forcing" denotes the ensemble-mean meteorological forcing derived from 18 models participating in Phase 6 of the Coupled Model Intercomparison Project (CMIP6), whereas "MRI forcing" denotes the independent forcing derived from the Meteorological Research Institute Earth System Model version 2.0 (MRI-ESM2-0). Panels a and c show results obtained using "Ensemble forcing", whereas panels b and d show results obtained using "MRI forcing". Despite differences in the magnitudes of local responses, the two forcing datasets produce similar spatial patterns, with the largest increases concentrated in eastern and central China.

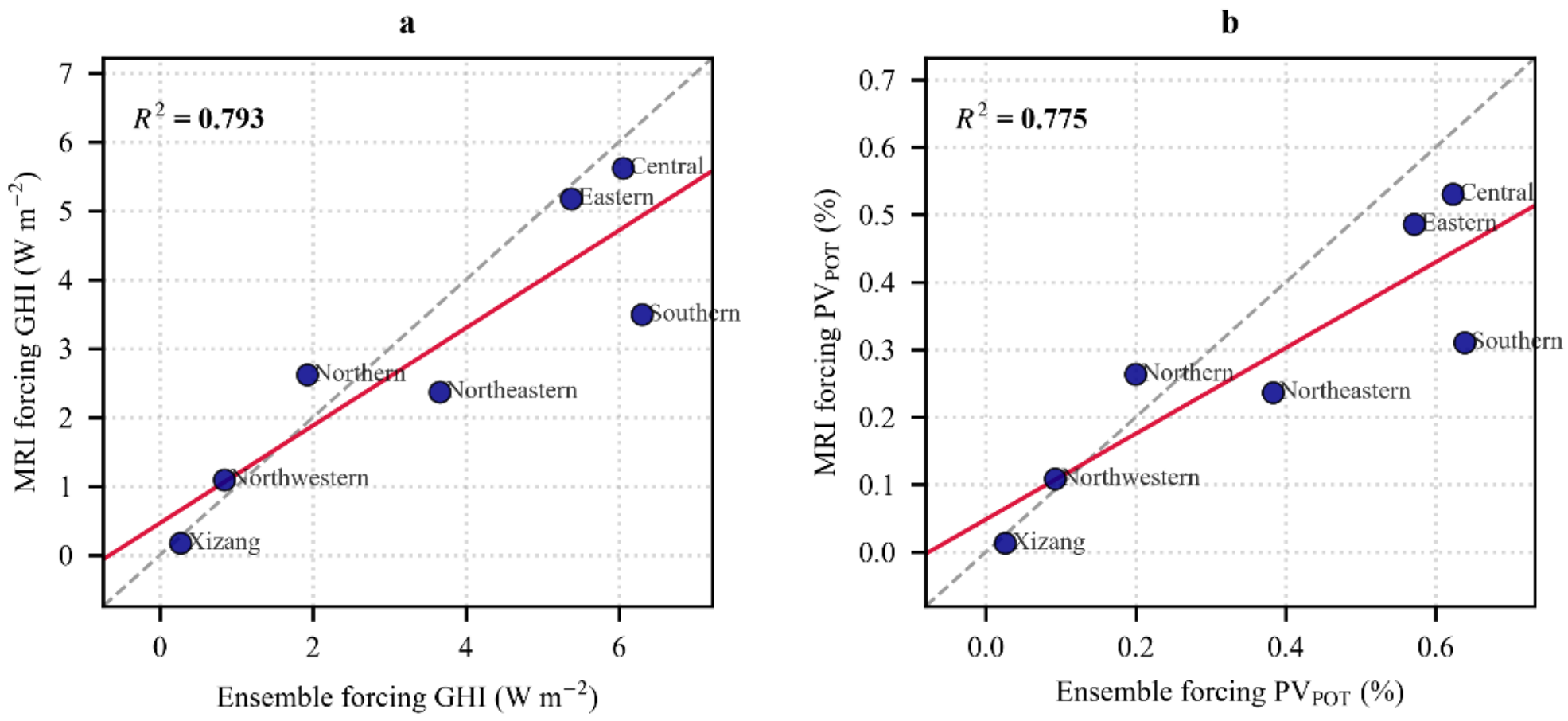

Supplementary Figure 7. Comparison of regional solar-resource changes simulated using two meteorological forcing datasets. a, Comparison of regional mean changes in global horizontal irradiance (GHI), and **b,** comparison of regional mean changes in photovoltaic power potential (PV_{POT}), under the high-renewable (HR) pathway relative to the baseline scenario (BS) in 2060. The horizontal and vertical axes show results obtained using the ensemble forcing from 18 models participating in Phase 6 of the Coupled Model Intercomparison Project (CMIP6) and the independent Meteorological Research Institute Earth System Model version 2.0 (MRI-ESM2-0) forcing, respectively. Each point represents one of China's seven regional power grids. The coefficient of determination is $R^2 = 0.79$ for both changes in GHI and PV_{POT}, indicating that the projected benefits for solar resources and photovoltaic performance are relatively insensitive to the choice of meteorological forcing dataset.

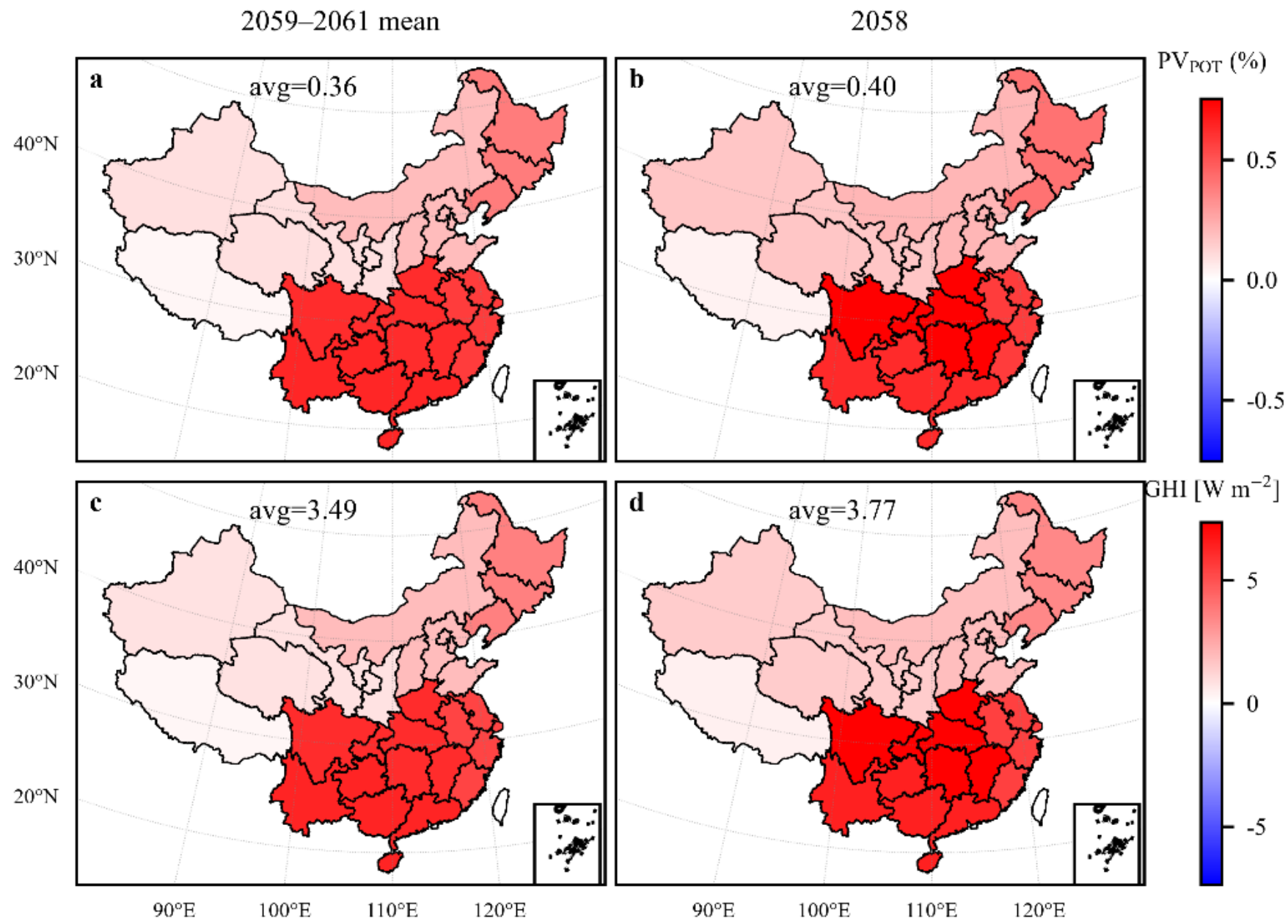

Supplementary Figure 8. Sensitivity of projected solar-resource changes to interannual variability. a,b, Spatial distributions of changes in global horizontal irradiance (GHI), and **c,d,** changes in photovoltaic power potential (PV_{POT}), under the high-renewable (HR) pathway relative to the baseline scenario (BS). Panels **a** and **c** show the means of the simulations for 2059–2061, whereas panels **b** and **d** show results for the independent year 2058. The similar spatial patterns obtained for the two periods indicate limited sensitivity of the projected changes to interannual variability.

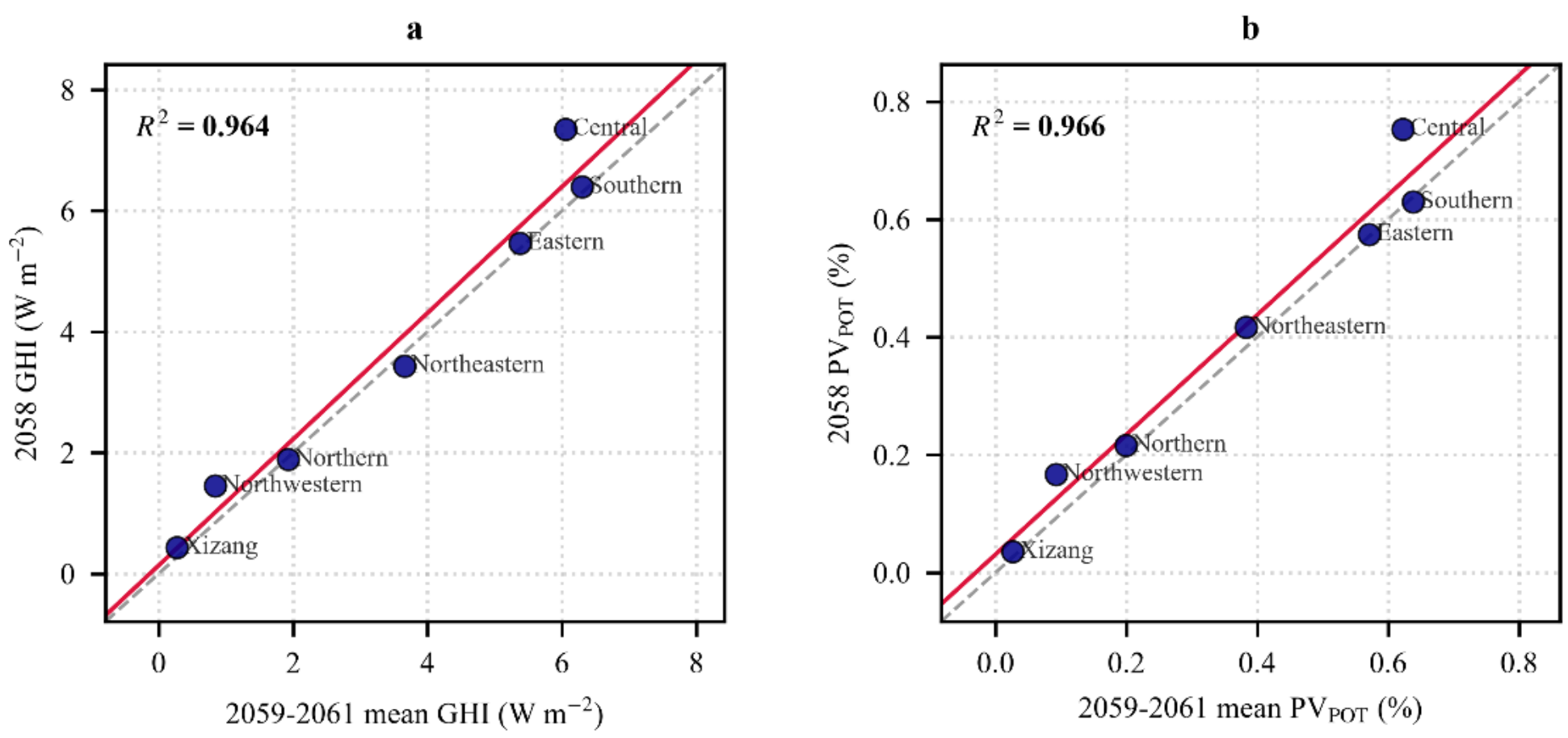

Supplementary Figure 9. Comparison of regional solar-resource changes between an independent year and the three-year simulation mean. a, Comparison of regional mean changes in global horizontal irradiance (GHI), and **b,** comparison of regional mean changes in photovoltaic power potential (PV_{POT}), under the high-renewable (HR) pathway relative to the baseline scenario (BS). The horizontal and vertical axes show results for the 2059–2061 mean and the independent year 2058, respectively. Each point represents one of China's seven regional power grids. The high coefficients of determination ($R^2 > 0.9$) between the two periods indicate that the projected enhancements in solar resources and photovoltaic performance are robust to interannual variability.

Supplementary References

1. Prilliman, M. J. et al. Quantifying uncertainty in PV energy estimates: Final report. National Renewable Energy Laboratory, Golden, CO (2023).
2. Zhuo, Z. et al. Cost increase in the electricity supply to achieve carbon neutrality in China. *Nat. Commun.* **13**, 3172 (2022).
3. Gao, D. et al. Different carbon neutrality strategies induce substantially divergent health benefits and distributional impacts in China. *Environ. Sci. Technol.* **59**, 27517–27529 (2025).
4. Kawai, H. et al. Significant improvement of cloud representation in the global climate model MRI-ESM2. *Geosci. Model Dev.* **12**, 2875–2897 (2019).
