# Supplementary material for: Carbon neutrality strategies modulate air quality effects on solar power generation
Source: Nat Commun. 2026 Sep 1;17:10423. doi: 10.1038/s41467-026-77344-z (PMC13631228; doi:10.1038/s41467-026-77344-z)
Supplement: Supplementary file 2 [file 41467_2026_77344_MOESM2_ESM.pdf]

nature portfolio

Peer Review File

Carbon Neutrality Strategies Modulate Air Quality Effects on Solar Power Generation

Corresponding Author: Dr Xiangao Xia

This file contains all reviewer reports in order by version, followed by all author rebuttals in order by version.

Attachments originally included by the reviewers as part of their assessment can be found at the end of this file.

Version 0:

Reviewer comments:

Reviewer #1

(Remarks to the Author)
I enjoyed the opportunity to read manuscript "NCOMMS-25-58082 - Carbon Neutrality Strategies Modulate Air Quality Effects on Solar Power Generation." The paper builds an integrated chain—C-GEM (policy/energy–economy) → WRF-Chem (air quality & meteorology) → pvlib PV model—to claim that China's "High Renewables" pathway in 2060 delivers ~9,850±1,766 GWh/yr extra PV generation (≈$0.89±0.16 bn/yr) relative to a baseline, largely via ACI rather than ARI. It further asserts these air-quality gains exceed material-driven PV efficiency improvements and are concentrated in eastern/central China. The topic is important, but the manuscript overreaches on identification, attribution, valuation, and uncertainty. As written, the policy-relevant economic conclusions are not credible.

Major Comments
1. The ACI vs ARI partition mainly infers "dominance" from GHI–GHI_c differences. That is not a causal ACI identification. You must run paired physics toggles (ARI-on/ACI-off; ACI-on/ARI-off) and microphysics/radiation scheme ensembles to isolate process contributions. Without them, "ACI dominates" is unsubstantiated and highly model-dependent.

2. Simulating only 2059–2061 cannot robustly represent the distribution of cloud/aerosol states or PV resources. At minimum, use decadal slices (e.g., 2051–2060) and/or multi-member perturbed-initial-condition ensembles to quantify sampling uncertainty.

3. Future chemical initial/boundary conditions are proportionally reduced from today and tied to an SSP126 ensemble mean. This shortcut risks dynamical inconsistency between meteorology and composition. You need multi-GCM, multi-SSP chemistry–meteorology boundary experiments (or fully consistent CTM boundaries) with clear propagation of inter-model spread.

4. The claim that biases "cancel in differences" is an unsupported assumption. Perform site-wise bias correction (e.g., quantile mapping) with cross-validation, then re-run the PV chain; report how corrected vs uncorrected GHI alter PVPOT deltas and money metrics. Also quantify spatially structured error—differences do not guarantee cancellation.

5. The "GHI contributes ~100%" result is constructed by one-at-a-time substitutions holding other variables at baseline. Because PV output is nonlinear in (GHI, T, WS) and these fields are correlated, this approach misattributes interactions. Use Shapley value decomposition / ANOVA over factorial combinations to get a path-independent attribution with uncertainty.

6. The two-tier downscaling lacks inventory validation at sub-provincial scales and ignores plant siting and sector-specific controls. Provide ex-post checks against observed spatial AOD/$PM_{2.5}$ patterns and sectoral proxies; otherwise, PV benefits in high-demand east/central regions could be artifactually inflated.

7. Equation (3) labels both ΔPVPOT·Capacity_BS and ΔPVPOT·ΔCapacity as "aerosol-induced." The second term depends on capacity expansion, which is driven by policy/tech economics—not aerosols per se. Treat pure resource effect and capacity siting/scale effect separately; else you over-attribute economic gains to air quality.

8. Using a flat $0.09/kWh in 2060, no curtailment, no congestion, no LMP/capacity value differentiation, and no system integration (storage, transmission) overstates benefits. Replace with power-system–consistent valuation (e.g., marginal value of PV energy by region/hour from a capacity-expansion/UC-ED model) and apply discounting and price scenarios.

9. EasyUQ treats GHI only. Missing: C-GEM tech shares, emission factors, wrf-chem physics choices, ACI parameterization, pvlib model choices, price paths, capacity trajectories. Provide a full Monte Carlo / multi-model ensemble and tornado plots of elasticities to key assumptions.

10. This paper uses the north-easterly winds to estimate the impact of Korean air pollution on Chinese air quality and it would be recommended using all of the Easterly winds (Northeast, East, Southeast) to examine the changes in air pollution levels from Beijing to Shanghai. In the case of Southeast wind, how do the authors disentangle the spillover impacts of South Korea from Japan?

11. You cite past "brightening" after 2013 policy, but do not validate the model chain against observed PV output (plant- or province-level) over 2015–2023 concurrent with measured PM reductions. A credible study should reproduce historical PV gains due to cleaned air before projecting 2060.

12. HR/HB/CS are described qualitatively. You need transparent tabulation of sectoral activity, emissions by precursor, and control measures by region to justify the differential aerosol response that drives PV benefits—and a sensitivity to alternative feasible mixes within each pathway.
13. Lower COD and higher GHI could arise from dynamical changes (e.g., storm tracks, monsoon onset) unrelated to aerosol microphysics. Provide diagnostics (omega, RH profiles, cloud regimes) and causal mediation analysis before crediting ACI.

14. PV value often hinges on peak-demand and cloudy/clear extremes. A 3-year slice misses tail risks. Use longer climatologies and report extreme-percentile impacts on PV (e.g., P5/P95 of GHI and PV value).

15. The statement that air-quality PV gains are "~4× material-driven efficiency progress" cherry-picks a 0.5%/yr heuristic and ignores system-level module and BOS developments (bifacial, trackers, albedo, wiring, inverters) and learning rates to 2060. Provide a range from credible tech roadmaps and compare system yield, not a single efficiency proxy.

16. There is no formal statistical design for causal attribution (only scenario contrasts). Where you report $\pm 1\sigma$, clarify what stochastic source it represents; then add block bootstrap over time and space to reflect field correlation, and clustered uncertainty by region.

17. Numerous maps and comparisons are made without FWER/FDR control. Report field significance and False Discovery Rate for spatial patterns.

18. Standard errors that ignore spatial autocorrelation are too small. Use Conley-type or random field corrections when aggregating to regional effects.

19. Hold out subsets of ground stations and assess predictive skill of GHI/PVPOT deltas before monetization.

Minor Comments
1. Distinguish absolute vs relative PVPOT changes (e.g., 0.36% absolute ≈ 2% relative to 20.2%).
2. Add joint AOD–COD–GHI scatter and regional violin plots with uncertainty bands; show difference-of-bias-corrected results.
3. Report curtailment assumptions and co-located storage shares by region.
4. "Available upon request" is inadequate. Provide public repos (C-GEM scenarios, downscalers, WRF-Chem namelists, pvlib pipeline) and replication scripts end-to-end.

(Remarks on code availability)
No codes are available for review

Reviewer #2

(Remarks to the Author)
Attached

(Remarks on code availability)
Looks okay.

Reviewer #3

(Remarks to the Author)
I co-reviewed this manuscript with one of the reviewers who provided the listed reports. This is part of the Nature Communications initiative to facilitate training in peer review and to provide appropriate recognition for Early Career Researchers who co-review manuscripts.

(Remarks on code availability)

Reviewer #4

(Remarks to the Author)
The paper presents very interesting results, showing for the first time a quantitative economic assessment of the impact of different carbon neutrality pathways on PV production in China. Unlike most previous works that evaluate solar potential, in this case, the climate and energy models are coupled in a modeling chain. The outputs of changes in energy production are then evaluated and, going a step further, these are expressed in economic terms, although through a relatively simple conversion. One of the interesting aspects is that the authors put the results into context to help understand the magnitude of the economic changes. This work allows for a comparison of the impacts of different carbon neutrality strategies, which is important given the dependence of photovoltaic production on air quality.

I found a weakness in the fact that only one climate model was used for the simulation of the meteorological variables, which limits the robustness of the results presented. Part of the uncertainty arises from the model itself; therefore, using an ensemble of models would enhance the reliability of the results. In addition, a three-year simulation period is too short to capture variability properly. Longer simulations are needed to obtain representative mean values. I assume the authors tried to address this limitation using the probabilistic approach explained at the end of the paper — namely, EasyUQ as a post-processing technique. However, the paper should be revised on this point. An evaluation of the accuracy of this statistical approach should be provided, and the limitations related to the uncertainty of using a single climate model and a short simulation period must be clearly stated in the text.

Another relevant point is that solar radiation (GHI) was evaluated against ground stations but only for one year. A longer validation period is required to assess the model's performance across different time scales.

In general, the paper presents very interesting approaches to the problem, such as considering both the direct and indirect effects of aerosols on solar radiation, as well as implementing a complete modeling chain from atmospheric variables to economic gains.

Some specific comments:

- The code should be made publicly available on GitHub or a similar platform to allow replication of the results.

- The expression "actual conditions" in the text is unclear; please specify what is meant (see for instance line 89 and elsewhere).

- PV modeling is performed using the pvlib package; however, the models used at each stage of the process are not clearly described — namely: decomposition into direct and diffuse components, and transposition into the plane-of-array.

- Although the approach in this paper is novel, there are previous studies that have evaluated the impact of aerosols and their future changes (natural or anthropogenic) on solar radiation and, consequently, on photovoltaic potential that has not been consider in the manuscript. See, for instance: Isaza et al. (2023) where an additional effort to see economic impact is being also performed. Boé et al. (2020), Gutiérrez et al. (2020) for the impact of aerosols evolution, and Jerez et al. (2021) for the indirect effect of aerosols.

(Remarks on code availability)
The code should be available in a public repository.

Version 1:

Reviewer comments:

Reviewer #1

(Remarks to the Author)
The authors have submitted a highly responsive and rigorous revision. The original manuscript presented an ambitious, integrated modeling chain to evaluate the economic and physical impacts of pathway-specific air quality improvements on solar PV generation in China. The reviewers rightly pointed out several methodological weaknesses in the original draft—specifically regarding causal attribution, nonlinear interactions, spatial statistics, and transparency.

The authors have done a commendable job addressing these criticisms. The revised manuscript is substantially stronger, more transparent, and methodologically sound. It is suitable for publication after a few minor technical checks.

General comments:
1. The transition from a one-at-a-time (OAT) substitution method to a Shapley value-based decomposition is a major improvement. Because PV potential responds nonlinearly to global horizontal irradiance (GHI), temperature (T2), and wind speed (WS), the Shapley framework provides a path-independent and mathematically robust attribution. This successfully addresses Reviewer 1's concerns regarding interaction effects.

2. In the previous version, claiming that aerosol-cloud interactions (ACI) dominated over aerosol-radiation interactions (ARI) based solely on clear-sky vs. all-sky diagnostics was a significant overreach. The authors resolved this by running paired physical sensitivity experiments (ACI-off/ARI-on and ARI-off/ACI-on). This process-level toggling explicitly isolates the radiative contributions and confirms the dominance of ACI (accounting for roughly three-quarters of the total radiation enhancement).

3. The inclusion of grid-cell-level paired t-tests with FDR control using the Benjamini-Hochberg procedure at $q=0.05$ adds necessary statistical rigor. Acknowledging that the High Biomass (HB) and Carbon Storage (CS) scenarios show limited spatial coherence at the grid scale, while the High Renewables (HR) scenario maintains significance, prevents the over-interpretation of spatial maps.

4. The addition of Table S1 (macro-level sectoral activities) and Figure S5 (emissions breakdown by sector) greatly clarifies the physical basis for the divergent aerosol responses across the three pathways. Furthermore, depositing the end-to-end code (emissions, namelists, pvlib pipeline) into a public GitHub repository is a massive step forward for reproducibility.

5. While a flat electricity price of \$0.09/kWh is admittedly a simplified proxy for a 2060 grid that will likely experience severe curtailment and time-of-use pricing volatility, the authors have appropriately caveated this. By framing this as a conservative estimate relative to the projected rise in systemic electricity supply costs, the economic scaling remains a useful, if heuristic, metric for policymakers.

Minor comments:

1) Ensure that all equations in the Methods section are sequentially numbered and properly formatted. For example, the Shapley value equations are listed as equations (3), (4), and (5) , but ensure equations (1) and (2) are distinctly formatted and referenced in the text.

2) Ensure the GitHub repository (https://github.com/shihrong/PV-China-2060) is set to "Public" and contains a clear README.md with instructions on how to execute the pvlib pipeline and WRF-Chem namelists. Consider minting a DOI for the repository using Zenodo for long-term archiving.

3) In the Methods section, there is a minor typo: "representing an alternative method to isolate cloud-aerosol forcing as proposed by Ghan's method 62." This should simply read "...as proposed by Ghan (2013)" or similar to avoid redundancy.

(Remarks on code availability)

Reviewer #2

(Remarks to the Author)
I do not have further comments.

(Remarks on code availability)
I hope the github page is not public.. that'swhy showing 404 error.

Reviewer #3

(Remarks to the Author)
I co-reviewed this manuscript with one of the reviewers who provided the listed reports. This is part of the Nature Communications initiative to facilitate training in peer review and to provide appropriate recognition for Early Career Researchers who co-review manuscripts.

(Remarks on code availability)
The codes are appropriate.

Reviewer #4

(Remarks to the Author)
Thank you for the effort devoted to clarifying the manuscript; however, I consider that the results remain insufficiently robust: The use of the ensemble mean does not adequately represent the underlying variability, which in turn constrains the response of the regional climate model (RCM), particularly given that only a single RCM configuration is employed. In this regard, I believe that the analysis should, at a minimum, include simulations with the RCM driven by multiple forcing datasets. In addition a 3-years simulation is short to evaluate cloud and aerorols impacts on GHI. Evaluation of GHI was made for a very short period. I understand that it was previously made in another work, but still needed.

(Remarks on code availability)
The code is not available, or I have not been able to access.

Open Access This Peer Review File is licensed under a Creative Commons Attribution 4.0 International License, which permits use, sharing, adaptation, distribution and reproduction in any medium or format, as long as you give appropriate credit to the original author(s) and the source, provide a link to the Creative Commons license, and indicate if changes were made.
In cases where reviewers are anonymous, credit should be given to 'Anonymous Referee' and the source.
The images or other third party material in this Peer Review File are included in the article's Creative Commons license, unless indicated otherwise in a credit line to the material. If material is not included in the article's Creative Commons license and your intended use is not permitted by statutory regulation or exceeds the permitted use, you will need to obtain permission directly from the copyright holder.
To view a copy of this license, visit https://creativecommons.org/licenses/by/4.0/

We thank the reviewers for their careful examinations and thoughtful comments. Our point-by-point responses are provided below in blue. Our new/modified text in manuscript is highlighted in red.

Reviewer #1 (Remarks to the Author):

I enjoyed the opportunity to read manuscript "NCOMMS-25-58082 - Carbon Neutrality Strategies Modulate Air Quality Effects on Solar Power Generation." The paper builds an integrated chain—C-GEM (policy/energy–economy) → WRF-Chem (air quality & meteorology) → pvlib PV model—to claim that China's "High Renewables" pathway in 2060 delivers ~9,850±1,766 GWh/yr extra PV generation (≈$0.89±0.16 bn/yr) relative to a baseline, largely via ACI rather than ARI. It further asserts these air-quality gains exceed material-driven PV efficiency improvements and are concentrated in eastern/central China. The topic is important, but the manuscript overreaches on identification, attribution, valuation, and uncertainty. As written, the policy-relevant economic conclusions are not credible.

Major Comments

1. The ACI vs ARI partition mainly infers "dominance" from GHI–GHI_c differences. That is not a causal ACI identification. You must run paired physics toggles (ARI-on/ACI-off; ACI-on/ARI-off) and microphysics/radiation scheme ensembles to isolate process contributions. Without them, "ACI dominates" is unsubstantiated and highly model-dependent.

R: We thank the reviewer for the insightful comment. We agree with the reviewer that diagnosing aerosol－cloud interaction (ACI) dominance solely based on GHI－GHI_c differences is insufficient for strict causal attribution. To address this concern, two sensitivity experiments were conducted for each scenario (Gao et al., 2023): (1) ACI-off experiment (ARI-on), in which aerosol–cloud coupling in cloud microphysics was disabled. The difference between CTRL and ACI-off therefore represents the radiative contribution arising specifically from aerosol–cloud interactions (ΔGHI_{ACI}). (2) ARI-off experiment (ACI-on), in which aerosol–radiation coupling in the radiative transfer scheme was suppressed. The difference between CTRL and ARI-off thus isolates the radiative contribution attributable to aerosol–radiation interactions (ΔGHI_{ARI}). The CTRL simulation is not a newly introduced scenario. It refers to the standard WRF-Chem configuration (with both ARI and ACI enabled) that has been used throughout the manuscript for all baseline and carbon-neutral pathway simulations.

The revised results show that our main conclusion remains unchanged. The process-level decomposition reveals a clear dominance of ACI. While ΔGHI_{ARI} shows a modest positive contribution (domain means of 0.37, 0.47, and 0.69 W m^{-2} for HB, CS, and HR, respectively),

the ΔGHI_{ACI} component is substantially larger, contributing 1.32, 1.64, and 2.66 W m^{-2}. Moreover, the fractional contribution of ACI increases progressively from HB to CS and reaches its maximum under the most stringent HR pathway. In all scenarios, ACI accounts for approximately three-quarters of the total radiation enhancement. The fractional contribution of ACI to total △GHI increases progressively from HB to CS and is largest under the HR scenario, indicating a strengthening dominance of indirect aerosol effects under more aggressive emission reductions.

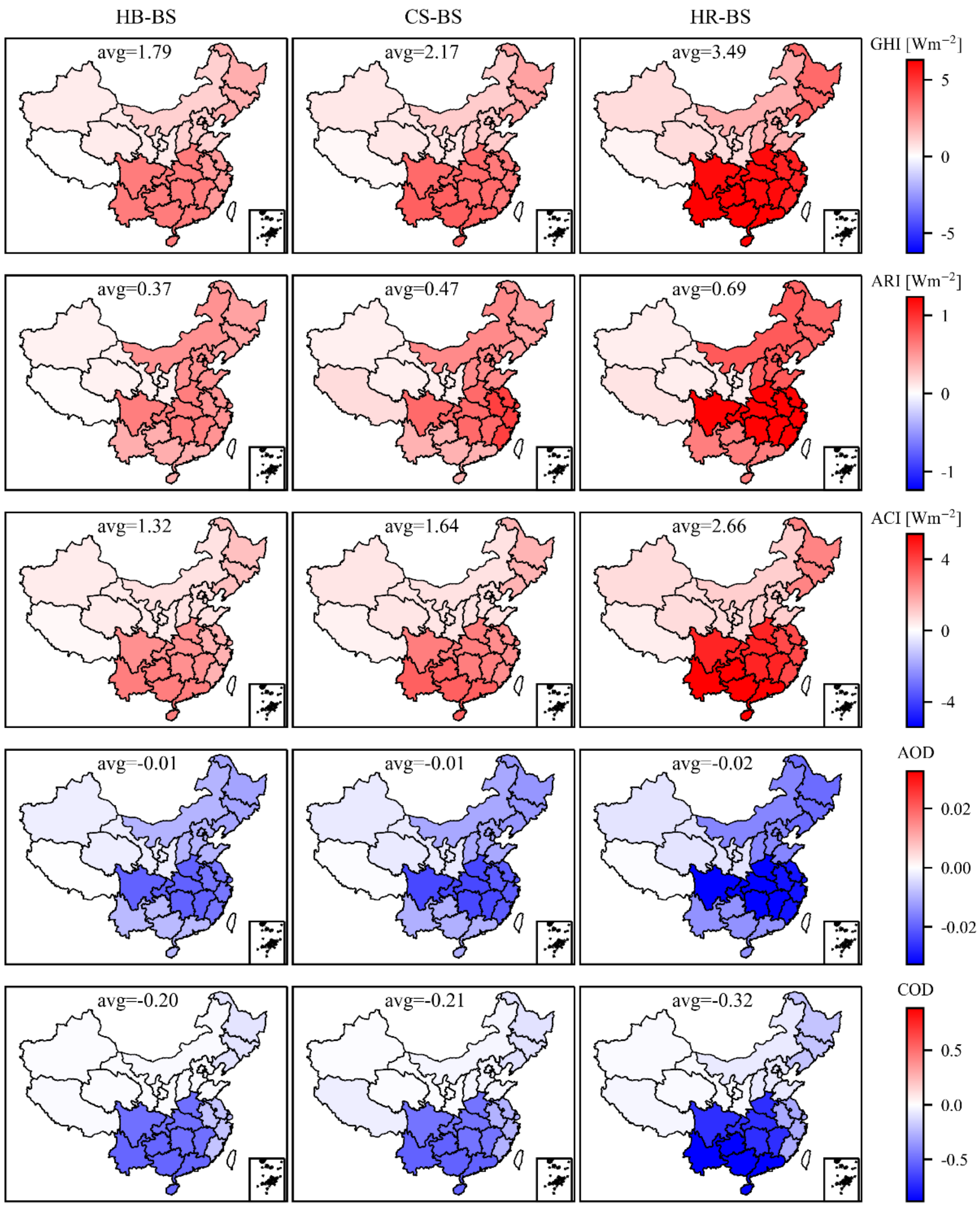

Figure 3. Spatial distribution of key atmospheric variables influencing GHI changes. This figure illustrates the spatial distribution in GHI changes and the corresponding

contributions from aerosol–radiation interactions (ARI) and aerosol–cloud interactions (ACI), aerosol optical depth (AOD), and cloud optical depth (COD) across China's seven regional power grids under three carbon neutrality pathways (HB, CS, and HR) relative to BS in 2060.

We have revised in Method part (line 458-483):

"Attribution of scenario-induced GHI changes

In this study, all baseline and carbon-neutral pathway simulations were conducted using the standard WRF-Chem configuration, in which both ARI and ACI are enabled. For clarity, we refer to this standard configuration as the CTRL simulation. To isolate the contributions of ACI and ARI to scenario-induced changes in GHI, we performed a set of paired sensitivity experiments. Two sensitivity experiments were conducted for each scenario, adopting the process-level separation approach detailed by Gao, et al. [40]: (1) ACI-off experiment (ARI-on), in which aerosol–cloud coupling in cloud microphysics was disabled. The difference between CTRL and ACI-off therefore represents the radiative contribution arising specifically from aerosol–cloud interactions ($\Delta\mathrm{GHI}_{ACI}$). (2) ARI-off experiment (ACI-on), in which aerosol–radiation coupling in the radiative transfer scheme was suppressed. The difference between CTRL and ARI-off thus isolates the radiative contribution attributable to aerosol–radiation interactions ($\Delta\mathrm{GHI}_{ARI}$). Under this differential framework, the total scenario-induced change in GHI can be approximately decomposed into ACI and ARI contributions.

Our attribution focuses on scenario-induced increments relative to the BS scenario, rather than absolute aerosol effects. For each future carbon-neutral scenario X (HR, HB, and CS), the total change in GHI relative to BS is defined as

$$\Delta\mathrm{GHI} = \mathrm{GHI}_{\mathrm{CTRL}}^{\mathrm{X}} - \mathrm{GHI}_{\mathrm{CTRL}}^{\mathrm{BS}} \tag{6}$$

The contribution from ACI is diagnosed as

$$\Delta\mathrm{GHI}_{ACI} = \left(\mathrm{GHI}_{\mathrm{CTRL}}^{\mathrm{X}} - \mathrm{GHI}_{\mathrm{ACIoff}}^{\mathrm{X}}\right) - \left(\mathrm{GHI}_{\mathrm{CTRL}}^{\mathrm{BS}} - \mathrm{GHI}_{\mathrm{ACIoff}}^{\mathrm{BS}}\right) \tag{7}$$

which represents the enhancement of GHI attributable to ACI under scenario X relative to BS.

Similarly, the contribution from ARI is defined as

$$\Delta\mathrm{GHI}_{ARI} = \left(\mathrm{GHI}_{\mathrm{CTRL}}^{\mathrm{X}} - \mathrm{GHI}_{\mathrm{ARIoff}}^{\mathrm{X}}\right) - \left(\mathrm{GHI}_{\mathrm{CTRL}}^{\mathrm{BS}} - \mathrm{GHI}_{\mathrm{ARIoff}}^{\mathrm{BS}}\right) \tag{8}$$

which represents the enhancement of GHI attributable to ARI under scenario X relative to BS."

References:

1. Gao, D., Zhao, B., Wang, S., Wang, Y., Gaudet, B., Zhu, Y., ... & Dong, Z. (2023). Increased importance of aerosol–cloud interactions for surface PM 2.5 pollution relative to aerosol–radiation interactions in China with the anthropogenic emission reductions. Atmospheric Chemistry and Physics, 23(22), 14359-14373.

2. Simulating only 2059–2061 cannot robustly represent the distribution of cloud/aerosol states or PV resources. At minimum, use decadal slices (e.g., 2051–2060) and/or multi-member perturbed-initial-condition ensembles to quantify sampling uncertainty.

R: We acknowledge the reviewer's concern regarding the representativeness of a 3-year simulation period.

The meteorological initial and boundary conditions in the future used bias-corrected ensemble mean SSP126 climate data from 18 global climate models (Xu et al., 2021). This dataset has two advantages, firstly, the multi-model ensemble mean reduces the uncertainty in climate sensitivity from a single model, secondly, the multi-model climate data, after being corrected against the reanalysis data, makes future projections more reasonable.

Besides, we simulated for three years (2059-2061) to reduce the impact of internal climate variability on ambient PM2.5 concentrations. Although a 3-year simulation period may not fully eliminate the influence of natural variability, we utilized ensemble meteorological fields derived from 18 models, which substantially mitigates the effects of low-frequency variability.

An important point to clarify is that all scenarios in this study were driven by identical meteorological conditions, with only emission rates differing between scenarios. Our analysis focuses on relative differences across emission scenarios rather than absolute meteorological or climate states, so the influence of interannual meteorological variability on our key conclusions is greatly minimized.

We also note that the reviewer's suggestion of using decadal simulations is commonly adopted in studies aimed at assessing climate change impacts, where long-term statistics of climate variability are essential. In contrast, studies evaluating the effects of emission changes on air quality under prescribed meteorological conditions typically employ much shorter simulation periods (e.g., Zhang et al., 2021; Cheng et al., 2021; Sun et al., 2024), since the primary objective is to isolate the response of atmospheric composition to emission differences rather than to characterize long-term climate variability. In this context, multi-year simulations of one to several years are widely used in policy-relevant air-quality modeling studies.

We have updated the **Method** section (Lines369-376):

"…While this timeframe may not capture decadal climatological shifts, it provides a robust

basis for isolating the radiative benefits of air quality improvements under diverse carbon-neutrality strategies. Our analysis focuses on relative differences across emission scenarios rather than absolute meteorological or climate states, so the influence of interannual meteorological variability on our key conclusions is greatly minimized. According to our literature review 24,48,49, the combination of an 18-model ensemble and a 3-year simulation period represents a more robust approach to addressing nature variability compared to most existing studies of a similar nature."

Reference

1. Cheng, J.; Tong, D.; Zhang, Q.; Liu, Y.; Lei, Y.; Yan, G.; Yan, L.; Yu, S.; Cui, R. Y.; Clarke, L.; et al. Pathways of China's PM2.5 air quality 2015-2060 in the context of carbon neutrality. Natl Sci Rev 2021, 8 (12). DOI: 10.1093/nsr/nwab078.

2. Sun, Y. S.; Jiang, Y. Q.; Xing, J.; Ou, Y.; Wang, S. X.; Loughlin, D. H.; Yu, S.; Ren, L.; Li, S. Y.; Dong, Z. X.; et al. Air Quality, Health, and Equity Benefits of Carbon Neutrality and Clean Air Pathways in China. Environ Sci Technol 2024, 58 (34), 15027-15037. DOI: 10.1021/acs.est.3c10076.

3. Zhang, S. H.; An, K. X.; Li, J.; Weng, Y. W.; Zhang, S. H.; Wang, S. X.; Cai, W. J.; Wang, C.; Gong, P. Incorporating health co-benefits into technology pathways to achieve China's 2060 carbon neutrality goal: a modelling study. Lancet Planet Health 2021, 5 (11), E808-E817. DOI: 10.1016/S2542-5196(21)00252-7.

4. Xu, Z. F., Han, Y., Tam, C. Y., Yang, Z. L. & Fu, C. B. Bias-corrected CMIP6 global dataset for dynamical downscaling of the historical and future climate (1979-2100). Sci Data 8, doi:10.1038/s41597-021-01079-3 (2021).

3. Future chemical initial/boundary conditions are proportionally reduced from today and tied to an SSP126 ensemble mean. This shortcut risks dynamical inconsistency between meteorology and composition. You need multi-GCM, multi-SSP chemistry–meteorology boundary experiments (or fully consistent CTM boundaries) with clear propagation of inter-model spread.

R: We thank the reviewer for raising this important point regarding the dynamical consistency between meteorology and chemical boundary conditions.

First, regarding meteorological forcing, future meteorological initial and boundary conditions were derived from a bias-corrected ensemble mean of 18 CMIP6 global climate models under SSP126 (Xu et al., 2021). This multi-model ensemble framework substantially reduces uncertainty associated with individual model climate sensitivity and large-scale

circulation biases, thereby improving the robustness of projected future meteorological states. Thus, the dynamical background driving WRF-Chem already incorporates inter-model spread at the climate level.

Second, SSP126 was selected because our objective is to evaluate China's carbon neutrality pathways under a globally consistent low-carbon development background (Riahi et al., 2016). SSP126 reaches net-zero CO_2 emissions globally around mid-century and exhibits cumulative emissions for China that closely align with those projected by our national carbon-neutral scenarios (as following figure). Therefore, using SSP126 ensures coherence between the global climate background and the domestic emission trajectories in our C-GEM framework.

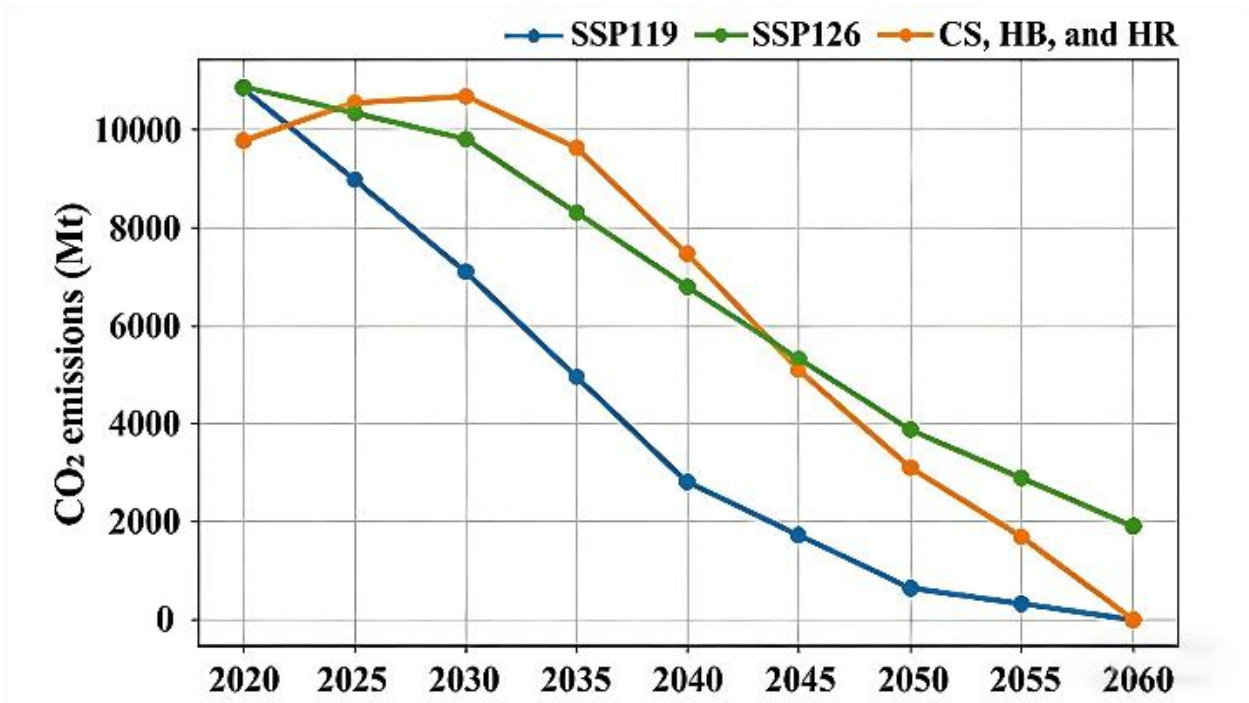

Figure. Emission of CO_2 in China under SSP119, SSP126, and the three carbon neutrality 310 scenarios (HB, CS, and HR).

For chemical initial and boundary conditions, future concentrations were derived by scaling present-day WACCM fields using the SSP126 2021–2060 concentration ratios. We adopted proportional scaling rather than absolute values because CMIP6 chemistry outputs from SSP126 scenario generally employ simplified chemical schemes and incompletely represent certain $PM_{2.5}$ components (particularly organic aerosols) (Emmons et al., 2020; Sellar et al., 2020; Wu et al., 2020). The ratio-based approach preserves reasonable large-scale concentration fields while maintaining consistency with the SSP126 global forcing trajectory.

Importantly, all carbon neutrality pathways examined in this study (HR, CS, HB) share identical global chemical boundary conditions. In our simulation design, the WRF-Chem model was reinitialized daily, with each run incorporating a 7-h spin-up period. This approach effectively minimizes potential deviations from the projected future meteorological fields that may arise in long term continuous simulations.

Besides, our analysis focuses on relative differences among scenarios (e.g., HR–BS, CS–BS, HB–BS), rather than absolute concentration projections. Under this framework, uncertainties associated with chemical boundary inflow are largely systematic across scenarios and therefore substantially cancel when calculating inter-scenario deltas. Consequently, the risk

of dynamical inconsistency affecting the core conclusions—namely the comparative impacts of different carbon neutrality pathways—is minimized.

We acknowledge that fully coupled multi-GCM × multi-SSP chemistry boundary experiments would provide a more comprehensive quantification of uncertainty propagation. However, such experiments would require coordinated global chemistry–climate model ensembles consistent with each SSP and are computationally intensive for high-resolution regional WRF-Chem simulations. Notably, our current experimental design is sufficient to meet the specific objectives of this study, as it effectively isolates the emission-driven impacts we aim to quantify.

We have clarified these methodological considerations in the revised manuscript (line358-367):

"

Future meteorological initial and boundary conditions were derived from a bias-corrected ensemble mean of 18 CMIP6 global climate models under the SSP126 scenario 47. This multi-model ensemble dataset was specifically developed for regional dynamical downscaling and has been shown to effectively reduce uncertainties associated with individual model climate sensitivity and large-scale circulation biases. By employing ensemble-mean climate forcing, the representativeness and robustness of projected future meteorological conditions are substantially improved, thereby providing a reliable basis for regional air-quality and PV-impact assessments.

Future chemical initial and boundary conditions were constructed by scaling present-day global chemical fields using concentration ratios between 2021 and 2060 under SSP126 (Gao et al., 2025). The reduced ratio was obtained from the changes in concentrations of $PM_{2.5}$ and its components from 2021 to 2060 under SSP126 in Institut Pierre-Simon Laplace Climate Model version 6A with low resolution Model (https://esgf-node.llnl.gov/search/cmip6/). This proportional-scaling approach preserves the large-scale spatial structure of chemical fields while ensuring consistency with the global low-emission background represented by SSP126. "

Reference:

1. Emmons, L. K., Schwantes, R. H., Orlando, J. J., Tyndall, G., Kinnison, D., Lamarque, J. F., ... & Pétron, G. (2020). The chemistry mechanism in the community earth system model version 2 (CESM2). *Journal of Advances in Modeling Earth Systems*, *12*(4), e2019MS001882.
2. Xu, Z. F., Han, Y., Tam, C. Y., Yang, Z. L. & Fu, C. B. Bias-corrected CMIP6 global dataset for dynamical downscaling of the historical and future climate (1979-2100). Sci Data 8,

doi:10.1038/s41597-021-01079-3 (2021).

3. Sellar, A. A., Walton, J., Jones, C. G., Wood, R., Abraham, N. L., Andrejczuk, M., ... & Griffiths, P. T. (2020). Implementation of UK Earth system models for CMIP6. *Journal of Advances in Modeling Earth Systems*, *12*(4), e2019MS001946.
4. Wu, T., Zhang, F., Zhang, J., Jie, W., Zhang, Y., Wu, F., ... & Hu, A. (2020). Beijing Climate Center Earth System Model version 1 (BCC-ESM1): model description and evaluation of aerosol simulations. *Geoscientific Model Development*, *13*(3), 977-1005.
5. Riahi, K., Van Vuuren, D. P., Kriegler, E., Edmonds, J., O'neill, B. C., Fujimori, S., ... & Tavoni, M. (2017). The Shared Socioeconomic Pathways and their energy, land use, and greenhouse gas emissions implications: An overview. *Global environmental change*, *42*, 153-168.

4. The claim that biases "cancel in differences" is an unsupported assumption. Perform site-wise bias correction (e.g., quantile mapping) with cross-validation, then re-run the PV chain; report how corrected vs uncorrected GHI alter PVPOT deltas and money metrics. Also quantify spatially structured error—differences do not guarantee cancellation.

R: We thank the reviewer for this important comment regarding the treatment of systematic biases in GHI and their potential impact on scenario-based differences.

Our analysis targets scenario-induced perturbations (e.g., HR/HB/CS relative to BS) rather than the absolute magnitude of surface GHI. All scenarios are simulated using the same model configuration, physical parameterizations, and boundary conditions, differing only in emission assumptions. Under this framework, systematic model biases in GHI are expected to be highly correlated across scenarios. As a result, while absolute GHI values in future projections may remain biased, the analysis of regional and inter-scenario differences becomes less sensitive to such systematic biases.

We have removed the problematic reasoning from the revised text to avoid any misleading interpretation. The specific revisions in the method part (line 383-386):

"

The WRF-Chem simulations show a consistent positive bias in GHI, with rMBE values falling within a narrow range of 8.6% – 11.5% across the seven grid regions. While absolute GHI values in future projections may remain biased, the analysis of inter-scenario differences is less sensitive to such systematic biases."

5. The "GHI contributes ~100%" result is constructed by one-at-a-time substitutions holding

other variables at baseline. Because PV output is nonlinear in (GHI, T, WS) and these fields are correlated, this approach misattributes interactions. Use Shapley value decomposition / ANOVA over factorial combinations to get a path-independent attribution with uncertainty.

R: We thank the reviewer for this insightful comment regarding attribution under nonlinear and correlated meteorological drivers. We agree that the one-at-a-time substitution approach, while useful for diagnostic sensitivity analysis, does not rigorously account for interaction effects when photovoltaic output depends nonlinearly on GHI, T2, and WS.

We applied a Shapley value – based attribution to decompose changes in PV_{POT} into contributions from GHI, T2, and WS. The method evaluates all factorial combinations of the three variables between baseline and scenario conditions, yielding a path-independent attribution that accounts for nonlinearity and interactions.

The revised analysis confirms that changes in GHI remain the dominant driver of PV_{POT} enhancement across all carbon neutrality pathways, while contributions from T2 and WS, as well as higher-order interaction effects, are comparatively minor. To avoid overinterpretation, we have revised the manuscript to replace the statement "GHI contributes ~100%" with a more precise description emphasizing the dominant role of GHI.

We have updated the Methods section (Line 438-455) accordingly and revised Fig. 2 to reflect the Shapley-based attribution results.

"

Contribution of GHI, T and WS on PV_{POT}

PV output responds nonlinearly to GHI, T2, and WS (Yang et al., 2022), traditional one-at-a-time approaches cannot provide a path-independent attribution of their contributions (Ndiaye et al., 2025; He and Zhao, 2025). Thus, to quantitatively analyze the contribution of GHI, T2, and WS to changes in PV_{POT}, a Shapley value–based decomposition method is employed in this study (Shapley, 1953). PV_{POT} is a nonlinear function of meteorological drivers,

$$PV_{POT} = f(GHI, T2, WS)$$

where f represents the full pvlib-based photovoltaic performance model chain.

For a specific driver $i \in N$ (here, $N = \{GHI, T2, WS\}$), its Shapley value ϕ_i representing its average marginal contribution to the total PV_{POT} change (ΔPV_{POT}), is defined as

$$\phi_i = \sum_{S \subseteq N \setminus \{i\}} \frac{|S|!\,(|N| - |S| - 1)!}{|N|!} [f(S \cup \{i\}) - f(S)]$$

where S denotes any subset of variables excluding i, and $|N| = 3$ in this study. This framework

ensures additive closure, such that:

$$\Delta PV_{POT} = \phi_{\mathrm{GHI}} + \phi_{T_2} + \phi_{WS}$$

For each carbon-neutrality pathway (HB, CS, HR), all $2^{|N|} = 8$ factorial combinations of baseline and scenario meteorological variables were constructed. PV_{POT} was evaluated for each combination using the pvlib model chain, and Shapley contributions were computed grid-point-wise through explicit enumeration of all marginal contributions.

”

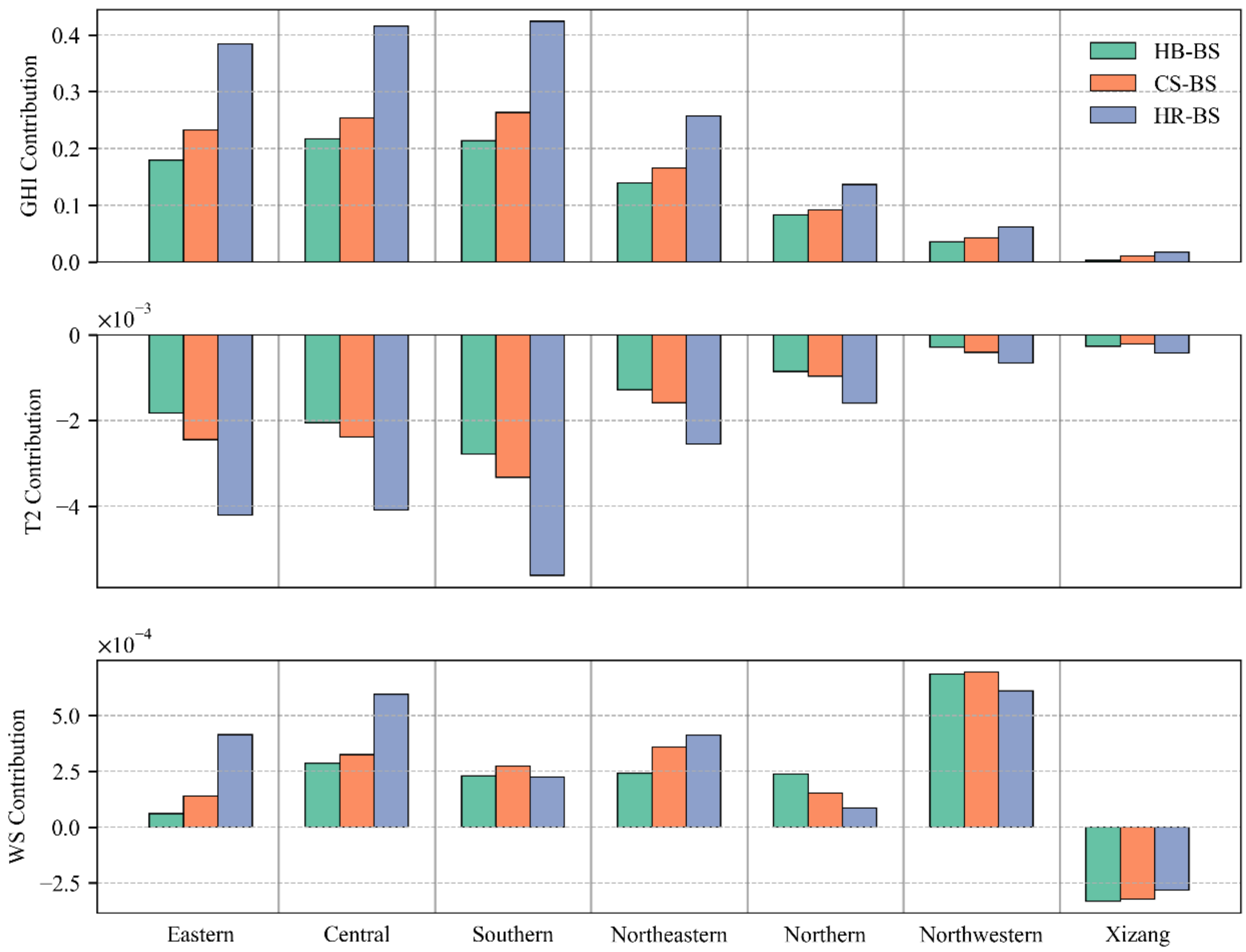

Figure 2. **Contributions of key meteorological variables to the changes in PV_{POT} [unit: %] across China's seven regional power grids under HB, CS, and HR compared to BS in 2060.** Key meteorological variables are GHI, T2, and WS. Seven power grids are Eastern, Central, Southern, Northeastern, Northern, Northwestern, and Xizang.

Reference:

1. Shapley, L. S. (1953). A value for n-person games. Contributions to the Theory of Games, 2(28), 307-317.
2. Yang, D., Wang, W., Gueymard, C. A., Hong, T., Kleissl, J., Huang, J., ... & Peters, I. M.

(2022). A review of solar forecasting, its dependence on atmospheric sciences and implications for grid integration: Towards carbon neutrality. Renewable and Sustainable Energy Reviews, 161, 112348.

3. Ndiaye, A., Faye, M., Gueye, D., Ba, A., & Traore, M. (2025). Analysis of the impact of irradiance and temperature on photovoltaic production: A statistical and machine learning approach. MethodsX, 103716.
4. He, B., & Zhao, C. (2025). A novel method to quantify the uncertainty contribution of aerosol–radiation interaction factors. *Atmospheric Chemistry and Physics*, *25*(14), 7765-7776.

6. The two-tier downscaling lacks inventory validation at sub-provincial scales and ignores plant siting and sector-specific controls. Provide ex-post checks against observed spatial AOD/$PM_{2.5}$ patterns and sectoral proxies; otherwise, PV benefits in high-demand east/central regions could be artifactually inflated.

R: We thank the reviewer for this important comment regarding the spatial credibility of the emission downscaling approach. To address these concerns, we have provided further details on our emission inventory construction and performed rigorous validation against observations.

First, C-GEM/ABaCAS-EI provides annual total anthropogenic emissions by province, sector, and species, including PM2.5, VOCs, SO2, NO_x, and NH3. VOC emissions are further speciated into SAPRC99 chemical mechanism species based on source profiles from ABaCAS-EI (Wu et al., 2017). The inventory covers a wide range of emission sectors, such as power generation, industrial combustion and processes, cement and steel production, residential fuel use (both fossil and biomass), on-road and off-road transportation, open burning, solvent use (industrial and domestic), agriculture (fertilizer application and livestock), and other domestic sources (Ding et al., 2019). To generate gridded hourly emission files for WRF-Chem simulations, emissions were temporally allocated (monthly, weekly, and hourly) based on activity data (Streets et al., 2003; Wang et al., 2005; Lin et al., 2010; Zhang et al., 2009), and spatially distributed using proxies derived from county-level GDP, high-resolution population data, and road network maps (Ding et al., 2020).

Second, we performed an ex-post validation using 2021 as a base year to ensure the spatial fidelity of our modeling system. We simulated the results for January, April, July, and October 2021 and compared them with the corresponding observational data (details in Figure S7 of Gao et al., 2025, as following). As shown in following figure, the model successfully captures the observed spatial gradients of $PM_{2.5}$ across China, particularly in the high-demand East and Central regions. Specifically, Table S2 shows the performance statistics for $PM_{2.5}$, including mean observation, mean bias simulation, normalized mean bias (MMB), normalized mean error

(NME), mean fractional bias (MFB), and mean fractional error (MFE). The NMBs of $PM_{2.5}$ concentrations are within 12% in January, April, July, while the maximum NMB occur in October, reaching only 23%. MFB and MFE for $PM_{2.5}$ meet the model performance goal proposed by Boylan and Russell [15].

The reasonable agreement in spatial patterns and statistical metrics suggests that our downscaled emission inventory provides a reliable baseline for investigating the radiative impacts of carbon neutrality pathways.

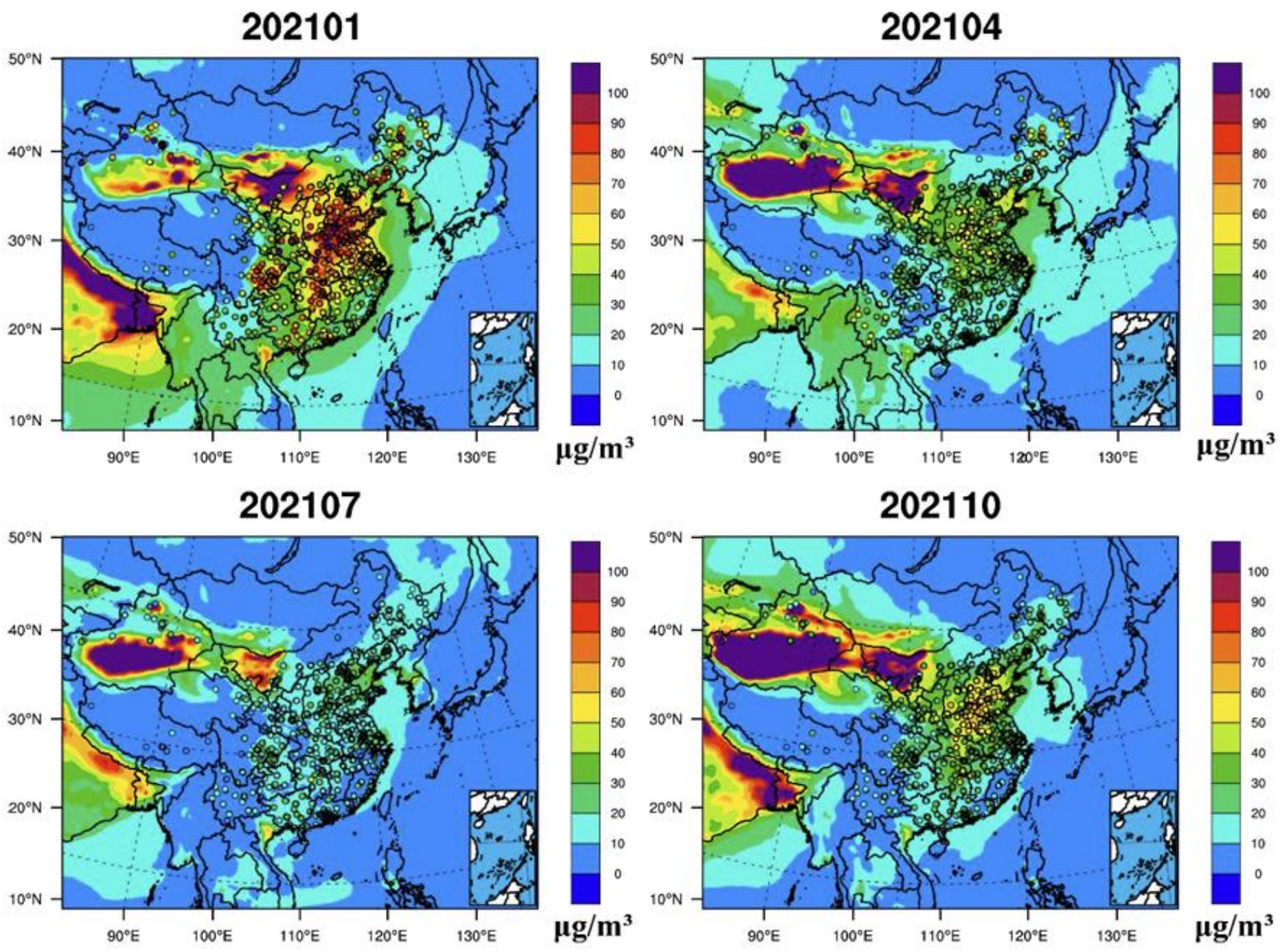

Figure S7. Observations (dots) and simulations (contours) of surface PM2.5 concentrations (unit: μg m−3) in January, April, July, and October 2021.

We have clarified these considerations in the revised manuscript (Line 342-348):

“

The anthropogenic emissions from the C-GEM/ABaCAS-EI framework were downscaled to a 27 km grid using a sector-specific approach. To generate gridded hourly emission files for WRF-Chem simulations, emissions were temporally allocated (monthly, weekly, and hourly) based on activity data (Streets et al., 2003; Wang et al., 2005; Lin et al., 2010; Zhang et al., 2009), and spatially distributed using proxies derived from county-level GDP, high-resolution population data, and road network maps (Ding et al., 2020). To ensure spatial reliability, the simulation results were validated against nationwide $PM_{2.5}$ observations for the base year of

2021. The spatial patterns of simulated $PM_{2.5}$ align well with observations (FigureS7 in ref. [17])

”

Reference

1. Lin, J. T.; McElroy, M. B.; Boersma, K. F., Constraint of anthropogenic NO_x emissions in China from different sectors: a new methodology using multiple satellite retrievals. *Atmos Chem Phys* 2010, *10*, (1), 63-78.
2. Streets, D. G.; Bond, T. C.; Carmichael, G. R.; Fernandes, S. D.; Fu, Q.; He, D.; Klimont, Z.; Nelson, S. M.; Tsai, N. Y.; Wang, M. Q.; Woo, J. H.; Yarber, K. F., An inventory of gaseous and primary aerosol emissions in Asia in the year 2000 - art. no. 8809. *J Geophys Res-Atmos* 2003, *108*, (D21).
3. Wang, X. P.; Mauzerall, D. L.; Hu, Y. T.; Russell, A. G.; Larson, E. D.; Woo, J. H.; Streets, D. G.; Guenther, A., A high-resolution emission inventory for eastern China in 2000 and three scenarios for 2020. *Atmos Environ* 2005, *39*, (32), 5917-5933.
4. Zhang, Q.; Streets, D. G.; Carmichael, G. R.; He, K. B.; Huo, H.; Kannari, A.; Klimont, Z.; Park, I. S.; Reddy, S.; Fu, J. S.; Chen, D.; Duan, L.; Lei, Y.; Wang, L. T.; Yao, Z. L., Asian emissions in 2006 for the NASA INTEX-B mission. *Atmos Chem Phys* 2009, *9*, (14), 5131-5153.
5. Ding, D. Response Surface Model of Atmospheric $PM_{2.5}$ and O_3 Concentration with Precursor Emissions and Its Application. Tsinghua University, 2020. (In Chinese)
6. Wu, W.; Zhao, B.; Wang, S.; Hao, J., Ozone and secondary organic aerosol formation potential from anthropogenic volatile organic compounds emissions in China. J Environ Sci (China) 2017, 53, 224-237.
7. Ding, D.; Xing, J.; Wang, S.; Liu, K.; Hao, J., Estimated Contributions of Emissions Controls, Meteorological Factors, Population Growth, and Changes in Baseline Mortality to Reductions in Ambient PM2.5 and PM2.5-Related Mortality in China, 2013-2017. Environ Health Perspect 2019, 127, (6), 067009.

7. Equation (3) labels both ΔPVPOT·Capacity_BS and ΔPVPOT·ΔCapacity as “aerosol-induced.” The second term depends on capacity expansion, which is driven by policy/tech economics—not aerosols per se. Treat pure resource effect and capacity siting/scale effect separately; else you over-attribute economic gains to air quality.

R: We thank the reviewer for this important conceptual clarification.

In this study, we define the gains in PV power generation from improved air quality under carbon neutrality pathways as

$$\Delta PV_{POT_{S-BS}} \cdot Capacity_S = \Delta PV_{POT_{S-BS}} \cdot Capacity_{BS} + \Delta PV_{POT_{S-BS}} \cdot \Delta Capacity_{S-BS} \quad (9)$$

where $\Delta PV_{POT_{S-BS}}$ represents the change in PV_{POT} between carbon neutrality pathway S (HB, CS, or HR) and the baseline scenario (BS) due to reduced aerosol loading, and $Capacity_S$ denotes the installed PV capacity under carbon neutrality pathway S. This term

therefore represents the PV generation gains attributable to improved air quality under the PV deployment scale of each carbon neutrality pathway.

This total gain consists of two components: a pure resource (air-quality) effect, $\Delta PV_{POT(S-BS)} \cdot Capacity_{BS}$, representing the increase in PV generation due solely to improved PV_{POT} under the baseline installed capacity; and an synergistic effect, $\Delta PV_{POT(S-BS)} \cdot \Delta Capacity_{(S-BS)}$, which reflects the additional gains from improved air quality realized under expanded PV deployment in the carbon neutrality pathways.

To address this concern, we have revised the attribution framework and terminology throughout the manuscript: Results (Line 178-192), Figure 4, and method parts (Line504-516).

8. Using a flat $0.09/kWh in 2060, no curtailment, no congestion, no LMP/capacity value differentiation, and no system integration (storage, transmission) overstates benefits. Replace with power-system–consistent valuation (e.g., marginal value of PV energy by region/hour from a capacity-expansion/UC-ED model) and apply discounting and price scenarios.

R: We thank the reviewer for this important comment. We fully agree that a flat electricity price assumption does not represent a fully power-system–consistent valuation, particularly under high renewable penetration in 2060, where electricity prices, curtailment, congestion, and system integration effects (e.g., storage and transmission) will play an increasingly important role.

To provide a transparent and conservative estimate, we adopted a constant average electricity price of 0.09 USD/kWh, which is broadly consistent with current wholesale electricity prices in China. As of 2021, reported residential and industrial electricity prices were approximately 0.084 and 0.089 USD/kWh, respectively (Zhuo et al., 2022). However, driven by the nation’s dual objectives of market reform and decarbonization, these rates are projected to experience a significant upward trajectory, primarily due to the growth of per capita GDP and the mandatory reduction in energy intensity (Zhuo et al., 2022).

Additionally, recent system-level modeling studies further suggest that electricity supply costs under carbon neutrality pathways will continue to rise by 2050 due to large-scale renewable deployment, grid expansion, and system integration requirements (Chen et al., 2021). Therefore, the adopted value of 0.09 USD/kWh represents a moderate and conservative estimate within the projected range of future electricity costs.

We have updated the manuscript to clarify these assumptions and provide additional context for the applied price estimate in Results (line 196-205):

“

Using an average electricity price of 0.09 USD/kWh, which reflects current market rates in China (approximately 0.084 USD/kWh for residential and 0.089 USD/kWh for industrial users as of 2021, Zhuo et al., 2022), we estimated the economic value of this additional generation. Driven by the dual objectives of market reform and decarbonization, these rates are projected to rise in the future due to factors such as GDP growth and the mandatory reduction in energy intensity (Zhuo et al., 2022). Additionally, recent system-level modeling studies suggest that electricity supply costs will continue to rise under carbon neutrality pathways, reaching higher levels by 2050 due to large-scale renewable energy deployment, grid expansion, and system integration requirements (Chen et al., 2021). Therefore, the adopted value of 0.09 USD/kWh represents a conservative estimate within the projected range of future electricity costs."

Reference:

1. Zhuo, Z., Du, E., Zhang, N., Nielsen, C. P., Lu, X., Xiao, J., ... & Kang, C. (2022). Cost increase in the electricity supply to achieve carbon neutrality in China. Nature communications, 13(1), 3172.
2. Qiu, Y., Zhou, S., Gu, W., & Zhang, X. P. (2023). Analysis of China's electricity price and electricity burden of basic industries under the carbon peak target before 2030. CSEE Journal of Power and Energy Systems, 10(2), 481-491.

9. EasyUQ treats GHI only. Missing: C-GEM tech shares, emission factors, wrf-chem physics choices, ACI parameterization, pvlib model choices, price paths, capacity trajectories. Provide a full Monte Carlo / multi-model ensemble and tornado plots of elasticities to key assumptions.

R: We appreciate the reviewer's comprehensive perspective on uncertainty propagation across the full modeling chain. In principle, a fully integrated Monte Carlo or multi-model ensemble spanning energy–economy scenarios, emission inventories, atmospheric chemistry–meteorology, PV performance modeling, and economic valuation would indeed be desirable, which is computationally prohibitive.

In PV energy estimates, solar radiation uncertainty is regarded as one of the most critical and largest sources of uncertainty (Prilliman et al., 2023). Among all components of the PV model chain, surface solar radiation (GHI) overwhelmingly dominates variability in PV potential, whereas uncertainties associated with pvlib model options, temperature coefficients, or wind-speed parameterizations are well documented to be second order relative to irradiance uncertainty. We therefore focus our quantitative uncertainty analysis on GHI.

Given these constraints, to quantify the GHI uncertainty associated with deterministic WRF-Chem simulations, we employed the EasyUQ framework, which transforms deterministic

WRF-Chem radiation outputs into calibrated, probabilistic GHI distributions. By leveraging a training archive of model output–outcome pairs (WRF-Chem outputs and ground-based observations), EasyUQ generates probabilistic GHI forecasts that reflect the inherent uncertainty in the simulation's historical performance (Walz et al., 2024).

We now clarify in the manuscript that these uncertainty ranges reflect GHI uncertainty, rather than a full system-wide uncertainty envelope, and discuss additional sources qualitatively. (Supplementary Text S2).

Reference:

1. Prilliman, Matthew J., Clifford W. Hansen, Janine M.F. Keith, Steven Janzou, Marios Theristis, Aaron Scheiner, and Ellis Ozakyol. 2023 (Revised 2024). *Quantifying Uncertainty in PV Energy Estimates Final Report*. Golden, CO: National Renewable Energy Laboratory (NREL). NREL/TP-7A40-84993.
2. Walz, E.-M., Henzi, A., Ziegel, J. & Gneiting, T. Easy Uncertainty Quantification (EasyUQ): Generating predictive distributions from single-valued model output. *Siam Review* **66**, 91-122 (2024).

10. This paper uses the north-easterly winds to estimate the impact of Korean air pollution on Chinese air quality and it would be recommended using all of the Easterly winds (Northeast, East, Southeast) to examine the changes in air pollution levels from Beijing to Shanghai. In the case of Southeast wind, how do the authors disentangle the spillover impacts of South Korea from Japan?

R: No relevant statement is found in the manuscript. We would appreciate the reviewer's clarification on the specific location.

11. You cite past "brightening" after 2013 policy, but do not validate the model chain against observed PV output (plant- or province-level) over 2015–2023 concurrent with measured PM reductions. A credible study should reproduce historical PV gains due to cleaned air before projecting 2060.

R: We sincerely appreciate the reviewer's suggestion to validate the full PV modeling chain against historical observations. We agree that verifying the model's ability to reproduce historical PV gains driven by air quality improvements is crucial for ensuring the credibility of future projections.

Recent studies have already quantified historical photovoltaic gains associated with

improved air quality in China using the same PV model chain framework employed in our study. For example, Song et al. (2024) and Ji et al. (2026) used the PVLIB-Python model to estimate PV capacity factors and generation changes associated with aerosol reductions following the Clean Air Action Plan (2013–2020). These studies show that improved air quality significantly enhanced PV power generation across China, particularly in eastern regions, with estimated additional generation of up to ~41.3 TWh yr^{-1} under current installed capacity.

Besides, the WRF-chem framework used in this study has been previously evaluated for historical conditions. Specifically, the WRF-Chem configuration, aerosol–radiation interaction scheme, and emission treatments used here are identical to those evaluated in our previous historical simulations, which successfully reproduced observed trends in aerosol optical depth and surface solar radiation over China during the post-2013 clean air period (Gao et al., ACP, 2023).

We therefore believe that re-simulating historical PV output would not materially strengthen the central conclusions of this study, which concern pathway-dependent differences in future PV benefits rather than the existence of the air-quality－PV effect itself.

We have clarified this scope and added relevant references to historical PV and brightening studies in the revised manuscript (Method line 406-410):

“

PV model chains for deriving PV_{POT} and power generation

…Previous studies have already quantified the historical enhancement of PV electricity generation associated with improved air quality in China following the Clean Air Action Plan, demonstrating substantial increases in solar radiation and PV output since 2013 (**Song et al., 2024; Ji et al., 2026**). Building upon this established modeling framework, the present study focuses on quantifying how future emission pathways influence PV generation through air-quality-mediated changes in solar radiation….

”

Reference:

1. Ji, X., Liu, Z., Mu, J., Han, Z., Wang, Z., Zhu, Y., ... & Zhang, Y. (2026). Boosting solar efficiency: the unforeseen benefit of China’s clean air action plan. *Environmental Research Letters*, *21*(1), 014012.
2. Song, Z., Cao, S., & Yang, H. (2024). Quantifying the air pollution impacts on solar photovoltaic capacity factors and potential benefits of pollution control for the solar sector in China. *Applied Energy*, *365*, 123261.

3. Gao, D., Zhao, B., Wang, S., Wang, Y., Gaudet, B., Zhu, Y., ... & Dong, Z. (2023). Increased importance of aerosol–cloud interactions for surface PM 2.5 pollution relative to aerosol–radiation interactions in China with the anthropogenic emission reductions. Atmospheric Chemistry and Physics, 23(22), 14359-14373.

12. HR/HB/CS are described qualitatively. You need transparent tabulation of sectoral activity, emissions by precursor, and control measures by region to justify the differential aerosol response that drives PV benefits—and a sensitivity to alternative feasible mixes within each pathway.

R: We thank the reviewer for the suggestion to enhance the transparency of our scenario designs. We fully agree that a quantitative characterization of sectoral activities and emission profiles is essential to justify the differential aerosol responses across the three pathways. Accordingly, we have incorporated detailed quantitative data and visualizations into the revised manuscript.

We have added Table S1 to summarize the macro-level sectoral activities for each pathway in 2060, including indicators such as total CO_2 captured by technology, non-fossil energy shares, and total electrification rates. These data, based on our previous framework (Gao et al., 2026), provide a transparent foundation for understanding the technological preferences of each pathway. Complementing this, Figure S5 provides a detailed breakdown of sectoral emissions for four major precursors SO_2, NO_x, VOCs, and $PM_{2.5}$ across industrial and domestic sectors.

These data clearly illustrate the divergent emission profiles, which in turn explains the distinct aerosol responses. For instance, the HR (high renewables) scenario exhibits the lowest emission levels due to its high electrification rate (95%) and non-fossil share (97%). Compared to the BS, HR leads to substantial decreases in SO_2, $PM_{2.5}$, NO_x, and VOCs emissions by 68%, 57%, 53%, and 28%, respectively. These reductions are primarily driven by the near-total elimination of fossil fuel combustion in the transportation, power, and industrial sectors.

In contrast, the HB (High Biomass) scenario, while achieving net-zero targets, exhibits a weaker reduction in $PM_{2.5}$ (only 28% lower than BS) due to the increased utilization of biomass in the domestic combustion sector, which leads to residual primary $PM_{2.5}$ emissions. The CS (Carbon Capture and Storage) scenario represents an intermediate level, with reduction ratios of 55%, 37%, 33%, and 21% for SO_2, $PM_{2.5}$, NO_x, and VOCs, respectively. This reflects a balance between maintaining approximately 10% fossil energy reliance (equipped with Carbon Capture and Storage) and implementing electrification in the residential and transport sectors.

Regarding the reviewer's concern about sensitivity to alternative mixes, the selected HR, HB, and CS pathways represent the "outer boundaries" of the technically and economically feasible decarbonization spectrum. By maximizing technological differentiation while adhering

to the national mandate of an 80% non-fossil energy share, we effectively capture the full range of sensitivity for aerosol-related radiative feedbacks. This boundary-case design ensures that the radiative impacts of any intermediate technological combinations would likely fall within the interval defined by our current results.

Finally, we emphasize that these emission data are integrated from the ABaCAS-EI inventory using technology-specific emission factors across 31 provinces, ensuring that our downscaling process accounts for regional differences in industrial structure. We have incorporated these detailed quantitative descriptions into Method of the revised manuscript and cited the relevant tables and figures in Supplementary to ensure full transparency.

Table S1. Summary of Key Characteristics of the Three Representative Carbon Neutrality Strategies in the year 2060

Carbon Neutrality pathways	**HB**	**CS**	**HR**
Total CO_2 captured in biomass burning per year ($GtCO_2$/year)	0.8	0.5	0.2
Total CO_2 captured by direct air capture technology per year ($GtCO_2$/year)	0.2	0.4	0.1
Total CO_2 captured in coal and gas electricity generation and industry	1.2	1.3	0
Total CO_2 captured per year ($GtCO_2$/year)	2.2	2.2	0.3
Share of non-fossil energy in primary energy consumption (%)	80	80	97
Wind and solar generations (PWh)	11	12	15
Electrification rate, i.e., share of electricity in final energy consumption (%)	65	67	95

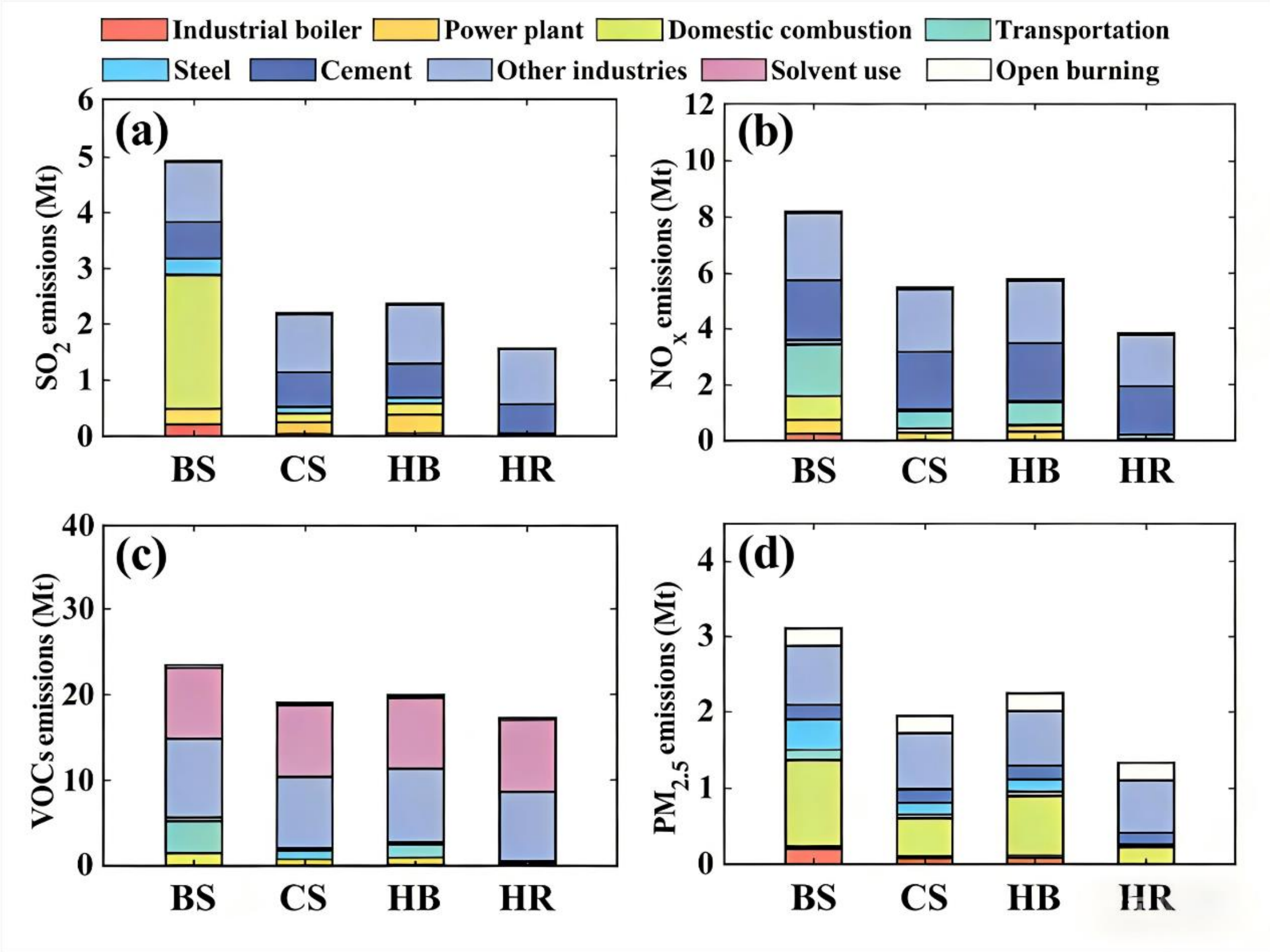

Figure S5. Air pollutant emissions under the baseline scenario (BS) and the three carbon neutrality scenarios (HB, CS, and HR) in 2060. (a) SO_2, (b) NO_x, (c) VOCs, and (d) $PM_{2.5}$ emissions contributed by different sectors under these four scenarios for 2060.

We have revised them in the Method part: line306-312 and line 320-328.

“

Carbon Capture and Storage (CS) featuring significant 20% fossil fuel reliance with Carbon Capture, Utilization, and Storage, High Biomass (HB) with expanded bioenergy use featuring 9% biomass energy share, and High Renewables (HR) dominated by wind and solar power accounting 70% of primary energy. The key socio-economic characteristics and sectoral activity levels for these pathways, including CO_2 capture amounts by technology, electrification rates, and energy mix compositions, are quantitatively summarized in Table S1.

…

As illustrated in Figure S5, these pathways result in markedly different sectoral emission profiles. For instance, the HR (high renewables) scenario exhibits the lowest emission levels due to its high electrification rate (95%) and non-fossil share (97%). Compared to the BS, HR leads to substantial decreases in SO_2, $PM_{2.5}$, NO_x, and VOCs emissions by 68%, 57%, 53%, and 28%, respectively. The HB scenario, exhibits a weaker reduction in $PM_{2.5}$ (only 28% lower

than BS) due to the increased utilization of biomass in the domestic combustion sector. The CS represents an intermediate level, with reduction ratios of 55%, 37%, 33%, and 21% for SO_2, $PM_{2.5}$, NO_x, and VOCs, respectively. These quantified sectoral differences provide the physical basis for the spatially heterogeneous aerosol responses and subsequent PV_{POT} changes analyzed in this study.

"

13. Lower COD and higher GHI could arise from dynamical changes (e.g., storm tracks, monsoon onset) unrelated to aerosol microphysics. Provide diagnostics (omega, RH profiles, cloud regimes) and causal mediation analysis before crediting ACI.

R: We sincerely thank the reviewer for this insightful comment. To isolate the role of aerosol microphysics from these dynamical factors and justify the credit given to ACI, we have performed the following additional analyses:

We conducted two sets of sensitivity experiments for all scenarios: (1) an ACI-off experiment (ARI-on) in which aerosol–cloud coupling in cloud microphysics was disabled, and (2) an ARI-off experiment (ACI-on) in which aerosol–radiation coupling in the radiative transfer scheme was suppressed. The CTRL simulation corresponds to the standard WRF-Chem configuration with both ARI and ACI enabled. Under this framework, the differences CTRL − ACI-off and CTRL − ARI-off isolate the radiative contributions from ACI and ARI, respectively.

Importantly, these sensitivity experiments are conducted under identical meteorological conditions and dynamical fields. As a result, we isolated the radiative contribution specifically arising from aerosol–cloud interactions ΔGHI_{ACI} and aerosol–radiation interactions ΔGHI_{ARI}. This allows us to explicitly quantify the GHI changes that are physically rooted in cloud microphysical coupling rather than large-scale dynamics. The corresponding spatial distributions of ΔGHI_ACI and ΔGHI_ARI are presented in Figure 3 of the revised manuscript.

Additionally, the simulated decreases in cloud droplet number concentration (NDROP) and liquid water path (LWP) (Supplementary Fig. S4) are consistent with the canonical aerosol–cloud interaction pathway, whereby reduced aerosol concentrations decrease CCN availability, leading to fewer cloud droplets, reduced cloud optical depth, and enhanced surface solar radiation.

We have clarified these methodological details and their implications in the revised manuscript to avoid potential misunderstanding.

14. PV value often hinges on peak-demand and cloudy/clear extremes. A 3-year slice misses

tail risks. Use longer climatologies and report extreme-percentile impacts on PV (e.g., P5/P95 of GHI and PV value).

R: We thank the reviewer for raising the important point regarding the potential influence of extreme cloudy or clear-sky conditions on PV performance. We acknowledge that a longer climatological record would provide a more comprehensive view of these extremes. However, the primary objective of this study is to quantify relative differences in PV_{POT} across carbon neutrality pathways, rather than to characterize the full climatological distribution of PV resources. We believe our current framework remains robust for the intended purpose.

Importantly, all scenarios in our study are driven by the same ensemble-based meteorological forcing, derived from the bias-corrected mean of 18 CMIP6 models under SSP126 (Xu et al., 2021). Consequently, the large-scale meteorological variability—including the occurrence of extreme cloudy or clear conditions—is consistent across scenarios. As a result, when calculating inter-scenario differences (e.g., HR–BS), the influence of internal climate variability and associated tail risks largely cancels out, allowing the aerosol-induced signal to be robustly isolated.

Furthermore, the three-year simulations (2059–2061) represent a commonly adopted compromise in regional chemistry–climate modeling studies due to the substantial computational expense of fully coupled WRF-Chem simulations with aerosol–cloud interactions. Similar simulation lengths have been widely used in policy-relevant assessments of future air quality and climate co-benefits (e.g., Zhang et al., 2021; Cheng et al., 2021; Sun et al., 2024).

We agree that longer climatological simulations would provide additional information on the full distribution of PV variability. However, given that our analysis focuses on scenario-induced changes rather than absolute PV climatology, the present framework remains appropriate for isolating the effects of aerosol-driven radiative changes on PV potential.

To clarify this point, we have added additional information in the manuscript (Method, Line 368-374).

“

…While this timeframe may not capture decadal climatological shifts, it provides a robust basis for isolating the radiative benefits of air quality improvements under diverse carbon-neutrality strategies. Our analysis focuses on relative differences across emission scenarios rather than absolute meteorological or climate states, so the influence of interannual meteorological variability on our key conclusions is greatly minimized. According to our literature review [24,48,49], the combination of an 18-model ensemble and a 3-year simulation period represents a

more robust approach to addressing nature variability compared to most existing studies of a similar nature.”

Reference:

1. Cheng, J.; Tong, D.; Zhang, Q.; Liu, Y.; Lei, Y.; Yan, G.; Yan, L.; Yu, S.; Cui, R. Y.; Clarke, L.; et al. Pathways of China's PM2.5 air quality 2015-2060 in the context of carbon neutrality. Natl Sci Rev 2021, 8 (12). DOI: 10.1093/nsr/nwab078.

2. Sun, Y. S.; Jiang, Y. Q.; Xing, J.; Ou, Y.; Wang, S. X.; Loughlin, D. H.; Yu, S.; Ren, L.; Li, S. Y.; Dong, Z. X.; et al. Air Quality, Health, and Equity Benefits of Carbon Neutrality and Clean Air Pathways in China. Environ Sci Technol 2024, 58 (34), 15027-15037. DOI: 10.1021/acs.est.3c10076.

3. Zhang, S. H.; An, K. X.; Li, J.; Weng, Y. W.; Zhang, S. H.; Wang, S. X.; Cai, W. J.; Wang, C.; Gong, P. Incorporating health co-benefits into technology pathways to achieve China's 2060 carbon neutrality goal: a modelling study. Lancet Planet Health 2021, 5 (11), E808-E817. DOI: 10.1016/S2542-5196(21)00252-7.

4. Xu, Z. F., Han, Y., Tam, C. Y., Yang, Z. L. & Fu, C. B. Bias-corrected CMIP6 global dataset for dynamical downscaling of the historical and future climate (1979-2100). Sci Data 8, doi:10.1038/s41597-021-01079-3 (2021).

15. The statement that air-quality PV gains are “~4× material-driven efficiency progress” cherry-picks a 0.5%/yr heuristic and ignores system-level module and BOS developments (bifacial, trackers, albedo, wiring, inverters) and learning rates to 2060. Provide a range from credible tech roadmaps and compare system yield, not a single efficiency proxy.

R: We thank the reviewer for this important comment. We agree that photovoltaic performance improvements arise not only from cell conversion efficiency but also from system-level innovations such as bifacial modules, tracking systems, inverter optimization, and other balance-of-system developments. In the revised manuscript, we have removed the comparison based on a single efficiency improvement rate and instead placed our results in the broader context of PV technology roadmaps. We replaced the 0.5%/yr heuristic with a comprehensive overview of PV evolution. This includes the transition from PERC to advanced HJT/TOPCon cells and the potential of perovskite tandems, as well as balance-of-system (BOS) improvements such as single-axis tracking and higher DC/AC ratios.

We have updated the manuscript (Discussion lines 253-266) to reflect this nuanced

comparison, ensuring it aligns with current industry roadmaps and avoids "cherry-picking" single-proxy metrics.

"

under the HR pathway, the national-average PV_{POT} increases by 0.36 percentage points, from 20.2% in the BS scenario to about 20.6%, equivalent to a ~2% relative increase. To contextualize this gain within the broader trajectory of solar technology, we compare it against industry benchmarks from the International Technology Roadmap for Photovoltaic (ITRPV). Over the past decade (2010–2020), standard mono-crystalline module efficiencies improved from approximately 15% to 20% [29], driven by the maturation of Passivated Emitter Rear Cell (PERC) technology, representing a decade-long average annual increase of 0.5% per year. Looking toward 2030, the roadmap projects further systematic enhancements: average module efficiencies are expected to reach 22.5%–24.0% through the adoption of Tunnel Oxide Passivated Contact (TOPCon) and Heterojunction (HJT) architectures, with perovskite/c-Si tandem cells creating a pathway toward 30% efficiency [30,31], expecting to average 0.3%–1% per year. Crucially, modern PV progress extends beyond cell efficiency to include system-level innovations that boost annual energy yield. Key innovations including single-axis tracking, bifacial modules, and optimized DC/AC inverter ratios (rising from 1.2 to 1.7) collectively boost annual energy yield. When aggregated, the combined effect of material and system-level advancements yields an overall system yield improvement of 0.8% – 1.2% per year according to industry benchmarks (ITRPV, 2019). …

"

Reference:

1. Victoria, M., Haegel, N., Peters, I. M., Sinton, R., Jäger-Waldau, A., Del Cañizo, C., ... & Smets, A. (2021). Solar photovoltaics is ready to power a sustainable future. *Joule*, *5*(5), 1041-1056.
2. International Technology Roadmap for Photovoltaic (ITRPV) 11th Ed. 2019 results. https://itrpv.vdma.org/documents/ 27094228/29066965/ITRPV%302020.pdf/ ba3da187-3186-83de-784e-6e3b10d96f3f.
3. Wilson, G.M., Al-Jassim, M., Metzger, W.K., Glunz, S.W., Verlinden, P., Xiong, G., Mansfield, L.M., Stanbery, B.J., Zhu, K., Yan, Y., et al. (2020). The 2020 photovoltaic technologies roadmap.J. Phys.D:Appl. Phys. 53, 493001.

16. There is no formal statistical design for causal attribution (only scenario contrasts). Where you report ±1σ, clarify what stochastic source it represents; then add block bootstrap over time and space to reflect field correlation, and clustered uncertainty by region.

R: We thank the reviewer for the sophisticated feedback on our statistical approach.

First, we clarify the definition and specific calculation methodology of the ±1σ. In short, ±1σ represent the statistical spread of the simulated PV potential changes from errors in simulated GHI in WRF-Chem.

In PV energy estimates, GHI uncertainty is regarded as one of the most critical and largest sources of uncertainty (Prilliman et al., 2023), while the influence of other variables is comparatively smaller. (Mayer & Yang, 2023). We therefore focus our quantitative uncertainty analysis on GHI.

To quantify this uncertainty, we applied the EasyUQ framework, which statistically post-processes deterministic WRF-Chem outputs to generate probabilistic GHI estimates based on model–observation pairs (Walz et al., 2024). These probabilistic GHI distributions were then propagated through the PV model chain to estimate the resulting uncertainty in PVPOT and PV power generation. The reported ±1σ values correspond to one standard deviation derived from the EasyUQ-calibrated predictive distributions.

For scenario contrasts (e.g., pathway-BS), we conservatively assume stochastic independence between scenarios when propagating uncertainty, such that Var(scenario-BS) = Var(scenario) + Var(BS). As a result, the reported ±1σ ranges should be interpreted as conservative upper-bound estimates of uncertainty.

Despite the robust nationwide gains identified for scenario contrasts, we acknowledge certain limitations in our uncertainty characterization. While our national-level assessment provides a clear signal for macro-scale energy policy, it may mask regional heterogeneities in model performance. Future research employing spatially-clustered bootstrapping or multi-model ensembles could further refine these local-scale uncertainties and provide more granular insights for regional grid planning.

We have revised in method part (Line529-536):

"

To quantify the uncertainty associated with projected PV_{POT}, PV power generation, and economic benefits increase under different carbon neutrality pathways, we focus on quantify the uncertainty associated with GHI, which uncertainty is regarded as one of the most critical and largest sources of uncertainty in PV energy estimates [60]. While uncertainties may arise from multiple components of the integrated modeling framework—including energy–economy scenarios, emission inventories, atmospheric chemistry–meteorology simulations, PV performance models, and economic valuation—a comprehensive quantification of all sources remains challenging. A detailed discussion of these uncertainty sources and their

limitations is provided in Text S2.

”

17. Numerous maps and comparisons are made without FWER/FDR control. Report field significance and False Discovery Rate for spatial patterns.

R: We thank the reviewer for pointing out the issue of multiple testing in spatial analyses. We have now assessed grid–level statistical significance using false discovery rate (FDR) control. For each grid cell, the significance of scenario differences was assessed using a paired t-test based on three-year simulations (2059-2061), and the FDR was controlled using the Benjamini–Hochberg procedure at $q = 0.05$. We applied FDR control to key spatial fields directly linked to PV performance (GHI and PV_{POT}). Variables with comparatively minor contributions (e.g., T2 and wind speed) are not subjected to grid-cell–level multiple-testing correction.

Our main figures focus on regionally averaged results relevant to power-grid–scale assessments. To address the reviewer's concern regarding spatial multiple testing, we additionally evaluated grid-cell–level significance with FDR control and provide the corresponding results in Fig. S1. While many grid cells satisfy nominal significance ($p < 0.05$), the FDR correction substantially reduces the number of significant cells, particularly under the HB and CS scenarios. Under the HR scenario, however, a substantial number of grid cells remain FDR-significant. Overall, the regional-mean conclusions remain robust, while the additional FDR analysis provides a more conservative and statistically rigorous interpretation of spatial patterns.

In Supplementary:

“

Text S1. Field significance with FDR control

To assess the robustness of spatial patterns and account for multiple testing across grid cells, we evaluated statistical significance using false discovery rate (FDR) control. Although a large number of grid cells satisfy nominal significance ($p < 0.05$) in all scenarios, the number of FDR-significant cells is substantially reduced, indicating limited spatial coherence at the grid scale.

For GHI, 4,273 and 4,769 grid cells satisfy $p < 0.05$ under the HB and CS scenarios, respectively, yet none (HB) or only two (CS) remain significant after FDR correction. In contrast, under the HR scenario, 189 grid cells remain FDR-significant, suggesting more spatially consistent changes in irradiance.

A similar but more pronounced pattern is found for PV_{POT}. While more than 5,000 grid cells satisfy $p < 0.05$ under each scenario, only one to two grid cells remain FDR-significant under HB and CS, whereas 1,058 grid cells remain significant under the HR scenario. The

concentration of FDR-significant PV_{POT} changes over eastern and southern China supports the robustness of the regional-mean enhancement identified in the main text.

Overall, the FDR analysis highlights the importance of accounting for multiple testing in spatial analyses and confirms that statistically robust grid-level signals are mainly confined to the HR scenario.

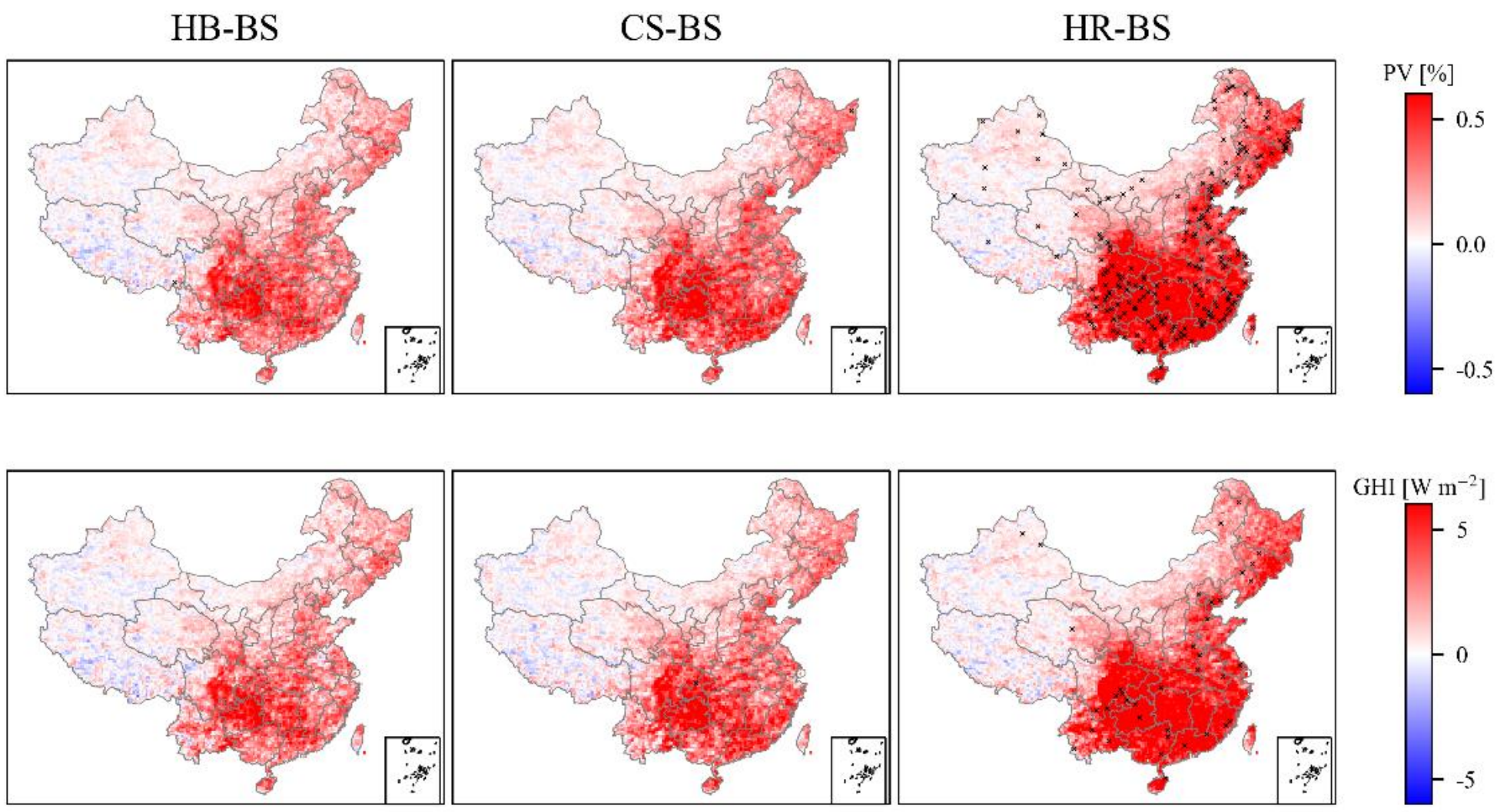

Figure S1. Spatial distributions of differences in the PV potential (PV_{POT}) and global horizontal irradiance (GHI) under three carbon neutrality pathways (HB, CS, and HR) relative to BS in 2060. Color shading denotes grid-cell–level differences, while black dots indicate grid cells that remain statistically significant after controlling the false discovery rate (FDR) at $q = 0.05$ using the Benjamini–Hochberg procedure, based on paired *t*-tests applied to annual means from three-year simulations (2059–2061). Only regions passing the FDR criterion are marked as statistically significant in the figures.

”

18. Standard errors that ignore spatial autocorrelation are too small. Use Conley-type or random field corrections when aggregating to regional effects.

R: We thank the reviewer for raising the important point of spatial autocorrelation in uncertainty estimation. In this study, however, the reported uncertainty does not arise from spatial sampling across grid cells. Instead, it reflects predictive uncertainty in GHI simulations estimated from WRF-Chem and subsequently propagated through the PV performance model chain.

Because the uncertainty originates from probabilistic forecasts rather than spatial sampling variability, the reported standard errors are not affected by spatial autocorrelation among neighboring grid cells. Therefore, corrections such as Conley-type or random-field adjustments

are not required for the uncertainty estimates presented here.

We have clarified this ±1σ definition, uncertainty source, and limitations in the Methods (Uncertainty analysis) and Supplementary TextS2.

19. Hold out subsets of ground stations and assess predictive skill of GHI/PVPOT deltas before monetization.

R: We thank the reviewer for this suggestion. We would like to clarify that the objective of this study is not to statistically predict future GHI or PV_{POT} based on a subset of observations, but to quantify scenario-driven differences in PV performance under a consistent, physics-based modeling framework.

Our approach follows a standard counterfactual scenario-comparison paradigm: the air-quality–PV response is simulated using the same WRF-Chem configuration across all scenarios, and future impacts are assessed based on relative differences between scenarios, rather than absolute irradiance values. As such, the key requirement is that the model can reasonably reproduce historical GHI magnitudes and spatial patterns, which we evaluated against 132 ground-based stations for an independent historical year (2021), yielding a regional-mean rMBE of ~10%. This level of agreement is comparable to, or better than, that reported in previous WRF-Chem based solar resource assessments.

Importantly, because all future scenarios are driven by the same meteorological boundary conditions and model physics, systematic model biases are expected to be largely consistent across scenarios. These biases therefore cancel to first order when computing inter-scenario differences (e.g., HR–BS), which are the basis for the reported ΔGHI, ΔPV_{POT}, and subsequent economic estimates.

We have revised the Method (Line389-393) to explicitly clarify this distinction between historical model evaluation and future scenario comparison, and to emphasize that monetized benefits are derived from physically consistent scenario-relative changes rather than absolute predictions at individual stations.

"Importantly, our analysis focuses on scenario-induced changes relative to the BS under a consistent physical modeling framework, rather than on the absolute magnitude of GHI. Consequently, when comparing regional differences across future scenarios, the analysis becomes less sensitive to pervasive systematic biases, as these biases are highly correlated across scenarios within the same modeling framework."

Minor Comments

1. Distinguish absolute vs relative PVPOT changes (e.g., 0.36% absolute ≈ 2% relative to 20.2%).

R: Thank you for your valuable comment. In the revised manuscript, we have explicitly clarified that under the HR pathway, the national-average PV_{POT} increases by 0.36 percentage points in absolute terms, from 20.2% in the BS scenario to approximately 20.6%. This corresponds to a relative increase of about 2% relative to the BS value of 20.2%. We have revised the text accordingly to ensure this distinction is clear to readers. We appreciate your suggestion to improve the precision of our presentation.

2. Add joint AOD–COD–GHI scatter and regional violin plots with uncertainty bands; show difference-of-bias-corrected results.

R: Thank you for this helpful suggestion.

First, we included joint scatter plots of ΔAOD–ΔGHI with ΔCOD indicated by color to examine the combined influence of aerosol and cloud changes on surface solar radiation. These plots (Fig. S3) show that reductions in AOD and COD are consistently associated with increases in GHI across regions and scenarios.

Second, we added regional violin plots of ΔGHI (Fig. S2) to illustrate the spatial distribution and variability of GHI changes across 7 power regions of China. The violin plots display the full distribution of grid-level values, along with the median and interquartile range, highlighting significant regional contrasts in the magnitude of GHI enhancement.

These new analyses are provided in Supplementary Figure S3 and Figure S2 and referenced in the Results section.

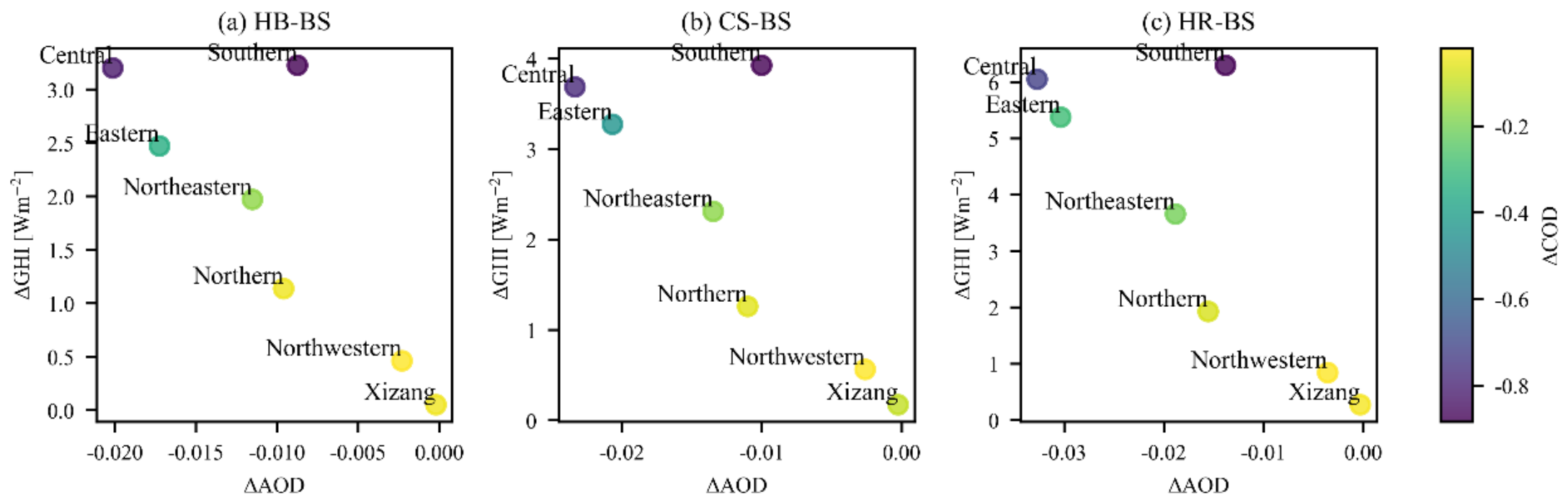

Figure S3. Joint relationships between aerosol, cloud, and solar radiation changes under different carbon neutrality pathways.

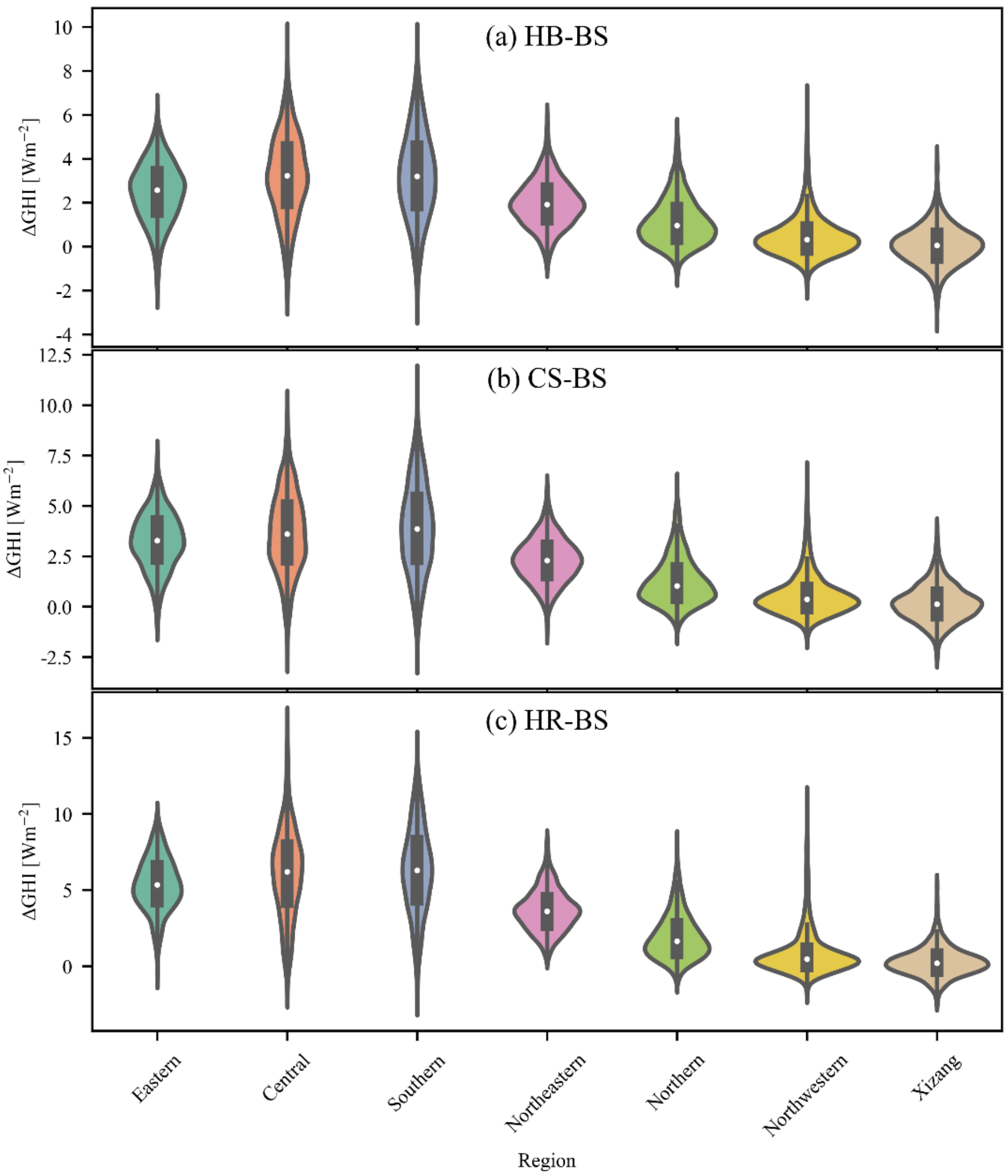

Figure S2. Regional distribution of simulated changes in surface solar radiation.

3. Report curtailment assumptions and co-located storage shares by region.

R: We thank the reviewer for raising this important point. In this study, PV power generation is estimated based on PV potential (PVPOT) and installed capacity, representing the resource-driven generation potential under each scenario. Grid integration factors such as curtailment and co-located storage deployment are not explicitly represented in the current framework.

Curtailment and storage deployment depend strongly on regional power system operation, transmission capacity, and storage investment strategies, which are typically simulated using dedicated power system models. Incorporating these processes is beyond the scope of the present study.

Nevertheless, previous studies suggest that increasing storage deployment and grid

flexibility under carbon neutrality pathways is expected to reduce renewable curtailment rates over time, particularly with continued expansion of transmission infrastructure and energy storage systems.

We have now clarified this assumption and added a discussion of this limitation in the revised manuscript (Method line 518-519).

4. “Available upon request” is inadequate. Provide public repos (C-GEM scenarios, downscalers, WRF-Chem namelists, pvlib pipeline) and replication scripts end-to-end.

R: We fully agree with the reviewer that transparency and reproducibility are essential for scientific advancement. In response, we have archived all the necessary resources and source code used in this study to a public repository (available at: https://github.com/shihrong/PV-China-2060), including emission data, namelist, pvlib code, visualization code, and so on.

The manuscript addresses a consequential question but currently relies on fragile modeling shortcuts and overstated economic claims. With rigorous identification, expanded ensembles, power-system-consistent valuation, and open replication, it could make a meaningful contribution-but not in its present form.

Reviewer #1 (Remarks on code availability):

No codes are available for review

Reviewer #2 (Remarks to the Author):

The authors have presented a study addressing a highly relevant topic at the intersection of carbon neutrality, air quality, and solar energy, with a focus on one of the most polluted and climate-sensitive regions, China. Overall, the manuscript is well written; however, I have the following comments.

1. The abstract is long. Should be trimmed in accordance with Nature journal.

R: Thanks for the suggestion.

“

Solar photovoltaics (PV) are pivotal for achieving carbon neutrality, yet their output is highly sensitive to air quality changes under future emission pathways. By integrating energy-economy, coupled meteorology-chemistry, and PV performance models, we quantify the impacts of future air quality driven by China’s different carbon neutrality pathways on PV generation. We show that a pathway emphasizing renewable energy deployment yields the greatest air quality improvements, boosting annual PV generation by 57,526 ± 10,314 GWh relative to business-as-usual—equivalent to $5.18 ± 0.93 billion in economic gains by 2060. This enhancement is primarily driven by aerosol-cloud interactions rather than direct aerosol-radiation effects. In contrast, pathways relying on biomass or carbon capture reduce these benefits by roughly two thirds. Spatially, economically developed regions with high energy demand, such as eastern and South China, benefit disproportionately from this enhancement. Our results underscore the synergies between decarbonization and air quality, advocating for policy frameworks that co-optimize pollutant mitigation to maximize renewable energy returns.

”

2. What are the 2060 carbon-neutral pathways referred to in the study? Are these scenarios specifically developed for China using C-GEM, or do they represent global trajectories? It would be helpful to illustrate this with a brief description of the corresponding emission reduction pathways and its difference w.r.t BS scenario. Although these scenarios might have been developed in earlier studies, including a concise discussion in the Methods section (or Supplementary Information) would enhance the completeness and standalone clarity of the paper (just like Table 1 or spatial map).

R: We thank the reviewer for this helpful suggestion. The carbon-neutral pathways analyzed in this study are China-specific scenarios developed using the China-in-Global Energy Model (C-GEM), which simulates economy-wide energy transitions and emission trajectories for China under alternative decarbonization strategies.

In addition to the Baseline Scenario (BS), which assumes no explicit carbon-neutrality constraint by 2060, we analyze three alternative carbon-neutral pathways that all achieve net-zero CO_2 emissions in 2060 but differ in their dominant decarbonization technologies: High Renewables (HR), High Biomass (HB), and Carbon Capture and Storage (CS).

To clarify the differences between these pathways, we have added Table S1 summarizing macro-level sectoral indicators in 2060, including electrification rates, non-fossil energy shares, and total CO_2 captured by technology. In addition, Figure S1 provides the sectoral emissions of major air pollutants (SO_2, NOx, VOCs, and $PM_{2.5}$) across industrial and residential sectors.

The three pathways (HR, HB, and CS) represent distinct technological archetypes spanning the feasible decarbonization spectrum, while all meeting the national requirement of achieving at least 80% non-fossil energy share by 2060. By maximizing technological differentiation under this common constraint, these scenarios effectively capture the plausible range of emission trajectories and associated aerosol responses. Consequently, the radiative impacts of intermediate technology mixes are expected to fall within the bounds defined by these scenarios.

We have added a concise description of these carbon-neutral pathways in the Methods section (line308-330) and included Table S1 and Figure S5 in the Supplementary Information to provide a transparent overview of the underlying emission trajectories and sectoral characteristics.

“

Energy-Economic Model and four scenarios for China’s carbon neutrality pathways

We utilize the China-in-Global Energy Model (C-GEM), …to assess China-specific carbon neutrality pathways and associated emission trajectories toward the national 2060 carbon-neutrality target…

We examine four China-specific scenarios: (1) a Baseline Scenario (BS) without carbon neutrality target in 2060; and three carbon-neutral pathways achieving net-zero emissions by 2060 through distinct technological emphases—Carbon Capture and Storage (CS) featuring significant 20% fossil fuel reliance with Carbon Capture, Utilization, and Storage, High Biomass (HB) with expanded bioenergy use featuring 9% biomass energy share, and High Renewables (HR) dominated by wind and solar power accounting 70% of primary energy. The detailed scenario designs are based on Gao, et al. [17]. The key socio-economic characteristics and sectoral activity levels for these pathways, including CO_2 capture amounts by technology, electrification rates, and energy mix compositions, are quantitatively summarized in Table S1.

… As illustrated in Figure S1, these pathways result in markedly different sectoral emission

profiles. For instance, the HR (high renewables) scenario exhibits the lowest emission levels due to its high electrification rate (95%) and non-fossil share (97%). Compared to the BS, HR leads to substantial decreases in SO_2, $PM_{2.5}$, NO_x, and VOCs emissions by 68%, 57%, 53%, and 28%, respectively. The HB scenario, exhibits a weaker reduction in $PM_{2.5}$ (only 28% lower than BS) due to the increased utilization of biomass in the domestic combustion sector. The CS represents an intermediate level, with reduction ratios of 55%, 37%, 33%, and 21% for SO_2, $PM_{2.5}$, NO_x, and VOCs, respectively. These quantified sectoral differences provide the physical basis for the spatially heterogeneous aerosol responses and subsequent PV_{POT} changes analyzed in this study."

Table S1. Summary of Key Characteristics of the Three Representative Carbon Neutrality Strategies in the year 2060

Carbon Neutrality pathways	**HB**	**CS**	**HR**
Total CO_2 captured in biomass burning per year ($GtCO_2$/year)	0.8	0.5	0.2
Total CO_2 captured by direct air capture technology per year ($GtCO_2$/year)	0.2	0.4	0.1
Total CO_2 captured in coal and gas electricity generation and industry	1.2	1.3	0
Total CO_2 captured per year ($GtCO_2$/year)	2.2	2.2	0.3
Share of non-fossil energy in primary energy consumption (%)	80	80	97
Wind and solar generations (PWh)	11	12	15
Electrification rate, i.e., share of electricity in final energy consumption (%)	65	67	95

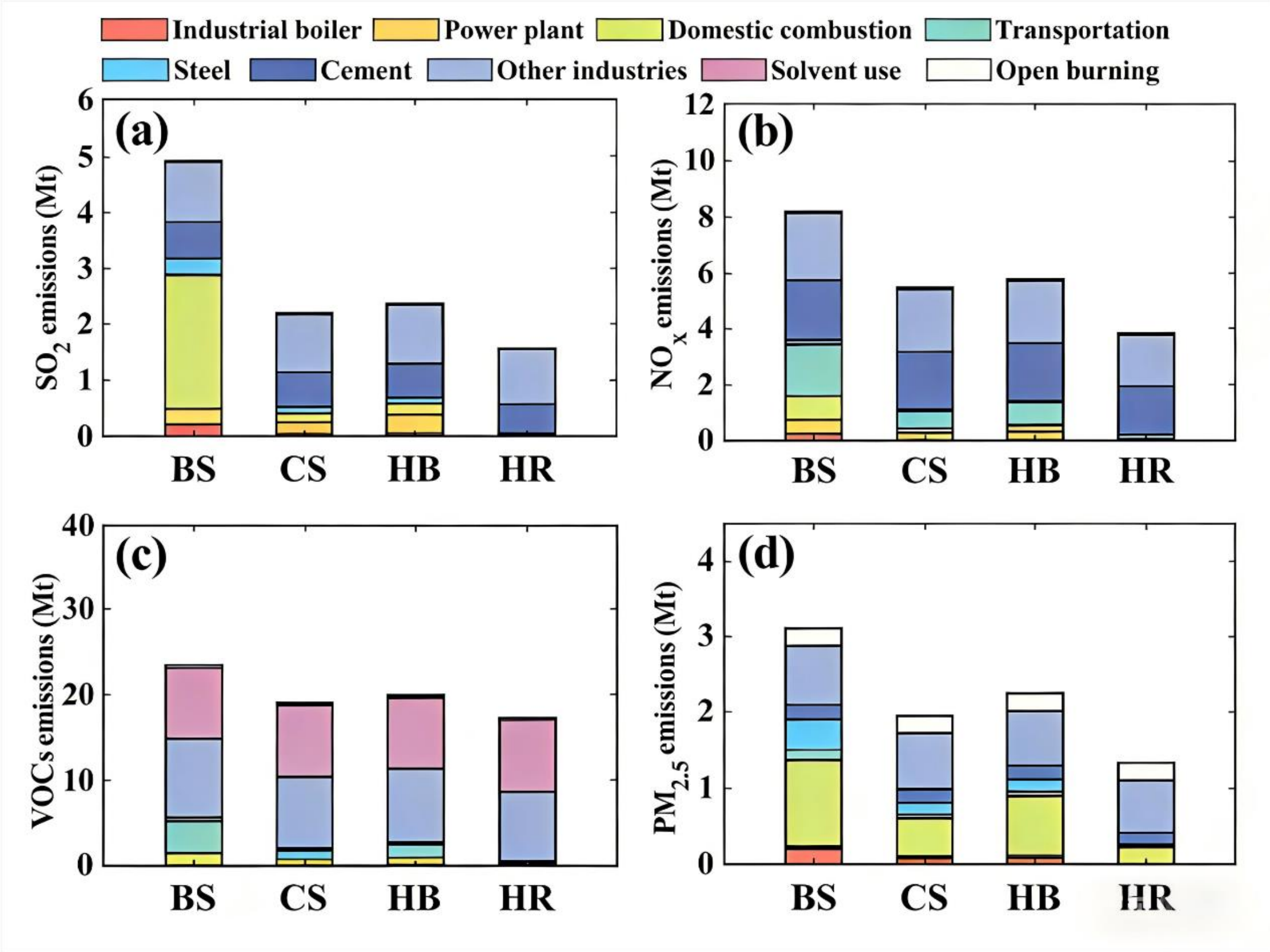

Figure S1. Air pollutant emissions under the baseline scenario (BS) and the three carbon neutrality scenarios (HB, CS, and HR) in 2060. (a) SO_2, (b) NO_x, (c) VOCs, and (d) $PM_{2.5}$ emissions contributed by different sectors under these four scenarios for 2060.

3. Additionally, although the study focuses on China, considering the broader readership of Nature, it would strengthen the paper to reference relevant literature from the Global South—particularly from India, where similar air pollution challenges exist and studies have demonstrated that clean air significantly enhances solar energy production. Incorporating this perspective (e.g., around lines 49–50) would help emphasize the global relevance and broader significance of the work.

R: We thank the reviewer for this helpful suggestion. We agree that highlighting similar challenges in other regions can better emphasize the broader relevance of our study. We have now incorporated relevant literature from India demonstrating that air pollution substantially reduces solar energy production and that cleaner air can significantly enhance photovoltaic generation (Ghosh et al., 2022; Ghosh et al., 2024). These studies illustrate that the interaction between air quality and solar energy is not unique to China but is also a critical issue across the Global South. The corresponding references and discussion have been added in the Introduction

(lines 48-50).

Reference:

1. Ghosh, S., Ganguly, D., Dey, S., & Chowdhury, S. G. (2024). Future photovoltaic potential in India: navigating the interplay between air pollution control and climate change mitigation. *Environmental Research Letters*, *19*(12), 124030.
2. Ghosh, S., Dey, S., Ganguly, D., Baidya Roy, S., & Bali, K. (2022). Cleaner air would enhance India's annual solar energy production by 6–28 TWh. *Environmental Research Letters*, *17*(5), 054007.

4. Line 75–80: The main findings of the study are currently presented in the Introduction. It would be more appropriate to move these details to the Results section and instead include a few concise sentences in the Introduction highlighting the innovation of the paper.

R: We thank the reviewer for this helpful suggestion. We agree that detailed quantitative findings are more appropriately presented in the Results section. Following the reviewer's advice, we have revised the final paragraph of the Introduction by removing the specific numerical results and relocating them to the Results section. The Introduction now focuses on outlining the study framework and highlighting the key innovations of this work, including the integration of energy–economy modeling, high-resolution chemistry–meteorology simulations, and PV performance modeling to quantify pathway-dependent air quality impacts on solar energy.

Introduction (line71-79):

"… By comparing a baseline scenario without carbon neutrality constraints with three alternative carbon neutrality pathways characterized by different technological strategies—high renewables (HR), high biomass (HB), and carbon capture and storage (CS)—this study systematically evaluates how pathway-dependent emission reductions alter aerosol loading, surface solar radiation, and ultimately PV performance. This integrated framework enables a comprehensive assessment of the interactions between decarbonization strategies, air quality improvements, and solar energy resources, providing new insights into how policy-driven technology choices may shape the future performance of PV systems.

"

5. Line 73-80: HR scenario will bring more PV power and that too 2.8 times higher than HB and double to CS. clarify whether this increment comes only from air quality improvement or

from projected PV installation (Table 1) or from both the effect. Authors provide the methodology Line 423 (Estimation of aerosol-induced power generation increase). Also need to look-into the "aerosol-induced" phrase.

R: We thank the reviewer for this important conceptual clarification.

In this study, we define the gains in PV power generation from improved air quality under carbon neutrality pathways as

$$\Delta PV_{POT_{S-BS}} \cdot Capacity_S = \Delta PV_{POT_{S-BS}} \cdot Capacity_{BS} + \Delta PV_{POT_{S-BS}} \cdot \Delta Capacity_{S-BS} \quad (9)$$

where $\Delta PV_{POT_{S-BS}}$ represents the change in PV_{POT} between carbon neutrality pathway S (HB, CS, or HR) and the baseline scenario (BS) due to reduced aerosol loading, and $Capacity_S$ denotes the installed PV capacity under carbon neutrality pathway S. This term therefore represents the PV generation gains attributable to improved air quality under the PV deployment scale of each carbon neutrality pathway.

This total gain consists of two components: a pure resource (air-quality) effect, $\Delta PV_{POT(S-BS)} \cdot Capacity_{BS}$, representing the increase in PV generation due solely to improved PV_{POT} under the baseline installed capacity; and an synergistic effect, $\Delta PV_{POT(S-BS)} \cdot \Delta Capacity_{(S-BS)}$, which reflects the additional gains from improved air quality realized under expanded PV deployment in the carbon neutrality pathways.

To address this concern, we have revised the attribution framework and terminology throughout the manuscript: Results (Line 178-192), Figure 4, and method parts (Line504-516).

Results:

6. Line 143-145: Could the authors clarify why the Xizang and Northwestern regions are expected to show only a minor positive impact of T2 and wind speed (WS) on PV potential? A brief explanation—perhaps linked to regional climatic conditions, topography, or atmospheric circulation patterns—would help readers better understand the underlying physical rationale for these results.

R: We thank the reviewer for this comment. To address it, we first made adjustments to the calculation methodology of the contribution method. In the revised manuscript, we applied a Shapley value – based attribution method to decompose the changes in PV_{POT} into contributions from GHI, T2, and WS. Unlike the previously used one-at-a-time (OAT) approach, the Shapley framework evaluates all factorial combinations of the variables between baseline and scenario conditions, providing a path-independent attribution that accounts for nonlinear responses and

interactions among meteorological drivers.

The updated results confirm that changes in GHI remain the dominant driver of PV_{POT} enhancement across all carbon neutrality pathways. In contrast, the contributions from T2 and WS are substantially smaller. The T2 contribution is consistently negative, reflecting the well-known decrease in PV conversion efficiency with increasing cell temperature. This is because the ground temperature increases projected under all carbon-neutral scenarios relative to the BS is associated with enhanced surface solar radiation, which leads to additional surface heating and, consequently, a further reduction in conversion efficiency.

The WS contribution is even smaller, typically on the order of 10^{-4}, which is two to three orders of magnitude lower than the contribution from GHI. Given this very small magnitude, the WS-induced perturbation has a negligible influence on PV_{POT} changes and does not warrant detailed regional interpretation. To avoid overinterpretation, we have revised the manuscript to emphasize the dominant role of GHI while noting that the contributions of T2 and WS are minor and largely negligible in comparison.

Result part (Line 142-147):

"The contribution of T2 is consistently negative across all regions, reflecting the temperature-induced reduction in photovoltaic conversion efficiency. This negative effect becomes more pronounced under the HR scenario, particularly in the Southern and Central grids. Moreover, the temperature increase in our simulations is partly associated with enhanced surface solar radiation, which leads to additional surface heating. The contribution of WS is two to three orders of magnitude smaller than that of GHI and can therefore be safely neglected in the attribution analysis."

We have also revised the Method part (Line 440-457) and Figure 2.

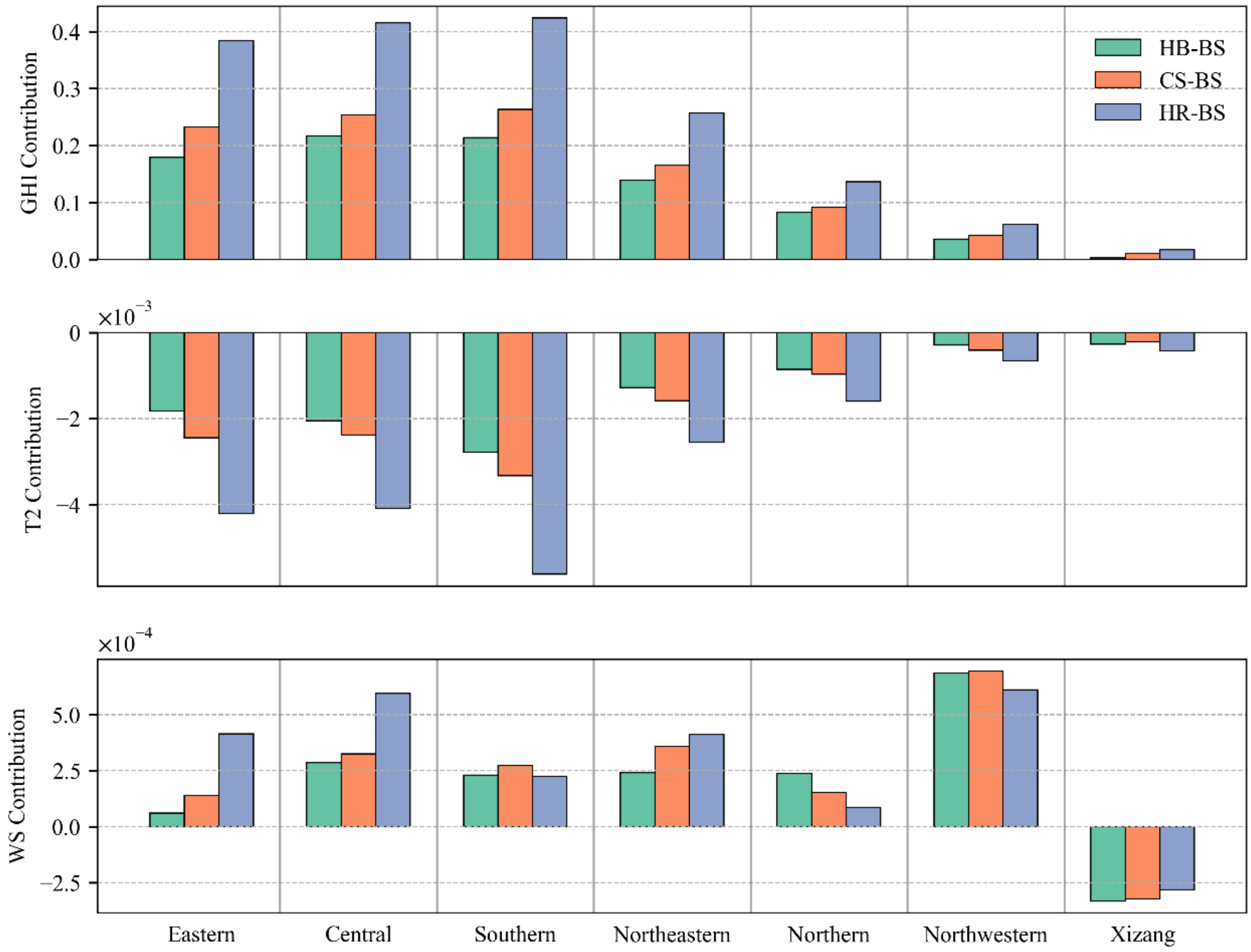

Reference:

1. Prilliman, Matthew J., Clifford W. Hansen, Janine M.F. Keith, Steven Janzou, Marios Theristis, Aaron Scheiner, and Ellis Ozakyol. 2023 (Revised 2024). *Quantifying Uncertainty in PV Energy Estimates Final Report*. Golden, CO: National Renewable Energy Laboratory (NREL). NREL/TP-7A40-84993.

7. I appreciate the authors' effort to connect changes in clear-sky GHI with aerosol-induced cloud impacts and I see authors use the following:

$\Delta C = \Delta(F\_all - F\_clear)$

where F_all is the shortwave radiative flux at the TOA (SFC) under all-sky conditions and F_clear is the flux diagnosed with clouds neglected. Here, Δ: represents the difference between the various experiments (e.g., HR, HB, and CS relative to BS). According to Ghan (2013), Δ typically denotes the difference between atmospheric simulations with and without anthropogenic emissions, while maintaining identical ocean and natural emission conditions.)

Ghan (2013) further proposed that a more realistic attribution involves

$\Delta C\_clean = \Delta(F\_clean - F\_clear,clean)$,

where F_clean is the diagnostic flux neglecting aerosol scattering and absorption, and F_clear,clean is the diagnostic flux neglecting both cloud and aerosol scattering and absorption. Δ: defined already.

The difference between these two approaches— one with all-sky and clear-sky vs. clean-sky and clean-clear-sky diagnostics—was shown to produce a non-negligible signal, particularly for regions such as China and India, where aerosol–cloud interactions are strong. Therefore, the current claim of aerosol-induced cloud impacts on GHI changes may not be fully supported without including clean-sky and clean-clear-sky diagnostics. Incorporating these diagnostics would provide a more robust quantification of aerosol effects, and could potentially alter the number and (interpretations ?) in Lines 177–179, even though signals in LWP and cloud droplet number concentration are evident.

Reference : Ghan (2013) https://acp.copernicus.org/articles/13/9971/2013/

R: We sincerely thank the reviewer for highlighting the attribution framework proposed by Ghan (2013), which provides a rigorous radiative diagnostic approach to separating aerosol direct and indirect effects using clean-sky and clean-clear-sky flux calculations. We agree that the difference between all-sky and clear-sky diagnostics alone does not strictly isolate aerosol–cloud interaction (ACI) effects, and that the clean-sky framework proposed by Ghan (2013) offers a more refined radiative decomposition when attribution relies solely on diagnostic flux outputs.

While we did not initially output the specific F_clean and F_clear,clean diagnostic variables required for Ghan's method, we addressed this attribution challenge using a more physically comprehensive approach: process-level sensitivity experiments (also known as the "switching-on/off" method), which is widely utilized in regional modeling studies (e.g., Gao et al., 2023) to decompose aerosol effects.

Specifically, for each scenario, we conducted two paired perturbation simulations while keeping all other physical and chemical parameterizations unchanged:

1. ACI-off experiment (ARI-on): aerosol–cloud coupling in the cloud microphysics scheme was disabled. The difference between the standard simulation (CTRL) and the ACI-off run therefore quantifies the radiative contribution arising specifically from aerosol–cloud interactions (ΔGHI_ACI).
2. ARI-off experiment (ACI-on): aerosol–radiation coupling in the radiative transfer scheme was suppressed. The difference between CTRL and ARI-off isolates the radiative contribution attributable to aerosol–radiation interactions (ΔGHI_ARI).

Using this method, we find that the dominance of ACI in driving GHI enhancement remains

robust. Across all carbon-neutral scenarios, ΔGHI_ACI is substantially larger than ΔGHI_ARI, and the fractional contribution of ACI increases with emission reductions, reaching its maximum under the most stringent HR pathway. Therefore, the primary conclusion—that indirect aerosol effects via cloud modifications dominate the projected increase in surface solar radiation over China—remains unchanged.

We acknowledge that clean-sky diagnostics can provide additional insight in purely radiative attribution studies. We have updated the Methodology section (Lines 467-490) to formally describe this ARI/ACI decomposition and included references to both Ghan (2013) for the conceptual framework and Gao et al. (2023) for the specific modeling implementation.

Method part (line 459-484):

"Attribution of scenario-induced GHI changes

To isolate the contributions of aerosol–cloud interactions (ACI) and aerosol–radiation interactions (ARI) to scenario-induced changes in surface solar radiation, we performed a set of paired sensitivity experiments. In this study, all baseline and carbon-neutral pathway simulations were conducted using the standard WRF-Chem configuration, in which both ARI and ACI are enabled. For clarity, we refer to this standard configuration as the CTRL simulation. In addition to CTRL runs, two sensitivity experiments were conducted for each scenario, adopting the process-level separation approach detailed by Gao et al. (2023): (1) ACI-off experiment (ARI-on), in which aerosol–cloud coupling in cloud microphysics was disabled. The difference between CTRL and ACI-off therefore represents the radiative contribution arising specifically from aerosol–cloud interactions ($\Delta\mathrm{GHI}_{ACI}$), representing an alternative method to isolate cloud-aerosol forcing as proposed by Ghan (2013). (2) ARI-off experiment (ACI-on), in which aerosol–radiation coupling in the radiative transfer scheme was suppressed. The difference between CTRL and ARI-off thus isolates the radiative contribution attributable to aerosol–radiation interactions ($\Delta\mathrm{GHI}_{ARI}$). Under this differential framework, the total scenario-induced change in GHI can be approximately decomposed into ACI and ARI contributions.

Our attribution focuses on scenario-induced increments relative to the baseline (BS) scenario, rather than absolute aerosol effects. For each future carbon-neutral scenario X (HR, HB, and CS), the total change in surface global horizontal irradiance (GHI) relative to BS is defined as

$$\Delta\mathrm{GHI} = \mathrm{GHI}_{\mathrm{CTRL}}^{\mathrm{X}} - \mathrm{GHI}_{\mathrm{CTRL}}^{\mathrm{BS}}$$

The contribution from aerosol–cloud interactions (ACI) is diagnosed as

$$\Delta\mathrm{GHI}_{ACI} = \left(\mathrm{GHI}_{\mathrm{CTRL}}^{\mathrm{X}} - \mathrm{GHI}_{\mathrm{ACloff}}^{\mathrm{X}}\right) - \left(\mathrm{GHI}_{\mathrm{CTRL}}^{\mathrm{BS}} - \mathrm{GHI}_{\mathrm{ACloff}}^{\mathrm{BS}}\right)$$

, which represents the enhancement of GHI attributable to ACI under scenario X relative to BS.

Similarly, the contribution from aerosol–radiation interactions (ARI) is defined as

$$\Delta \mathrm{GHI}_{ARI} = \left(\mathrm{GHI}_{\mathrm{CTRL}}^{\mathrm{X}} - \mathrm{GHI}_{\mathrm{ARIoff}}^{\mathrm{X}}\right) - \left(\mathrm{GHI}_{\mathrm{CTRL}}^{\mathrm{BS}} - \mathrm{GHI}_{\mathrm{ARIoff}}^{\mathrm{BS}}\right)$$

, which represents the enhancement of GHI attributable to ARI under scenario X relative to BS.

"

8. Line 183-186: The assumption of an average of 1,500 operational hours should be supported with a relevant reference or data source, as this parameter directly influences subsequent estimates of PV generation and associated economic benefits. Providing a citation—ideally from national energy statistics, technical reports, peer-reviewed studies or your rationale—would strengthen the credibility and practical basis of this assumption.

R: We thank the reviewer for this helpful comment. In the original manuscript, the description of PV generation calculation was not sufficiently clear. In the revised manuscript, we have corrected and clarified this calculation.

In this study, PV power generation is derived from hourly simulations of PV_{POT}. PV_{POT} represents the ratio between the actual PV power output and the rated capacity under given environmental conditions. Hourly PV_{POT} values were calculated using the pvlib model driven by simulated meteorological variables and then averaged over the year to obtain the annual mean PV_{POT}. Thus, the annual PV power generation is calculated as the product of the annual mean PV_{POT}, the installed PV capacity, and the total number of hours in a year (8760 h) (Bolson et al., 2022).

The corresponding description has been clarified in the revised manuscript (Method part, Line 523-528). And the Figure 4 has been updated.

"

…Annual PV power generation is calculated as the product of the annual mean PV_{POT}, the installed PV capacity, and the total number of hours in a year (8760 h). The annual mean PV_{POT} is derived from hourly simulations using the pvlib model driven by meteorological variables and represents the capacity factor of the PV system. The increase in PV generation under different scenarios is then multiplied by the assumed electricity price to estimate the corresponding economic benefits of PV power generation under different scenarios in each region.

"

Reference:

1. Bolson, N., Prieto, P., & Patzek, T. (2022). Capacity factors for electrical power generation from renewable and nonrenewable sources. *Proceedings of the National Academy of Sciences*, *119*(52), e2205429119.

9. Line 185–187: "Multiplied the aerosol-induced PV power increase by 1,500 hours to obtain the annual power generation. Figure 4a illustrates the aerosol-induced PV power generation…" The claim of aerosol-induced is not appropriate, because the authors use various emission-driven scenarios that obviously improve air quality but also bring changes in cloud properties (COD, Fig. 3, and also Supplementary). Therefore, the aerosol emission reductions in HR, HB, and CS pathways change GHI and PV potential by modulating aerosols, clouds, and other meteorological parameters. Hence, claiming only aerosol-induced PV potential change is not conclusive. For that, the authors need additional diagnostics stated in Comment 6, or they could focus only on how much change they observe in GHI_clear (which mainly depends on aerosols). A better phrase could be "aerosol-emission reduction pathways driven" / "carbon-neutrality pathways driven" (which are stated in Line 202). Change in phrase is also necessary in Figure 4 also.

R: We thank the reviewer for this important conceptual clarification.

In this study, we define the gains in PV power generation from improved air quality under carbon neutrality pathways as

$$\Delta PV_{POT\,S-BS} \cdot Capacity_S = \Delta PV_{POT\,S-BS} \cdot Capacity_{BS} + \Delta PV_{POT\,S-BS} \cdot \Delta Capacity_{S-BS} \quad (9)$$

where $\Delta PV_{POT\,S-BS}$ represents the change in PV_{POT} between carbon neutrality pathway S (HB, CS, or HR) and the baseline scenario (BS) due to reduced aerosol loading, and $Capacity_S$ denotes the installed PV capacity under carbon neutrality pathway S. This term therefore represents the PV generation gains attributable to improved air quality under the PV deployment scale of each carbon neutrality pathway.

This total gain consists of two components: a pure resource (air-quality) effect, $\Delta PV_{POT(S-BS)} \cdot Capacity_{BS}$, representing the increase in PV generation due solely to improved PV_{POT} under the baseline installed capacity; and an synergistic effect, $\Delta PV_{POT(S-BS)} \cdot \Delta Capacity_{(S\_BS)}$, which reflects the additional gains from improved air quality realized under expanded PV deployment in the carbon neutrality pathways.

To address this concern, we have revised the attribution framework and terminology throughout the manuscript: Results (Line 178-192), Figure 4, and method parts (Line504-516).

10. Line 353: How sensitive is the pvlib package when decomposing direct and diffuse radiation using only solar position? How confident are the authors about the diffuse component, which is more sensitive to aerosol type and cloud properties, whereas the direct component is comparatively less sensitive to these factors? This decomposition is further utilized in the POAI calculation (Lines 370–371).

R: We appreciate the reviewer's professional insight into the radiation decomposition process. We would like to clarify the methodology used and address the concerns regarding the sensitivity of the diffuse component.

We would like to clarify that the decomposition was not based solely on solar position. We utilized the simulated GHI from WRF-Chem as the core physical constraint. For the decomposition of GHI into Direct Normal Irradiance (DNI) and Diffuse Horizontal Irradiance (DHI), we employed the DISC model via the pvlib package. The DISC model is a widely recognized quasi-physical algorithm utilized by the National Solar Radiation Database (NSRDB). It is considered one of the most reliable decomposition models because it empirically relates the clearness index to the direct component, providing a robust partition even under complex sky conditions.

As noted by Mayer and Yang (2023), the choice of physical model chains—including separation (decomposition), transposition, and temperature models — can introduce uncertainties exceeding 10% in power output simulations. To mitigate this: We selected the DISC model specifically for its proven performance in large-scale solar resource assessments.

Furthermore, the related code has been deposited in a public repository, and we have added the description in the Methods section (line 399-405).

"…1) Radiation Decomposition: decomposing GHI into direct and diffuse components using the DISC model, a quasi-physical algorithm widely adopted by the National Solar Radiation Database (NSRDB) for its robust performance in partitioning radiation based on the clearness index and solar position, 2) Transposition and Optical Losses, utilizing the Perez model to calculate Plane-of-Array (POA) irradiance, while incorporating the Xie model for reflection losses and SAPM for spectral correction; and 3) Power Output Calculation, where cell temperature was estimated via the SAPM temperature model, and final DC power was determined using the PVWatts model…"

Reference:

1. Mayer, M. J., & Yang, D. (2023). Pairing ensemble numerical weather prediction with ensemble physical model chain for probabilistic photovoltaic power

forecasting. *Renewable and Sustainable Energy Reviews*, *175*, 113171.

11. Line 383: Contribution of GHI, T, and WS on PVPOT: This section contains descriptive text. The authors should refer to the existing literature where a detailed mathematical formulation of these contributions has already been presented.

R: We thank the reviewer for this constructive suggestion. To address the nonlinear interactions between these variables, we have moved beyond simple descriptive analysis and implemented the Shapley Value decomposition method. This approach, widely used in cooperative game theory and recently adapted for climate and energy attribution, allows for a path-independent and fair allocation of contributions from each driver.

We have updated the Methods section (line 439-456) accordingly and revised Fig. 2 to reflect the Shapley-based attribution results.

"

Contribution of GHI, T and WS on PV_{POT}

PV output responds nonlinearly to GHI, T2, and WS (Yang et al., 2022), traditional one-at-a-time approaches cannot provide a path-independent attribution of their contributions (Ndiaye et al., 2025; He and Zhao, 2025). Thus, to quantitatively analyze the contribution of GHI, T2, and WS to changes in PV_{POT}, a Shapley value–based decomposition method is employed in this study (Shapley, 1953). PV_{POT} is a nonlinear function of meteorological drivers,

$$PV_{POT} = f(GHI, T2, WS)$$

where f represents the full pvlib-based photovoltaic performance model chain.

For a specific driver $i \in N$ (here, $N = \{GHI, T2, WS\}$), its Shapley value ϕ_i representing its average marginal contribution to the total PV_{POT} change (ΔPV_{POT}), is defined as

$$\phi_i = \sum_{S \subseteq N \setminus \{i\}} \frac{|S|!\,(|N| - |S| - 1)!}{|N|!} [f(S \cup \{i\}) - f(S)]$$

where S denotes any subset of variables excluding i, and $|N| = 3$ in this study. This framework ensures additive closure, such that:

$$\Delta PV_{POT} = \phi_{GHI} + \phi_{T_2} + \phi_{WS}$$

For each carbon-neutrality pathway (HB, CS, HR), all $2^{|N|} = 8$ factorial combinations of baseline and scenario meteorological variables were constructed. PV_{POT} was evaluated for each combination using the pvlib model chain, and Shapley contributions were computed grid-point-

wise through explicit enumeration of all marginal contributions.

”

Reference:

1. Shapley, L. S. (1953). A value for n-person games. Contributions to the Theory of Games, 2(28), 307-317.
2. Yang, D., Wang, W., Gueymard, C. A., Hong, T., Kleissl, J., Huang, J., ... & Peters, I. M. (2022). A review of solar forecasting, its dependence on atmospheric sciences and implications for grid integration: Towards carbon neutrality. Renewable and Sustainable Energy Reviews, 161, 112348.
3. Ndiaye, A., Faye, M., Gueye, D., Ba, A., & Traore, M. (2025). Analysis of the impact of irradiance and temperature on photovoltaic production: A statistical and machine learning approach. MethodsX, 103716.
4. He, B., & Zhao, C. (2025). A novel method to quantify the uncertainty contribution of aerosol–radiation interaction factors. *Atmospheric Chemistry and Physics*, *25*(14), 7765-7776.

Methods:

12. A map showing 7 power grids in the supplementary is encouraged.

R: We thank the reviewer for this helpful suggestion. We have added a new map illustrating China’s inter-provincial regional power grids as Supplementary Figure S6. This figure provides the spatial framework for all regional aggregations of PVPOT, aerosol-induced PV generation changes, and projected PV capacity discussed in the manuscript. We have also added a corresponding reference to this figure in the main text (Results section).

Supplementary part:

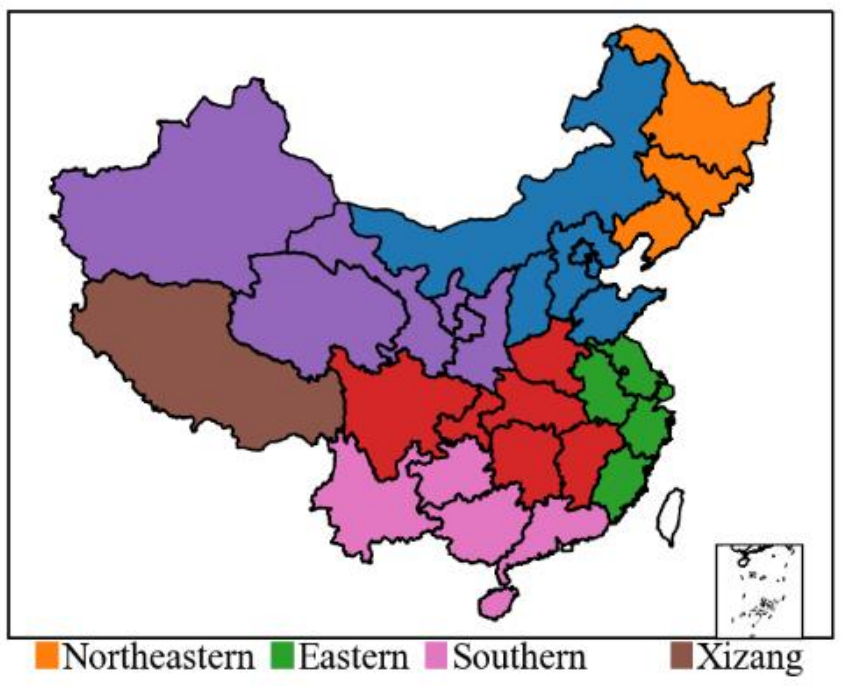

Figure S6. China’s inter-provincial regional power grids.

Results/ Methods part:

“In this study, we analyze the results based on China's seven regional power grids (Supplementary Fig. S6): Eastern, Central, Southern, Northeastern, Northern, Northwestern, and Xizang (Zhuo et al., 2022).”

Reference：

1. Zhuo, Z., Du, E., Zhang, N., Nielsen, C. P., Lu, X., Xiao, J., ... & Kang, C. (2022). Cost increase in the electricity supply to achieve carbon neutrality in China. Nature communications, 13(1), 3172.

13. What is the horizontal resolution of the WRF simulation? Although the authors have used a previously published WRF setup, providing a few key details of the model configuration (e.g., domain size, resolution, nesting strategy, and physics schemes) would make the current paper more self-contained and comprehensible as a standalone read.

R: We thank the reviewer for this helpful suggestion. We agree with the reviewer that providing key model configuration details enhances the self-containment of the manuscript. Accordingly, we have added a brief description of the WRF-Chem configuration in the Methods and Supplementary.

The WRF-Chem simulation is conducted using a single domain covering the entire land area of China with a horizontal grid spacing of 27 km. The model uses 24 vertical layers extending from the surface to 50 hPa, with enhanced resolution within the planetary boundary layer (PBL). Aerosol direct radiative effects as well as the first and second aerosol indirect effects on grid-scale clouds are included.

The major physical parameterizations include the Morrison double-moment microphysics scheme (Morrison et al., 2009), the RRTMG shortwave and longwave radiation schemes (Iacono et al., 2008), the Eta similarity surface layer scheme (Janjic et al., 1994), the Noah land surface model (Niu et al., 2011), the Bougeault–Lacarrère PBL scheme (Bougeault and Lacarrère, 1989), and the Grell–Freitas ensemble cumulus scheme (Grell and Freitas, 2014). Biogenic emissions are calculated online using MEGAN v2.04, while dust emissions are represented using the GOCART scheme.

For atmospheric chemistry, we employ the SAPRC-99 gas-phase mechanism (Carter, 2000) coupled with the MOSAIC aerosol module (Zaveri et al., 2008), which represents aerosol size distributions using eight size bins and includes major aerosol processes. Secondary organic aerosol formation is further represented using the volatility basis set (VBS) framework (Shrivastava et al., 2011). Photolysis rates are calculated using the Fast-J photolysis scheme (Wild et al., 2000).

Accordingly, we have added a brief description of the WRF-Chem configuration in the Methods section (line 334-340).

"…

The WRF-Chem simulation is conducted using a single domain covering the entire land area of China with a horizontal grid spacing of 27 km. The model uses 24 vertical layers extending from the surface to 50 hPa, with enhanced resolution within the planetary boundary layer (PBL). Aerosol direct radiative effects as well as the first and second aerosol indirect effects on grid-scale clouds are included. The model configuration, including physical and chemical parameterizations, follows the framework established in Gao, et al. [32,33], with detailed settings summarized in Table S3…

"

Table S2 The configuration of WRF-Chem in this study.

**Configuration**	**Description**
Horizontal Resolution	27 km × 27 km
Grid Dimensions	182 (West-East) × 232 (South-North)
Vertical Layers	24 layers (top at 50 hPa)
Radiation Scheme	RRTMG (Shortwave and Longwave)
Microphysics	Morrison 2-moment scheme
Surface layer scheme	Eta similarity
Cumulus Scheme	Grell-3
PBL scheme	Bougeault–Lacarrère

Land Surface	Noah Land Surface Model
Biogenic emissions	MEGAN v2.04
Dust emissions	GOCART
Photolysis rates	Fast-J photolysis scheme
Gas-phase Mechanism	CBMZ
Aerosol Module	MOSAIC (8-bin)
Secondary organic aerosol	volatility basis set (VBS) framework

”

14. Why only SSP126 climate data used for future scenarios? (Line 337) Provide your rationale why other SSPs will not qualify towards the Carbon neutrality target for China.

R: We thank the reviewer for this important question. SSP126 was selected as the climate forcing scenario primarily to ensure consistency with the global low-emission background required for achieving carbon neutrality.

First, regarding meteorological forcing, future meteorological initial and boundary conditions were derived from a bias-corrected ensemble mean of 18 CMIP6 global climate models under SSP126 (Xu et al., 2021). Using an ensemble mean reduces uncertainties associated with individual model climate sensitivity and large-scale circulation biases, thereby providing a more robust representation of future meteorological conditions. Consequently, the dynamical background driving the WRF-Chem simulations already incorporates inter-model spread at the climate level.

Second, SSP126 represents a globally consistent low-carbon development pathway in which CO_2 emissions decline rapidly and approach net-zero around the middle of the century (Riahi et al., 2017). This trajectory closely aligns with the emission reductions implied by China’s carbon-neutral pathways used in this study. As illustrated in the revised manuscript (as following figure), the cumulative CO_2 emissions for China under SSP126 are broadly consistent with those in our national carbon-neutral scenarios. Therefore, using SSP126 provides a coherent global climate background for evaluating the impacts of China’s domestic emission mitigation pathways.

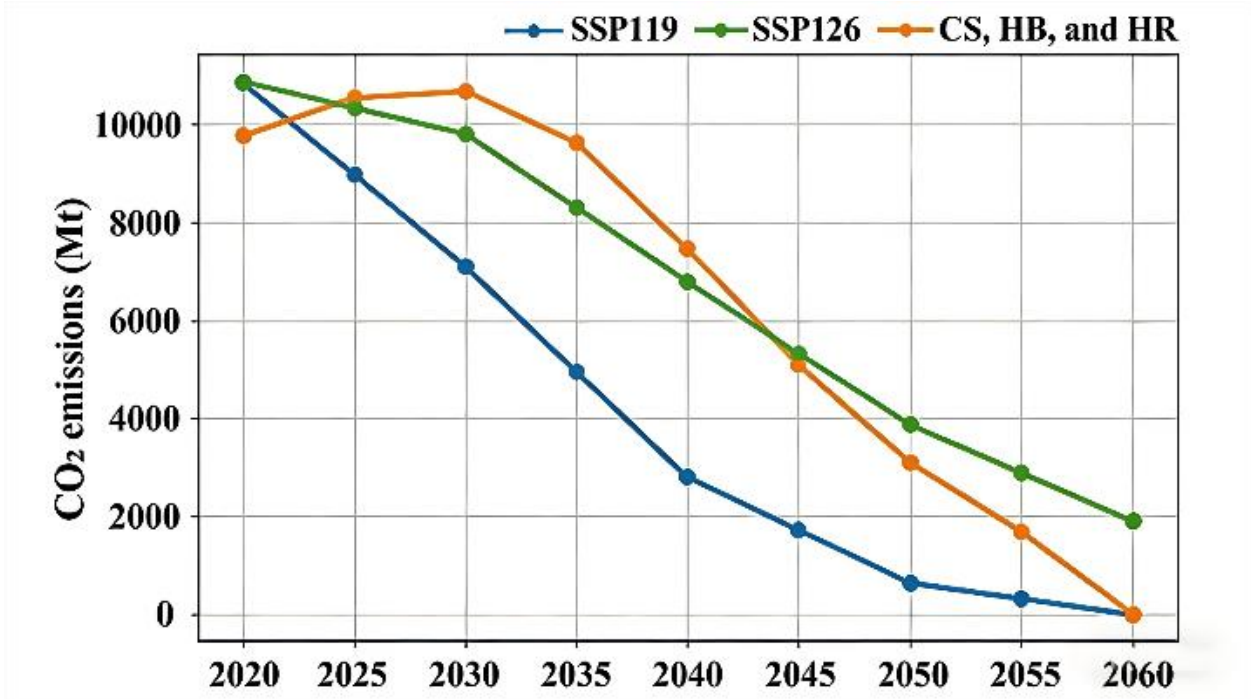

Figure. Emission of CO_2 in China under SSP119, SSP126, and the three carbon neutrality 310 scenarios (HB, CS, and HR).

For chemical initial and boundary conditions, future concentrations were constructed by scaling present-day global chemical fields using SSP126-based concentration ratios for 2021–2060. A proportional scaling approach was adopted because CMIP6 chemistry outputs generally employ simplified chemical mechanisms and may incompletely represent certain $PM_{2.5}$ components (particularly organic aerosols) (Emmons et al., 2020; Sellar et al., 2020; Wu et al., 2020). This ratio-based method preserves the large-scale spatial structure of chemical fields while ensuring consistency with the SSP126 global forcing trajectory.

We have added the information in the revised method Part: Line 351-367.

Reference

1. Cheng, J.; Tong, D.; Zhang, Q.; Liu, Y.; Lei, Y.; Yan, G.; Yan, L.; Yu, S.; Cui, R. Y.; Clarke, L.; et al. Pathways of China's PM2.5 air quality 2015-2060 in the context of carbon neutrality. Natl Sci Rev 2021, 8 (12). DOI: 10.1093/nsr/nwab078.

2. Sun, Y. S.; Jiang, Y. Q.; Xing, J.; Ou, Y.; Wang, S. X.; Loughlin, D. H.; Yu, S.; Ren, L.; Li, S. Y.; Dong, Z. X.; et al. Air Quality, Health, and Equity Benefits of Carbon Neutrality and Clean Air Pathways in China. Environ Sci Technol 2024, 58 (34), 15027-15037. DOI: 10.1021/acs.est.3c10076.

3. Zhang, S. H.; An, K. X.; Li, J.; Weng, Y. W.; Zhang, S. H.; Wang, S. X.; Cai, W. J.; Wang, C.; Gong, P. Incorporating health co-benefits into technology pathways to achieve China's 2060 carbon neutrality goal: a modelling study. Lancet Planet Health 2021, 5 (11), E808-E817. DOI: 10.1016/S2542-5196(21)00252-7.

4. Xu, Z. F., Han, Y., Tam, C. Y., Yang, Z. L. & Fu, C. B. Bias-corrected CMIP6 global dataset for dynamical downscaling of the historical and future climate (1979-2100). Sci Data 8, doi:10.1038/s41597-021-01079-3 (2021).

15. It is observed that, both AOD and COD decreases in all scenarios w.r.t Baseline scenario. Are AOD and COD enough to establish the aerosol-cloud interaction?

R: We thank the reviewer for this insightful comment. To better isolate the physical mechanisms linking aerosol reductions to changes in surface radiation, we have conducted additional sensitivity experiments that separately diagnose the roles of aerosol–cloud interactions (ACI) and aerosol–radiation interactions (ARI).

Specifically, two sensitivity experiments were performed for each scenario:
(1) an ACI-off experiment (ARI-on), in which aerosol–cloud coupling in the cloud microphysics scheme was disabled; (2) an ARI-off experiment (ACI-on), in which aerosol–radiation coupling in the radiative transfer scheme was suppressed.

The standard WRF-Chem configuration used throughout the manuscript (with both ACI and ARI enabled) is referred to as the CTRL simulation. Differences between CTRL and the ACI-off experiment quantify the radiative contribution from aerosol–cloud interactions (ΔGHI_ACI), while differences between CTRL and the ARI-off experiment isolate the contribution from aerosol–radiation interactions (ΔGHI_ARI). Additional details of these experiments are provided in our response to Comment 7.

The revised analysis shows that our main conclusions remain unchanged. Although ΔGHI_ARI exhibits a modest positive contribution (domain means of 0.37, 0.47, and 0.69 W m^{-2} for HB, CS, and HR, respectively), the ΔGHI_ACI component is substantially larger (1.32, 1.64, and 2.66 W m^{-2}). Overall, ACI accounts for roughly three-quarters of the total radiation enhancement, and its fractional contribution increases from HB to CS and reaches a maximum under the most stringent HR pathway. These results confirm that the simulated increases in surface solar radiation are primarily driven by aerosol–cloud interactions rather than aerosol–radiation effects.

These results have been added to the revised manuscript. Method (line 458-482), Result (line 156-167) and Fig. 3

16. It is observed that the authors provide uncertainty quantification primarily associated with the WRF simulations. However, additional uncertainties may also arise from the emission trajectory models, pvlib-based calculations, the economic assessment, and several underlying assumptions. It is therefore encouraged to include a brief discussion in the Methods or Supplementary Information to illustrate and acknowledge these sources of uncertainty. This addition would also enhance the policy relevance of the study, and the authors are encouraged

to comment on its global applicability and reproducibility for other regions with similar contexts or interests.

R: We thank the reviewer for this insightful comment. We agree that uncertainties in this integrated modeling framework may arise not only from the WRF-Chem simulations but also from other components, including emission trajectories, PV performance modeling, economic valuation, and several scenario assumptions.

In PV energy estimates, solar radiation (GHI) uncertainty is regarded as one of the most critical and largest sources of uncertainty (Prilliman et al., 2023). Therefore, in this study we explicitly quantified this component using the EasyUQ probabilistic framework and propagated it through the PV modeling chain.

We have added a brief discussion on this point in Supplementary Text S2.

"

Text S2. Sources of uncertainty and limitations

To quantify the uncertainty associated with projected PV_{POT}, PV power generation and economic benefits increase under different carbon neutrality pathways relative to the baseline, uncertainties across the entire integrated modeling framework should ideally be considered, including energy–economy scenarios, emission inventories, atmospheric chemistry–meteorology simulations, PV performance models, and economic valuation. In principle, a fully integrated Monte Carlo or multi-model ensemble propagating uncertainties across all components would provide a comprehensive assessment; however, such an end-to-end uncertainty propagation is computationally prohibitive in practice.

In this study, we therefore focus on explicitly quantifying the uncertainty associated with simulated solar radiation (GHI) from WRF-Chem, which represents the dominant driver of variability in PV performance (Prilliman et al., 2023). These uncertainties are quantified using the EasyUQ framework and propagated through the pvlib-based PV modeling chain to estimate the resulting variability in PV_{POT}, PV power generation and economic benefits increase.

Additional sources of uncertainty may arise from emission trajectories derived from the C-GEM energy–economy model, assumptions about future technology deployment and emission factors, scheme uncertainties in atmospheric chemistry–meteorology simulations, parameter assumptions in the pvlib-based PV performance modeling, and the electricity price used in the economic valuation. While these factors may influence the absolute magnitude of projected PV benefits, the primary objective of this study is to compare relative differences among carbon neutrality pathways. Because the same modeling framework and parameter assumptions are consistently applied across scenarios, many systematic biases are expected to be minimized when evaluating pathway differences. Furthermore, the integrated framework

developed here is transferable to other regions, provided that region-specific emission scenarios, meteorological projections, and PV deployment pathways are available.
”

Reference:

1. Prilliman, Matthew J., Clifford W. Hansen, Janine M.F. Keith, Steven Janzou, Marios Theristis, Aaron Scheiner, and Ellis Ozakyol. 2023 (Revised 2024). *Quantifying Uncertainty in PV Energy Estimates Final Report*. Golden, CO: National Renewable Energy Laboratory (NREL). NREL/TP-7A40-84993.

17. (Minor) Provide units in the table in the supplementary.
R: Done.

Reviewer #2 (Remarks on code availability):

Looks okay.

R: We fully agree with the reviewer that transparency and reproducibility are essential for scientific advancement. In response, we have archived all the necessary resources and source code used in this study to a public repository (available at: https://github.com/shihrong/PV-China-2060), including emission data, namelist, pvlib code, visualization code, and so on.

Reviewer #3 (Remarks to the Author):

I co-reviewed this manuscript with one of the reviewers who provided the listed reports. This is part of the Nature Communications initiative to facilitate training in peer review and to provide appropriate recognition for Early Career Researchers who co-review manuscripts.

R: Thank you for your co-review and valuable comments on this manuscript.

Reviewer #4 (Remarks to the Author):

The paper presents very interesting results, showing for the first time a quantitative economic assessment of the impact of different carbon neutrality pathways on PV production in China. Unlike most previous works that evaluate solar potential, in this case, the climate and energy models are coupled in a modeling chain. The outputs of changes in energy production are then evaluated and, going a step further, these are expressed in economic terms, although through a relatively simple conversion. One of the interesting aspects is that the authors put the results into context to help understand the magnitude of the economic changes. This work allows for a comparison of the impacts of different carbon neutrality strategies, which is important given the dependence of photovoltaic production on air quality.

I found a weakness in the fact that only one climate model was used for the simulation of the meteorological variables, which limits the robustness of the results presented. Part of the uncertainty arises from the model itself; therefore, using an ensemble of models would enhance the reliability of the results. In addition, a three-year simulation period is too short to capture variability properly. Longer simulations are needed to obtain representative mean values. I assume the authors tried to address this limitation using the probabilistic approach explained at the end of the paper — namely, EasyUQ as a post-processing technique. However, the paper should be revised on this point. An evaluation of the accuracy of this statistical approach should be provided, and the limitations related to the uncertainty of using a single climate model and a short simulation period must be clearly stated in the text.

R: We thank the reviewer for raising these important points regarding the robustness of the meteorological forcing and the treatment of uncertainty. We address the concerns regarding (1) the use of climate models, (2) the length of the simulation period, and (3) the probabilistic uncertainty analysis as follows.

(1) Although the regional simulations were conducted with WRF-Chem, the meteorological initial and boundary conditions for the future period were derived from the bias-corrected ensemble mean of 18 CMIP6 global climate models under the SSP126 scenario (Xu et al., 2021). This multi-model ensemble forcing provides two key advantages. First, it reduces

uncertainty associated with the climate sensitivity and circulation biases of individual models. Second, the bias correction against reanalysis data improves the realism of the large-scale meteorological fields used to drive the regional simulations. Therefore, the dynamical background driving WRF-Chem already incorporates the spread across multiple climate models, rather than relying on a single GCM.

(2) We performed simulations for three years (2059–2061) to reduce the influence of internal climate variability on projected $PM_{2.5}$ concentrations and radiation fields. While a longer simulation period could further average out natural variability, the use of ensemble-derived meteorological forcing from 18 climate models already mitigates low-frequency variability to a large extent. Moreover, this approach is consistent with many recent policy-relevant studies that assess future air quality and climate co-benefits using ensemble climate forcing combined with limited simulation periods (e.g., Zhang et al., 2021; Cheng et al., 2021; Sun et al., 2024).

Above all, while this timeframe may not capture decadal climatological shifts, it provides a robust basis for isolating the radiative benefits of air quality improvements under diverse carbon-neutrality strategies. Our analysis focuses on relative differences across emission scenarios rather than absolute meteorological or climate states, so the influence of interannual meteorological variability on our key conclusions is greatly minimized.

(3) In PV energy estimates, solar radiation uncertainty is regarded as one of the most critical and largest sources of uncertainty (Prilliman et al., 2023). Among all components of the PV model chain, surface solar radiation (GHI) overwhelmingly dominates variability in PV potential, whereas uncertainties associated with pvlib model options, temperature coefficients, or wind-speed parameterizations are well documented to be second order relative to irradiance uncertainty. We therefore focus our quantitative uncertainty analysis on GHI.

Given these constraints, to quantify the GHI uncertainty associated with deterministic WRF-Chem simulations, we employed the EasyUQ framework, which transforms deterministic WRF-Chem radiation outputs into calibrated, probabilistic GHI distributions. By leveraging a training archive of model output–outcome pairs (WRF-Chem outputs and ground-based observations), EasyUQ generates probabilistic GHI forecasts that reflect the inherent uncertainty in the simulation's historical performance (Walz et al., 2024).

Standard deviations for individual scenario were first back-calculated from the EasyUQ-calibrated 68% confidence intervals. To quantify the uncertainty of PV_{POT} changes under carbon neutrality scenarios relative to the BS, we employed the law of error propagation. The ±1σ for each scenario contrast was calculated as the square root of the sum of the variances (Var(scenario-BS) = Var(scenario) + Var(BS)), assuming stochastic independence

between the emission-driven pathways. This approach provides a robust measure of the statistical significance of air-quality-induced PV gains relative to GHI uncertainties.

Following the reviewer's suggestion, we have revised the manuscript in **Method part (line 351-376 and 530-551), and supplementary Text S2.**

"

Text S2. Sources of uncertainty and limitations

To quantify the uncertainty associated with projected PV_{POT}, PV power generation and economic benefits increase under different carbon neutrality pathways relative to the baseline, uncertainties across the entire integrated modeling framework should ideally be considered, including energy–economy scenarios, emission inventories, atmospheric chemistry–meteorology simulations, PV performance models, and economic valuation. In principle, a fully integrated Monte Carlo or multi-model ensemble propagating uncertainties across all components would provide a comprehensive assessment; however, such an end-to-end uncertainty propagation is computationally prohibitive in practice.

In this study, we therefore focus on explicitly quantifying the uncertainty associated with simulated solar radiation (GHI) from WRF-Chem, which represents the dominant driver of variability in PV performance (Prilliman et al., 2023). These uncertainties are quantified using the EasyUQ framework and propagated through the pvlib-based PV modeling chain to estimate the resulting variability in PV_{POT}, PV power generation and economic benefits increase.

Additional sources of uncertainty may arise from emission trajectories derived from the C-GEM energy–economy model, assumptions about future technology deployment and emission factors, scheme uncertainties in atmospheric chemistry–meteorology simulations, parameter assumptions in the pvlib-based PV performance modeling, and the electricity price used in the economic valuation. While these factors may influence the absolute magnitude of projected PV benefits, the primary objective of this study is to compare relative differences among carbon neutrality pathways. Because the same modeling framework and parameter assumptions are consistently applied across scenarios, many systematic biases are expected to be minimized when evaluating pathway differences. Furthermore, the integrated framework developed here is transferable to other regions, provided that region-specific emission scenarios, meteorological projections, and PV deployment pathways are available.

"

Reference:

1. Prilliman, Matthew J., Clifford W. Hansen, Janine M.F. Keith, Steven Janzou, Marios Theristis, Aaron Scheiner, and Ellis Ozakyol. 2023 (Revised 2024). *Quantifying*

Uncertainty in PV Energy Estimates Final Report. Golden, CO: National Renewable Energy Laboratory (NREL). NREL/TP-7A40-84993.

2. Walz, E.-M., Henzi, A., Ziegel, J. & Gneiting, T. Easy Uncertainty Quantification (EasyUQ): Generating predictive distributions from single-valued model output. *Siam Review* **66**, 91-122 (2024)
3. Cheng, J.; Tong, D.; Zhang, Q.; Liu, Y.; Lei, Y.; Yan, G.; Yan, L.; Yu, S.; Cui, R. Y.; Clarke, L.; et al. Pathways of China's PM2.5 air quality 2015-2060 in the context of carbon neutrality. Natl Sci Rev 2021, 8 (12). DOI: 10.1093/nsr/nwab078.
4. Sun, Y. S.; Jiang, Y. Q.; Xing, J.; Ou, Y.; Wang, S. X.; Loughlin, D. H.; Yu, S.; Ren, L.; Li, S. Y.; Dong, Z. X.; et al. Air Quality, Health, and Equity Benefits of Carbon Neutrality and Clean Air Pathways in China. Environ Sci Technol 2024, 58 (34), 15027-15037. DOI: 10.1021/acs.est.3c10076.
5. Zhang, S. H.; An, K. X.; Li, J.; Weng, Y. W.; Zhang, S. H.; Wang, S. X.; Cai, W. J.; Wang, C.; Gong, P. Incorporating health co-benefits into technology pathways to achieve China's 2060 carbon neutrality goal: a modelling study. Lancet Planet Health 2021, 5 (11), E808-E817. DOI: 10.1016/S2542-5196(21)00252-7.

Another relevant point is that solar radiation (GHI) was evaluated against ground stations but only for one year. A longer validation period is required to assess the model’s performance across different time scales.

R: We thank the reviewer for this helpful comment. We agree that evaluating model performance over multiple years is desirable for assessing robustness across different temporal scales.

While the GHI validation in the current manuscript focuses on one year, the WRF-Chem configuration used in this study has been extensively validated over a much longer period in our previous work (Gao et al., 2023). In Gao et al. (2023), the same model setup (including physics schemes, resolution, and aerosol-cloud interaction modules) was applied to simulate the years 2013 and 2021, which represent China’s air quality evolution. In that work, simulations for both 2013 and 2021 were performed and compared against multiple observational datasets, demonstrating good model performance for key meteorological variables and aerosol concentrations across China. As shown in Table 2 of Gao et al. (2023), the model consistently captured the seasonal and interannual variations of 2-m temperature, relative humidity, and 10-m wind speed, with the Correlation Coefficient (R) for T2 reaching 0.88–0.98 across major Chinese regions in both years. Since GHI is highly sensitive to aerosol loading, the accurate simulation of $PM_{2.5}$ and Aerosol Optical Depth (AOD) is a prerequisite for reliable radiation modeling. Our previous work demonstrated that the model effectively

captures the drastic decline in aerosol concentrations between 2013 and 2021 (Table 3, Gao et al., 2023), with the simulated AOD spatial patterns showing high consistency with MODIS satellite observations. Because surface solar radiation in WRF is calculated through the radiative transfer scheme based on simulated atmospheric states, the accurate representation of these variables provides confidence in the model's ability to simulate surface radiation fields. Therefore, the multi-year evaluation of atmospheric conditions in our previous work, combined with the GHI validation conducted in this study, provides additional support for the reliability of the simulated radiation fields.

Reference:

1. Gao, D., Zhao, B., Wang, S., Wang, Y., Gaudet, B., Zhu, Y., ... & Dong, Z. (2023). Increased importance of aerosol–cloud interactions for surface PM 2.5 pollution relative to aerosol–radiation interactions in China with the anthropogenic emission reductions. *Atmospheric Chemistry and Physics*, *23*(22), 14359-14373.

In general, the paper presents very interesting approaches to the problem, such as considering both the direct and indirect effects of aerosols on solar radiation, as well as implementing a complete modeling chain from atmospheric variables to economic gains.

Some specific comments:

- The code should be made publicly available on GitHub or a similar platform to allow replication of the results.

R: We fully agree with the reviewer that transparency and reproducibility are essential for scientific advancement. In response, we have archived all the necessary resources and source code used in this study to a public repository (available at: https://github.com/shihrong/PV-China-2060), including emission data, namelist, pvlib code, visualization code, and so on.

- The expression "actual conditions" in the text is unclear; please specify what is meant (see for instance line 89 and elsewhere).

R: We thank the reviewer for pointing out this ambiguity. In this study, "actual conditions" refers to the time-varying environmental and atmospheric states simulated by the WRF-Chem model under different carbon-neutrality pathways. Unlike the fixed parameters used in Standard Test Conditions. We have replaced "actual conditions" with "simulated ambient conditions" and "pathway-specific environmental conditions" to ensure clarity and scientific rigor through

the whole manuscript (line 83 and 416)

- PV modeling is performed using the pvlib package; however, the models used at each stage of the process are not clearly described — namely: decomposition into direct and diffuse components, and transposition into the plane-of-array.

R: We thank the reviewer for requesting further technical details on the PV modeling chain. We have now expanded the Methods section to explicitly define the validated sub-models used at each conversion stage:

1. Decomposition: We used the DISC model, which is a standard quasi-physical algorithm for GHI partitioning.
2. Transposition and Optical Losses: We employed the Perez model, which is highly regarded for its ability to handle sky diffuse anisotropy. We further detailed the inclusion of the Xie reflection loss model and the SAPM spectral loss model to enhance the accuracy of the effective irradiance reaching the cells.
3. Temperature and Power: We specified the use of the SAPM cell temperature model and the PVWatts DC power model.

These **detailed descriptions (Line 399-405)** ensure that our modeling pipeline is transparent, reproducible, and aligned with industry-standard meteorological model chains (e.g., Mayer & Yang, 2023).

"

…1) Radiation Decomposition, using the DISC model to partition WRF-Chem GHI into direct and diffuse components; 2) Transposition and Optical Losses, utilizing the Perez model to calculate Plane-of-Array irradiance, while incorporating the Xie model for reflection losses and SAPM for spectral correction; and 3) Power Output Calculation, where cell temperature was estimated via the SAPM temperature model, and final DC power was determined using the PVWatts model…

"

Reference:

1. Mayer, M. J., & Yang, D. (2023). Pairing ensemble numerical weather prediction with ensemble physical model chain for probabilistic photovoltaic power forecasting. *Renewable and Sustainable Energy Reviews*, *175*, 113171.

- Although the approach in this paper is novel, there are previous studies that have evaluated the impact of aerosols and their future changes (natural or anthropogenic) on solar radiation and, consequently, on photovoltaic potential that has not been consider in the manuscript. See,

for instance: Isaza et al. (2023) where an additional effort to see economic impact is being also performed. Boé et al. (2020), Gutiérrez et al. (2020) for the impact of aerosols evolution, and Jerez et al. (2021) for the indirect effect of aerosols.

R: We sincerely thank the reviewer for pointing out these key studies. Incorporating these references has significantly strengthened the theoretical framework of our study and helped situate our findings within the broader context of aerosol-climate-energy research.

We have carefully reviewed these works and integrated them into the Introduction, Line 49-50, line 54-56

Reference:

1. Jerez, S., Palacios-Peña, L., Gutiérrez, C., Jiménez-Guerrero, P., López-Romero, J. M., Pravia-Sarabia, E., & Montávez, J. P. (2021). Sensitivity of surface solar radiation to aerosol–radiation and aerosol–cloud interactions over Europe in WRFv3. 6.1 climatic runs with fully interactive aerosols. *Geoscientific Model Development*, *14*(3), 1533-1551.
2. Gutiérrez, C., Somot, S., Nabat, P., Mallet, M., Corre, L., Meijgaard, E. V., ... & Gaertner, M. Á. (2020). Future evolution of surface solar radiation and photovoltaic potential in Europe: investigating the role of aerosols. *Environmental Research Letters*, *15*(3), 034035.
3. Boé, J., Somot, S., Corre, L., & Nabat, P. (2020). Large discrepancies in summer climate change over Europe as projected by global and regional climate models: causes and consequences. *Climate Dynamics*, *54*(5), 2981-3002.
4. Isaza, A., Kay, M., Evans, J. P., Prasad, A., & Bremner, S. (2023). Air quality impacts on rooftop photovoltaic energy production during the 2019–2020 Australian bushfires season. *Solar Energy*, *257*, 240-248.

Reviewer #4 (Remarks on code availability):

The code should be available in a public repository.

R: We fully agree with the reviewer that transparency and reproducibility are essential for scientific advancement. In response, we have archived all the necessary resources and source code used in this study to a public repository (available at: https://github.com/shihrong/PV-China-2060), including emission data, namelist, pvlib code, visualization code, and so on.

For clarity, revisions made during the **first round** of review are highlighted in **red**, while the newly added amendments in this **second round** of revision are marked in **blue** throughout both the main text and Supplementary Information (SI).

Reviewer 1

We thank the reviewer for the positive assessment and for the constructive comments provided during the previous review round.

1. ***Ensure that all equations in the Methods section are sequentially numbered.***

R: We thank the reviewer for noting this issue. We have carefully checked all equations in the Methods section and ensured that they are sequentially numbered and consistently referenced throughout the manuscript.

2. ***Ensure the GitHub repository (https://github.com/shihrong/PV-China-2060) is set to "Public" and contains a clear README.md with instructions on how to execute the pvlib pipeline and WRF-Chem namelists. Consider minting a DOI for the repository using Zenodo for long-term archiving.***

R: We apologize for the inaccessible repository during the review process. The GitHub repository has now been made publicly available and the access issue has been resolved (https://github.com/shihrong/PV-China-2060.git). We have ensured that the GitHub repository is publicly accessible and added a detailed README describing the workflow, required inputs, and instructions for reproducing the analysis.

We have additionally archived the repository through Zenodo and included the DOI in the Code Availability statement (https://doi.org/10.5281/zenodo.20684741).

The corresponding link has been updated in the manuscript.

3. ***In the Methods section, there is a minor typo: "representing an alternative method to isolate cloud-aerosol forcing as proposed by Ghan's method 62." This should simply read "...as proposed by Ghan (2013)" or similar to avoid redundancy.***

R: Corrected as suggested.

Reviewer 2

We thank the reviewer for the positive assessment and for the constructive comments provided during the previous review round.

1. I hope the github page is not public.. that'swhy showing 404 error.

R: We apologize for the inaccessible repository during the review process. The GitHub repository has now been made publicly available and the access issue has been resolved (https://github.com/shihrong/PV-China-2060.git). We have ensured that the GitHub repository is publicly accessible and added a detailed README describing the workflow, required inputs, and instructions for reproducing the analysis.

We have additionally archived the repository through Zenodo and included the DOI in the Code Availability statement (https://doi.org/10.5281/zenodo.20684741).

The corresponding link has been updated in the manuscript.

Reviewer 3

We thank the reviewer for the positive assessment and for the constructive comments provided during the previous review round.

Reviewer 4

We sincerely thank Reviewer 4 for the constructive comments regarding the robustness of the climate forcing, the representativeness of the simulation period, the evaluation of surface solar radiation, and the accessibility of the modeling framework. These comments have helped us substantially strengthen the manuscript.

Our point-by-point responses are provided below.

1. I believe that the analysis should, at a minimum, include simulations with the RCM driven by multiple forcing datasets. In addition, a 3-years simulation is short to evaluate cloud and aerosols impacts on GHI.

R: We thank the reviewer for these important comments regarding the representativeness of the simulation period and the sensitivity of our results to the choice of climate forcing dataset.

To address these concerns, we conducted two additional sensitivity analyses designed to explicitly evaluate (1) the dependence of our results on the driving climate dataset and (2) the influence of interannual meteorological variability. **First**, we conducted an additional high-resolution WRF-Chem simulation driven by meteorological initial and boundary conditions derived from the independent CMIP6 model MRI-ESM2-0 (Yukimoto et al., 2019), while maintaining the same model configuration and experimental design as in the main simulations. **Second**, to evaluate the influence of interannual variability, we performed additional HR and BS simulations for an independent year (2058) and compared the results with those obtained from the original 2059–2061 simulation period.

The results demonstrate that our main conclusions are highly robust to both climate forcing uncertainty and interannual variability.

(1) Sensitivity to climate forcing dataset

We repeated the HR–BS comparison using MRI-ESM2-0 forcing and compared the resulting changes in GHI and photovoltaic potential (PV_{POT}) with those obtained using the bias-corrected CMIP6 ensemble-mean forcing. The resulting spatial distributions of ΔGHI and ΔPV_{POT} are highly consistent between the two simulations (Figure S7). Quantitatively, the regional mean responses across China's seven power-grid regions are strongly correlated, yielding an R^2 of 0.79 for both variables while preserving identical regional rankings (Figure S8). These results indicate that the projected air-quality-induced solar benefits are not overly sensitive to the choice of driving climate model.

(2) Sensitivity to interannual variability

We further compared the projected changes in GHI and PV_{POT} from the independent year 2058 with the mean response derived from the original 2059–2061 simulations. As shown in

Figure S9, the spatial distributions of both ΔGHI and ΔPV_{POT} remain highly consistent between the two periods. The regional mean responses across China's seven power-grid regions also exhibit strong agreement, with high correlations and nearly identical regional rankings (Figure S10). This consistency demonstrates that both the magnitude and spatial distribution of the projected solar-resource benefits are stable across different years despite meteorological variability.

Importantly, all scenario comparisons in this study focus on relative differences between HR and BS under the same meteorological conditions. Consequently, a substantial portion of meteorological variability is removed when calculating scenario-induced changes. The additional analyses therefore suggest that the projected enhancements in GHI and photovoltaic potential primarily reflect the response to emission reductions rather than artifacts associated with a particular climate model or simulation year.

We acknowledge that a full multi-GCM regional chemistry–climate ensemble and decadal-scale simulations would provide a more comprehensive quantification of uncertainty. However, such simulations remain computationally prohibitive for high-resolution WRF-Chem experiments. Nevertheless, the additional MRI-driven simulation and independent-year experiment provide rigorous sensitivity tests, supporting the robustness of the conclusions presented in this study.

The corresponding analyses have been added to the Supplementary Information (Text S4 and Figures S7–S10).

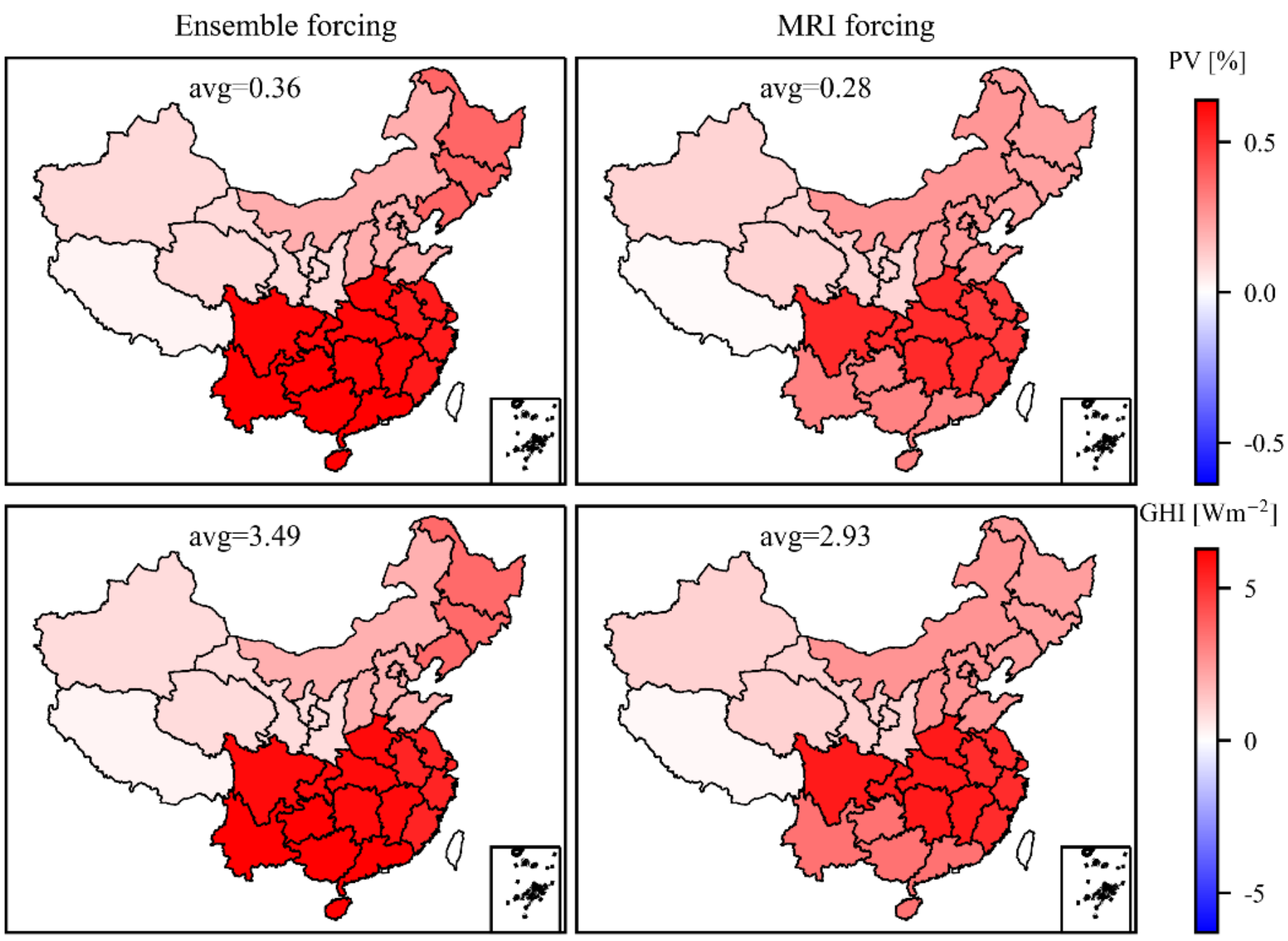

Figure S7. Spatial distributions of changes in global horizontal irradiance (ΔGHI, top panels)

and photovoltaic potential (ΔPV_{POT}, bottom panels) under the HR scenario relative to BS in 2060, simulated using two different meteorological forcing datasets: the 18 CMIP6 models ensemble-mean forcing used in the main manuscript and the independent MRI-ESM2-0 forcing. Despite differences in the magnitude of local responses, both forcing datasets produce highly consistent spatial patterns, with the largest enhancements concentrated in eastern and central China.

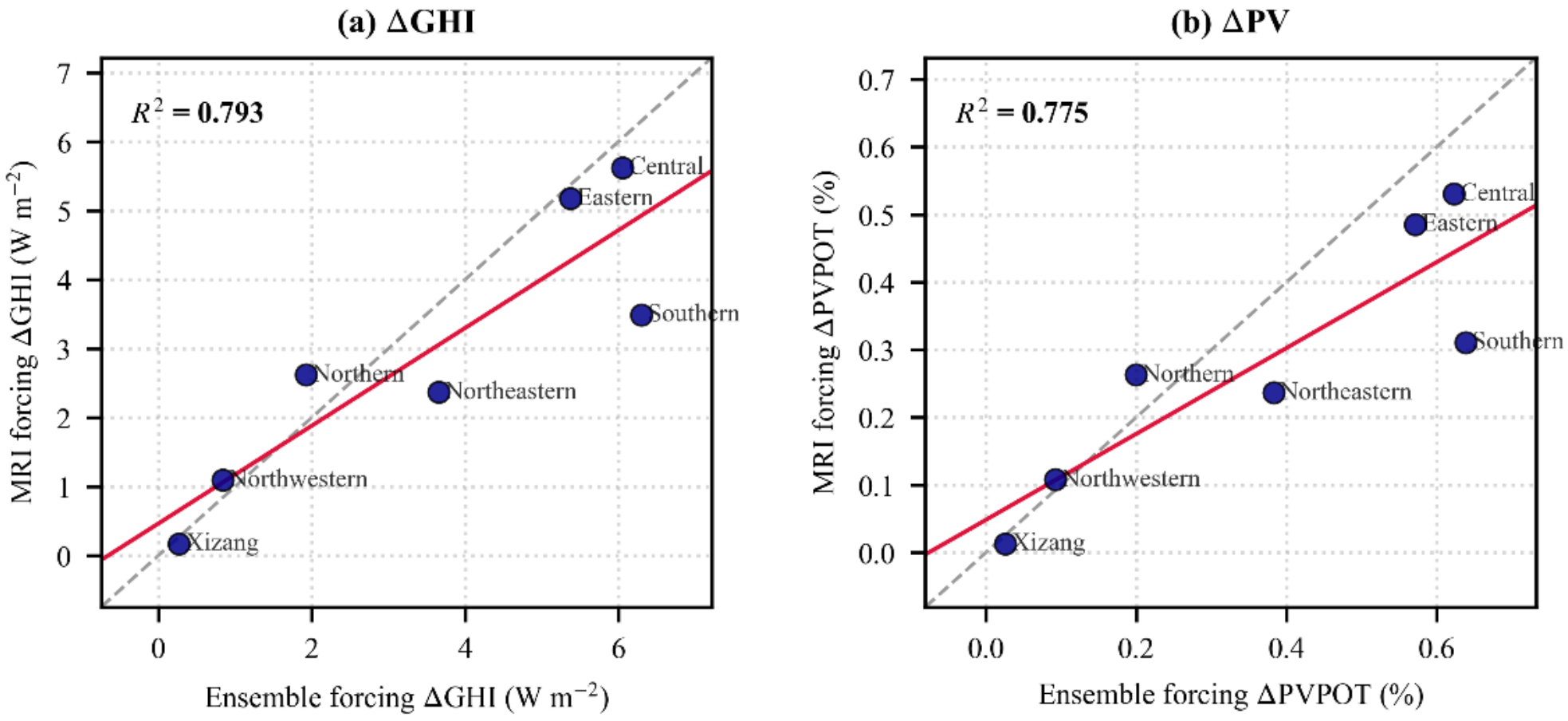

Figure S8. Scatter comparison of Regional mean changes in global horizontal irradiance (ΔGHI) and photovoltaic potential (ΔPV_{POT}) under the HR scenario relative to BS simulated using the CMIP6 ensemble-mean forcing and MRI-ESM2-0 forcing. Each point represents one of China's seven regional power grids. Strong correlations between the two simulations ($R^2 = 0.79$ for both ΔGHI and ΔPV_{POT}) indicate that the projected air-quality benefits for solar resources and PV performance are largely insensitive to the choice of meteorological forcing dataset.

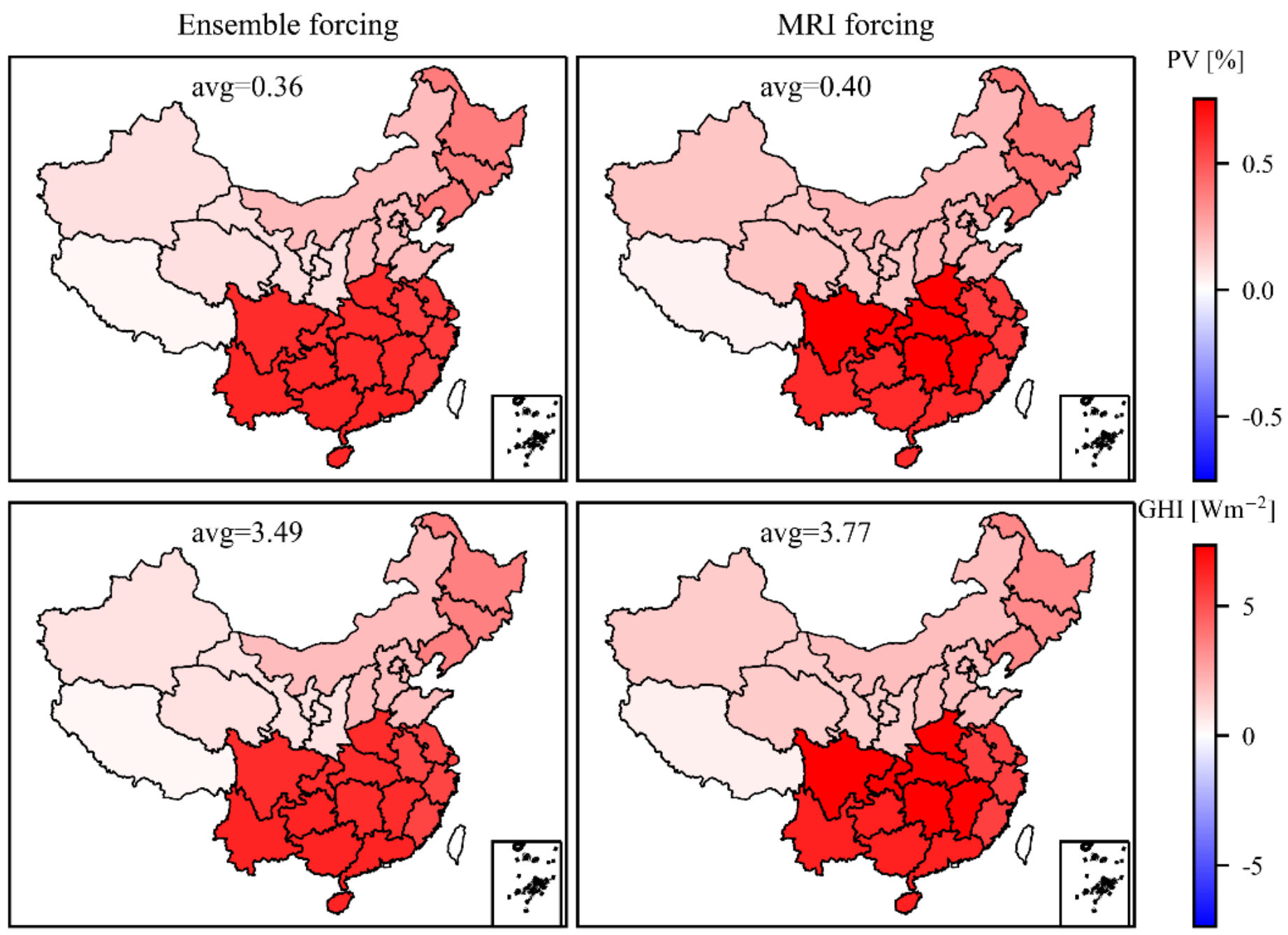

Figure S9. Spatial distributions of changes in global horizontal irradiance (ΔGHI, top panels) and photovoltaic potential (ΔPV_{POT}, bottom panels) under the HR scenario relative to BS in 2060, derived from the original simulation period (2059–2061 mean) and an independent year (2058). Similar spatial structures indicate limited sensitivity to interannual variability.

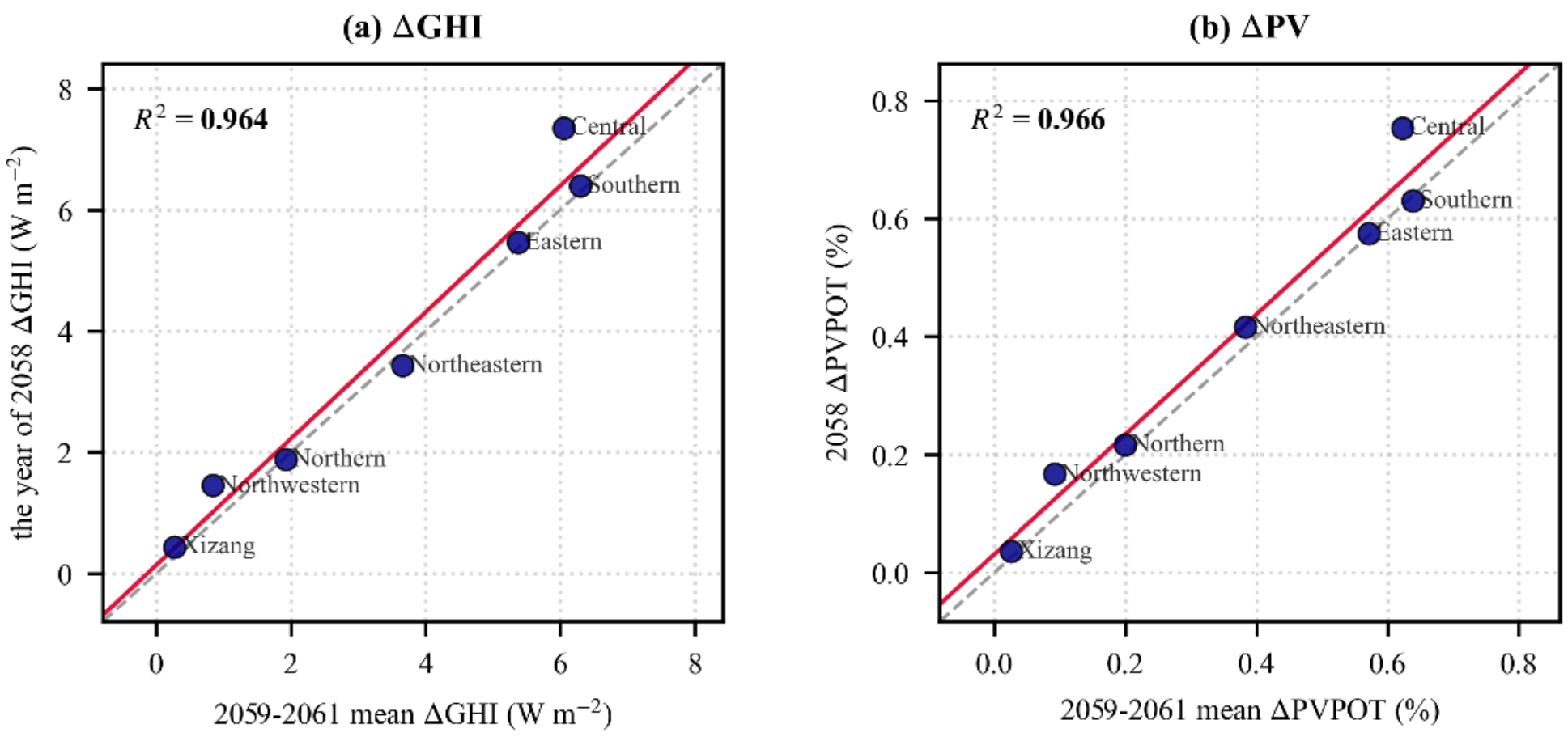

Figure S10. Scatter comparison of Regional mean changes in global horizontal irradiance (ΔGHI) and photovoltaic potential (ΔPV_{POT}) under the HR scenario relative to BS between 2058 and the 2059–2061 mean. The high correlation demonstrates that the projected enhancement is robust to interannual variability.

“

Text S4. Robustness of projected GHI and photovoltaic potential enhancements to climate forcing and interannual variability

To evaluate the robustness of the projected air-quality-induced enhancements in surface solar radiation and photovoltaic potential, we conducted two complementary sensitivity analyses. First, to assess the influence of climate forcing uncertainty, an additional set of simulations was performed using meteorological boundary conditions derived from the MRI-ESM2-0 model (Yukimoto et al., 2019), while the main simulations employed the bias-corrected ensemble mean of 18 CMIP6 models. Second, to examine the impact of interannual variability, we conducted additional simulations for 2058 under both the HR and BS scenarios and compared the results with the original 2059–2061 simulations.

The resulting spatial distributions of ΔGHI and ΔPV_{POT} are highly consistent with those obtained using the CMIP6 ensemble-mean forcing (Figure S7). Quantitatively, the regional mean responses across China's seven power-grid regions are strongly correlated between the two simulations, yielding an R^2 of 0.79 for both variables and preserving identical regional rankings (Figure S8). This suggests that the ensemble-mean forcing adopted in the main analysis provides a representative estimate of future meteorological conditions.

The resulting spatial distributions of ΔGHI and ΔPV_{POT} in 2058 closely resemble those obtained from the 2059–2061 mean simulations (Figure S9). Quantitatively, the regional mean responses across China's seven power-grid regions are strongly correlated between the two periods, with R^2 values of 0.96 for ΔGHI and 0.96 for $\Delta PV_{POT,}$ and with the regional ranking of projected benefits remaining unchanged (Figure S10). The consistency in both spatial patterns and regional responses demonstrates that the projected air-quality-induced enhancements are robust to interannual meteorological variability.

Taken together, these sensitivity analyses demonstrate that the projected air-quality-induced improvements in GHI and photovoltaic potential are robust to both climate forcing uncertainty and interannual variability, supporting the reliability of the conclusions presented in the main manuscript.

"

Reference:

Kawai, H., Yukimoto, S., Koshiro, T., Oshima, N., Tanaka, T., Yoshimura, H., and Nagasawa, R.: Significant improvement of cloud representation in the global climate model MRI-ESM2, Geosci. Model Dev., 12, 2875–2897, https://doi.org/10.5194/gmd-12-2875-2019, 2019.

2. ***Evaluation of GHI was made for a very short period. I understand that it was previously made in another work, but still needed.***

R: We thank the reviewer for this valuable suggestion. We agree that, because GHI is the primary meteorological variable driving the photovoltaic performance calculations in this study, a dedicated evaluation of simulated GHI is necessary.

In the original manuscript, we primarily relied on the evaluation of aerosol-related variables reported in Gao et al. (2025), which employed the same WRF-Chem configuration and demonstrated reasonable model performance for aerosol concentrations and optical properties. Following the reviewer's suggestion, we conducted an additional direct evaluation of simulated GHI using ground-based observations for two representative years, 2013 and 2021.

The evaluation was performed over the seven regional power grids used throughout this study. Results show that the model reproduces the observed spatial and regional variations of daily GHI reasonably well in both years. For 2013, the national mean relative mean bias (rMBE) and relative root mean square error (rRMSE) are 12.4% and 33.6%, respectively. For 2021, the corresponding values are 10.4% and 32.2%. Importantly, model performance remains highly consistent between the two years despite the substantial changes in air pollution levels that occurred in China during the intervening decade.

These results provide additional confidence that the radiation simulations generated by the WRF-Chem configuration used in this study are able to realistically represent surface solar radiation across a wide range of atmospheric conditions. We have therefore added a new supplementary figure and table presenting the GHI evaluation results and have updated the manuscript accordingly.

Added text in the Supplementary Information:

"Table S3 presents the evaluation of simulated daily GHI against ground-based observations for 2013 and 2021. Across the seven regional power grids, WRF-Chem reproduces the observed spatial variability of GHI reasonably well. Nationally averaged rMBE values are 12.4% and 10.4% for 2013 and 2021, respectively, while corresponding rRMSE values are 33.6% and 32.2%. The similar performance across the two years suggests that the model provides a stable representation of surface solar radiation under substantially different aerosol pollution conditions."

3. The code is not available, or I have not been able to access.

R: We apologize for the inaccessible repository during the review process. The GitHub repository has now been made publicly accessible and the access issue has been resolved (https://github.com/shihrong/PV-China-2060.git). We have ensured that the GitHub repository is publicly accessible and added a detailed README describing the workflow, required inputs, and instructions for reproducing the analysis.

We have additionally archived the repository through Zenodo and included the DOI in the Code Availability statement (https://doi.org/10.5281/zenodo.20684741).

The corresponding link has been updated in the manuscript.

The authors have presented a study addressing a highly relevant topic at the intersection of carbon neutrality, air quality, and solar energy, with a focus on one of the most polluted and climate-sensitive regions, China. Overall, the manuscript is well written; however, I have the following comments.

1. The **abstract** is long. Should be trimmed in accordance with Nature journal.

Introduction

2. What are the **2060 carbon-neutral pathways** referred to in the study? Are these scenarios specifically developed for **China using C-GEM**, or do they represent global trajectories? It would be helpful to illustrate this with a brief description of the corresponding **emission reduction pathways and its difference w.r.t BS** scenario. Although these scenarios might have been developed in earlier studies, including a concise discussion in the **Methods** section (or Supplementary Information) would enhance the completeness and standalone clarity of the paper (just like Table 1 or spatial map).

3. Additionally, although the study focuses on **China**, considering the broader readership of *Nature*, it would strengthen the paper to reference relevant literature from the **Global South**—particularly from **India**, where similar air pollution challenges exist and studies have demonstrated that **clean air significantly enhances solar energy production**. Incorporating this perspective (e.g., around lines 49–50) would help emphasize the **global relevance and broader significance** of the work.

4. **Line 75–80:** The main findings of the study are currently presented in the *Introduction*. It would be more appropriate to move these details to the *Results* section and instead include a few concise sentences in the Introduction highlighting the **innovation** of the paper.

5. Line 73-80: HR scenario will bring more PV power and that too 2.8 times higher than HB and double to CS. clarify whether this increment comes only from air quality improvement or from projected PV installation (Table 1) or from both the effect. Authors provide the methodology Line 423 (Estimation of **aerosol-induced** power generation increase). Also need to look-into the “aerosol-induced” phrase.

Results:

6. **Line 143-145:** Could the authors clarify why the Xizang and Northwestern regions are expected to show only a minor positive impact of T2 and wind speed (WS) on PV potential? A brief explanation—perhaps linked to regional climatic conditions, topography, or atmospheric circulation patterns—would help readers better understand the underlying physical rationale for these results.

7. I appreciate the authors' effort to connect changes in **clear-sky GHI** with aerosol-induced cloud impacts and I see authors use the following:

   ΔC = Δ(F_all − F_clear)
   where *F_all* is the shortwave radiative flux at the TOA (SFC) under all-sky conditions and *F_clear* is the flux diagnosed with clouds neglected. Here, Δ: represents the difference between the various experiments (e.g., HR, HB, and CS relative to BS). According to Ghan (2013), Δ typically denotes the difference between atmospheric simulations with and without anthropogenic emissions, while maintaining identical ocean and natural emission conditions.)

   Ghan (2013) further proposed that a more realistic attribution involves
   ΔC_clean = Δ(F_clean − F_clear,clean),
   where *F_clean is the* diagnostic flux neglecting aerosol scattering and absorption, and *F_clear,clean* is the diagnostic flux neglecting both cloud and aerosol scattering and absorption. Δ: defined already.

   The difference between these two approaches– one with *all-sky and clear-sky vs. clean-sky and clean-clear-sky* diagnostics—was shown to produce a **non-negligible signal**, particularly for regions such as **China and India**, where aerosol–cloud interactions are strong. Therefore, the current claim of aerosol-induced cloud impacts on GHI changes may not be fully supported without including **clean-sky and clean-clear-sky diagnostics**. Incorporating these diagnostics would provide a **more robust quantification** of aerosol effects, and could potentially alter the number and (interpretations ?) in **Lines 177–179**, even though signals in **LWP** and **cloud droplet number concentration** are evident.

   Reference : Ghan (2013) https://acp.copernicus.org/articles/13/9971/2013/

8. **Line 183-186:** The assumption of an average of 1,500 operational hours should be supported with a relevant reference or data source, as this parameter directly influences subsequent estimates of PV generation and associated economic benefits. Providing a citation—ideally from national energy statistics, technical reports,

peer-reviewed studies or your rationale—would strengthen the credibility and practical basis of this assumption.

9. **Line 185–187:** "Multiplied the **aerosol-induced** PV power increase by 1,500 hours to obtain the annual power generation. Figure 4a illustrates the aerosol-induced PV power generation..." The claim of *aerosol-induced* is not appropriate, because the authors use various emission-driven scenarios that obviously improve air quality but also bring changes in cloud properties (COD, Fig. 3, and also Supplementary). Therefore, the aerosol emission reductions in HR, HB, and CS pathways change GHI and PV potential by modulating aerosols, clouds, and other meteorological parameters. Hence, claiming only *aerosol-induced* PV potential change is not conclusive. For that, the authors need additional diagnostics stated in *Comment 6*, or they could focus only on how much change they observe in GHI_clear (which mainly depends on aerosols). A better phrase could be "aerosol-emission reduction pathways driven" / "carbon-neutrality pathways driven" (which are stated in Line 202). Change in phrase is also necessary in **Figure 4** also.

10. **Line 353:** How sensitive is the **pvlib** package when decomposing direct and diffuse radiation using only solar position? How confident are the authors about the **diffuse component**, which is more sensitive to **aerosol type** and **cloud properties**, whereas the **direct component** is comparatively less sensitive to these factors? This decomposition is further utilized in the **POAI calculation** (Lines 370–371).

11. **Line 383: Contribution of GHI, T, and WS on PVPOT:** This section contains descriptive text. The authors should refer to the existing literature where a detailed mathematical formulation of these contributions has already been presented.

    Reference:

    https://iopscience.iop.org/article/10.1088/1748-9326/ac2a64/meta

    https://iopscience.iop.org/article/10.1088/1748-9326/ad8c68

Methods:

12. A map showing 7 power grids in the supplementary is encouraged.

13. What is the horizontal resolution of the WRF simulation? Although the authors have used a previously published WRF setup, providing a **few key details of the model**

configuration (e.g., domain size, resolution, nesting strategy, and physics schemes) would make the current paper more self-contained and comprehensible as a standalone read.

14. Why only SSP126 climate data used for future scenarios? (Line 337) Provide your rationale why other SSPs will not qualify towards the Carbon neutrality target for China.
15. It is observed that, both AOD and COD decreases in all scenarios w.r.t Baseline scenario. Are AOD and COD enough to establish the aerosol-cloud interaction?
16. It is observed that the authors provide **uncertainty quantification** primarily associated with the **WRF simulations**. However, additional uncertainties may also arise from the **emission trajectory models**, **pvlib-based calculations**, the **economic assessment**, and several underlying **assumptions**. It is therefore encouraged to include a brief discussion in the **Methods** or **Supplementary Information** to illustrate and acknowledge these sources of uncertainty. This addition would also enhance the **policy relevance** of the study, and the authors are encouraged to comment on its **global applicability** and **reproducibility** for other regions with similar contexts or interests.
17. (Minor) Provide units in the table in the supplementary.
